# Ketene as a gas phase intermediate in acetic acid ketonization on TiO_2_

**DOI:** 10.1038/s41467-026-77945-8

**Published:** 2026-10-28

**Authors:** Jiayan Xu, William JI DeBenedetti, Xingyu Wang, Yiqing Wu, Libor Kovarik, Christopher R. O’Connor, Feng Gao, Janos Szanyi, Yong Wang, Annabella Selloni, Greg A. Kimmel, Zdenek Dohnálek

**Affiliations:** 1 Department of Chemistry, Princeton University, Princeton, NJ USA; 2 Physical and Computational Sciences Directorate and Institute for Integrated Catalysis, Pacific Northwest National Laboratory, Richland, WA USA; 3 Voiland School of Chemical Engineering and Bioengineering, Washington State University, Pullman, Washington, USA; 4 Present Address: Plutonium Supply & Disposition (PSD-4), Los Alamos National Laboratory, Los Alamos, New Mexico USA; 5 Present Address: Commonwealth Fusion Systems, Devens, MA USA

**Keywords:** Catalytic mechanisms, Heterogeneous catalysis, Surface spectroscopy

## Abstract

Reactive gas-phase intermediates are often underappreciated in heterogeneous catalysis, despite their potential to control key catalytic steps. Here, we provide direct experimental evidence that ketene can act as an intermediate in acetic acid ketonization, a mechanism that has long been debated. Using faceted TiO_2_(101) nanoparticles assembled into layers of controlled thickness, we show that ketene, formed from surface acetate, migrates through interparticle voids via desorption-readsorption events before reacting with surface acetate to yield acetone. Surface science and reactor studies reveal a direct correlation between ketene and acetone production and the depth to which the particle layers are saturated. Ab initio calculations support ketene’s reactivity via low-barrier coupling pathways that converge on a common β-ketoacid intermediate. Here, we show that ketene can act as a mobile gas-phase intermediate and underscore the broader importance of spatially decoupled elementary steps in determining reactivity and selectivity in catalytic systems.

## Abstract (web summary)

Reactive gas-phase intermediates are often overlooked in heterogeneous catalysis despite their critical role in governing catalytic pathways. Here, the authors provide direct experimental evidence that ketene serves as a key intermediate in acetic acid ketonization, resolving a long-standing mechanistic debate.

## Introduction

Reactive gas-phase intermediates generated at catalyst surfaces are often overlooked despite their potential to control key catalytic steps. Surface-initiated, transport-mediated chemistry shows that the sites of intermediate formation and consumption can be spatially decoupled^1–5^. Motivated by this paradigm, we revisit ketonization, the coupling of two carboxylic acids into a ketone with the release of carbon dioxide and water, as a key route for upgrading biomass-derived feedstocks into valuable chemicals and fuels^6–8^. A central mechanistic question is whether ketene (CH_2_ = C = O), formed by C–O bond cleavage of a surface enol, can function as a reactive gas-phase conduit to acetone, or it is a low-yield side product with no effect on the production of acetone.

Metal oxide catalysts with amphoteric Lewis acid–base pairs, such as TiO_2_, CeO_2_, and ZrO_2_, are particularly effective for ketonization due to their ability to activate carboxyl groups^9–14^. The prevailing mechanistic framework for the ketonization reaction involves the formation of a β-ketoacid intermediate, produced by C–C coupling between an enolate species and an adjacent monodentate carboxyl group, which subsequently decarboxylates to yield the ketone^9,10^. Furthermore, facet-dependent studies of anatase TiO_2_ suggest that the monodentate binding configuration of carboxylic acids is likely the more reactive intermediate toward C–C coupling^14^. Although density functional theory (DFT) calculations consistently support the feasibility of this mechanism, direct spectroscopic detection of β-ketoacid intermediates has not been achieved experimentally. As an alternative route, it has been suggested that ketene, generated via monomolecular dehydration and decomposition of surface enolates, might participate as an intermediate. Ketene formation has been experimentally observed under ultra-high vacuum (UHV) conditions across various metal oxide surfaces^15–17^. However, its role in the catalytic formation of acetone has remained controversial due to both its typically low yield at higher, more catalytically relevant pressures (≥1 Torr) and the lack of clear experimental evidence for its direct participation in the catalytic cycle^18^.

Skepticism regarding ketene’s catalytic relevance stems largely from an isotopic labeling study involving cross-ketonization of ^13^C-labeled acetic acid and unlabeled pivalic acid^19^. In that work, ^13^C was exclusively detected in the carbon dioxide product rather than in the ketone, suggesting ketene formation is a parallel, nonparticipating pathway rather than an intermediate step in ketone formation. Further complicating the mechanistic picture is the identification of anhydride species during ketonization on ZrO_2_ aerogels^20^ and CeO_2_ catalysts^13^, which suggests another possible reaction pathway. These anhydrides, however, have not been broadly observed on metal oxides, and their role remains ambiguous, despite theoretical predictions of their relevance on zeolites^21^.

Here, we use nanoparticulate anatase TiO_2_(101), assembled into supported layers of varying thickness, to probe whether ketene can participate in the reaction mechanism of acetic acid ketonization. Unlike planar single crystals, voids in such layers enable interparticle transport. Temperature-programmed reaction spectroscopy (TPRS) and reactor studies reveal that ketene, generated within deeper layers, can travel through the interparticle voids and serve as a gas-phase intermediate that reacts with acetates on neighboring nanoparticles to form acetone. DFT calculations support this mechanism, showing that ketene readily couples with surface enolates or monodentate acetic acid to form β-ketoacid intermediates. However, we emphasize that our UHV experiments are designed to isolate elementary steps and transport effects rather than reproduce the full steady-state populations of surface species. Under more realistic conditions, the relative populations of bidentate acetate, monodentate acetate/acetic acid, enolate species, and weakly adsorbed intermediates may differ from those in the temperature-programmed experiments. As such, our results do not rule out the classical β-ketoacid mechanism or establish ketene as the only acetone-forming intermediate. Rather, they demonstrate that ketene-mediated C–C coupling is a viable pathway for the ketonization of acetic acid.

## Results

### Characterization of supported faceted TiO_2_(101) layers

**Fig. 1:**
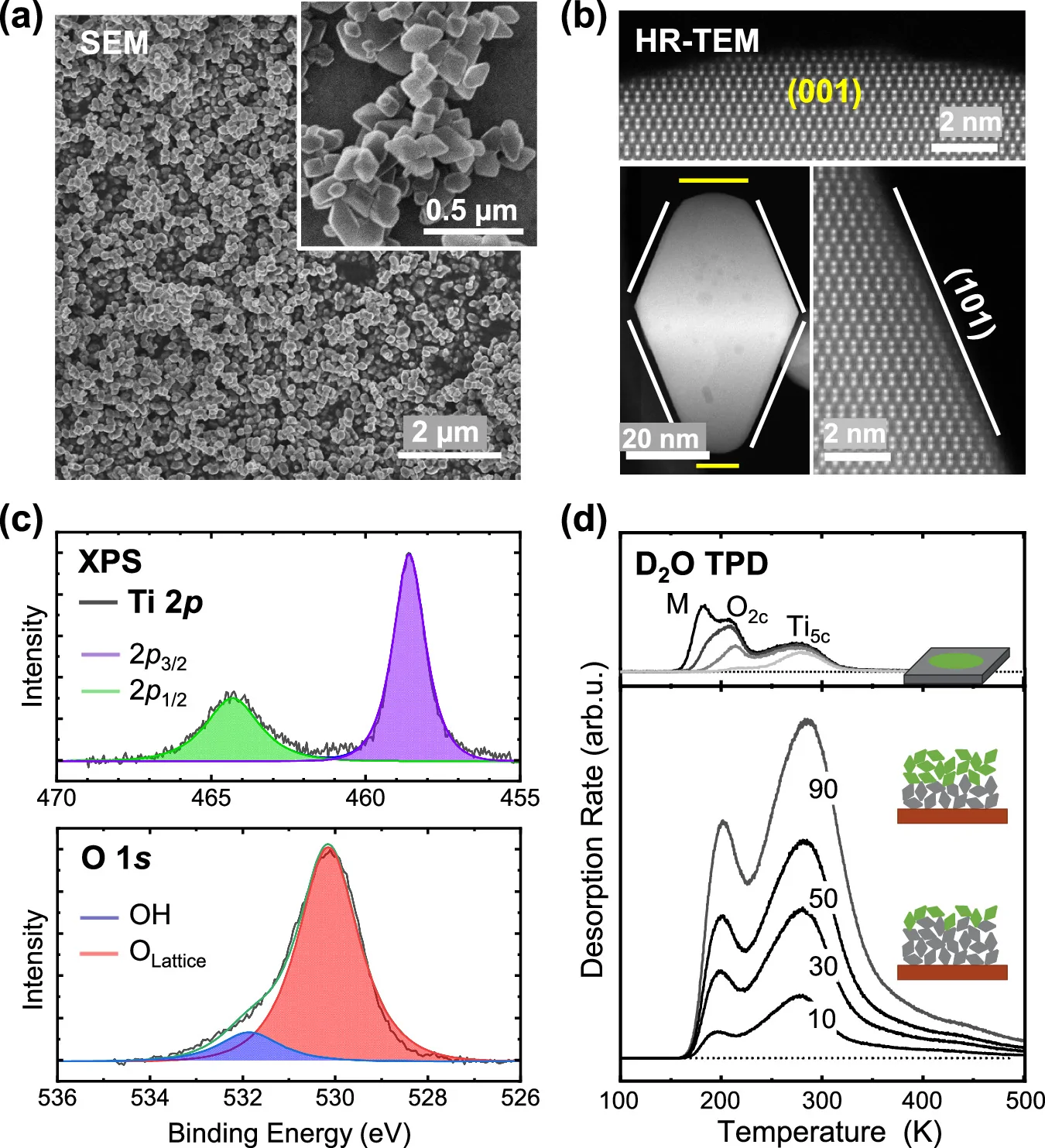
Structural and spectroscopic characterization of TiO_2_(101) nanoparticles supported on Au-coated Ta foil. **a** A scanning electron microscopy (SEM) image showing the architecture of the nanoparticulate layer. Inset: High-resolution He ion microscopy reveals particles with dominant (101) facets. **b** A high-resolution transmission electron microscopy (HRTEM) image of a representative nanoparticle reveals a bipyramidal shape with dominant (101) surfaces and minority stepped (001) terminations. **c** X-ray photoelectron spectroscopy (XPS) spectra from the annealed nanoparticulate TiO_2_(101) layer. Top: Ti 2*p* spectrum shows two Ti^4+^ spin–orbit peaks at 459.3 eV (2*p*_3/2_) and 465.0 eV (2*p*_1/2_), with no evidence of reduced Ti species. Bottom: O 1 *s* XPS spectrum shows a dominant lattice oxygen peak at 530.4 eV and a shoulder at 531.9 eV attributed to surface hydroxyls. **d** Dose-dependent D_2_O temperature-programmed desorption (TPD) spectra from the planar TiO_2_(101) single crystal (top) and the “thick” nanoparticulate TiO_2_(101) layer (bottom). Desorption peaks correspond to Ti_5c_ (250–350 K), O_2c_ (180–230 K), and multilayer (M, ~175 K) adsorption. The Ti_5c_ peak area from the planar crystal defines 1 monolayer (ML) and serves as a reference for quantifying D_2_O dose equivalents on the nanoparticulate TiO_2_(101) layer. Insets: circular adsorption spot (green) on the planar surface (top), and schematic illustration of progressive layer filling by adsorbate (green, bottom: top-only filled and middle: filled halfway).

A series of samples consisting of nanoparticulate TiO_2_(101) layers of varying thickness was prepared by spray- and drop-casting aqueous suspensions onto Au-plated optically polished Ta substrates (see “Methods” for details). The nanoparticles themselves were synthesized via the hydrothermal synthesis method^22^. Scanning electron microscopy (SEM) imaging reveals that the resulting films consist of well-dispersed, loosely packed nanoparticles (Fig. 1a), with diameters ranging from 100 to 200 nm, consistent with previous reports^23,24^. X-ray diffraction and Raman spectroscopy confirm the high crystallinity and phase purity of the anatase phase before and after reactivity studies (Supplementary Fig. 1 and Supplementary Fig. 2). High-magnification He ion microscopy (inset, Fig. 1a) shows that the particles exhibit a bipyramidal morphology. In some regions, the flat Au substrate remains visible, indicating incomplete coverage. High-resolution transmission electron microscopy (HR-TEM) imaging (Fig. 1b) further confirms a truncated square bipyramidal shape with some internal pores visible as dark contrast. The exposed surface is dominated by flat (101) facets, which account for ~90% of the total surface area; minority (001) facets exhibit slight curvature due to steps and edge terminations.

We used X-ray photoelectron spectroscopy (XPS) to characterize the surface composition of the faceted TiO_2_(101) nanoparticles after annealing to 850 K in ultra-high vacuum (UHV). The Ti 2p spectrum (Fig. 1c, top) shows two characteristic peaks at 459.3 eV and 465.0 eV, corresponding to the Ti 2*p*_3/2_ and Ti 2*p*_1/2_ transitions of fully oxidized Ti^4+^. No evidence of Ti^3+^ species is observed; such features would appear ~2 eV lower in binding energy relative to the Ti 2*p*_3/2_ peak^25^. Trace bulk vacancies cannot be excluded without electron paramagnetic resonance (EPR), which would be a useful complementary measurement.

The O 1*s* spectrum (Fig. 1c, bottom) exhibits a dominant peak at 530.4 eV, attributed to lattice oxygen, and a smaller shoulder at 531.9 eV assigned to surface hydroxyl groups^26,27^. The relative intensity of the OH feature suggests that surface hydroxyls account for ~15% of the total oxygen signal following annealing. Compared to the as-prepared sample, the hydroxyl peak is reduced by approximately half (Supplementary Fig. 3), consistent with thermal removal of surface OH at elevated temperature.

The XPS and D_2_O TPD structural characterization presented below shows no evidence for a significant concentration of surface oxygen vacancies and strongly suggests that the surfaces of faceted anatase TiO_2_(101) nanoparticles should be structurally analogous to the surface of the anatase TiO_2_(101) single crystal commonly used in surface science. However, their faceted morphology introduces a higher surface area and distinct structural features that are not present in planar samples. To quantify the surface area enhancement within the layers relative to a single crystal, we performed adsorption titration experiments using water as a probe molecule^28,29^. D_2_O was delivered via a molecular beam to ensure controlled dosing onto the TiO_2_ surface. To suppress multilayer adsorption on the nanoparticles and to avoid adsorption on exposed Au regions, the dosing was carried out at ~175 K^26,30^. This approach allows us to capture the complete evolution of all desorption features from the monolayer (ML) to multilayer with a subsequent temperature-programmed desorption (TPD) experiment (Fig. 1d).

D₂O TPD spectra from the single crystal TiO_2_(101) as a function of D_2_O dose are shown in the top panel of Fig. 1d. Consistent with previous studies^26,30^, the fully saturated surface (black curve) exhibits three distinct desorption peaks centered at ~280, 215, and 175 K. These features are attributed to D_2_O bound at progressively weaker sites starting from undercoordinated Ti_5c_ sites, twofold-coordinated bridging oxygen sites, O_2c_, and multilayer water, respectively. We define the saturation coverage of water on Ti_5c_ sites, corresponding to the ~280 K peak, as one monolayer (ML) and use it as a reference for quantifying surface site densities. The 215 K peak emerges only after Ti_5c_ sites are saturated (1.6 and 2.4 ML doses, medium gray curves), indicating weaker adsorption on O_2c_ sites. At higher coverage (3.2 ML), a shoulder at ~175 K appears, consistent with the onset of water multilayer formation^26,30^.

It should be noted that no additional high-temperature desorption peaks attributable to defect-associated water is resolved beyond the molecular-water features^30,31^. This absence indicates that the planar TiO_2_(101) used here contains no significant concentration of oxygen vacancies. (A more detailed set of coverage-dependent spectra is shown in Supplementary Fig. 4.) This assertion is also consistent with the absence of a reduced Ti^3+^ feature for the faceted nanoparticles (Fig. 1c) that would appear as a shoulder in Ti 2*p* XPS.

The corresponding D_2_O TPD spectra for the nanoparticulate TiO_2_(101) layer are shown in the lower panel of Fig. 1d. Across all doses (10–90 ML_eq_, expressed in ML equivalents relative to planar TiO_2_(101)), the spectra exhibit two prominent desorption peaks centered at ~280 and ~200 K, both increasing in intensity with dose. These features correspond to the Ti_5c_ and O_2c_ adsorption sites identified on the planar TiO_2_(101) surface (Fig. 1d, top). We note the absence of saturation even at the highest dose (90 ML_eq_), referred to as the “thick” layer below, confirms that the surface area of the nanoparticulate TiO_2_(101) layer is substantially greater than that of the planar single crystal.

The area of the Ti_5c_ peak is consistently larger than that of the O_2c_ peak across all doses, which we attribute to the elevated dosing temperature (~175 K) used for the nanoparticulate TiO_2_(101) layers compared to ~100 K for the single crystal. As with the planar surface, no desorption features are observed above 280 K, indicating that there is no significant concentration of surface defects such as oxygen vacancies on the faceted nanoparticles. Furthermore, no desorption peak is observed near ~175 K, indicating that water multilayers do not form.

The behavior of the thick TiO_2_(101) layer contrasts with that of two additional samples containing progressively thinner nanoparticulate TiO_2_(101) layers (Supplementary Fig. 5). These two thinner samples approach a saturation coverage at considerably lower D_2_O doses of ~20 and ~10 ML. Below, we refer to these as the “medium” and “thin” layers, respectively.

At low doses, where adsorption occurs primarily on the top layers of all films, the D_2_O TPD spectra from the thin, medium, and thick nanoparticulate TiO_2_(101) layers are similar. At higher doses, however, the thin and medium films approach saturation, whereas the thick film continues to take up additional D_2_O. If D_2_O adsorption were limited to the outermost particles exposed to the molecular beam, the thick film would also saturate once this external surface area was filled. Instead, the continued uptake by the thick film indicates that additional TiO_2_ surface area becomes accessible at higher doses. Because the desorption features remain associated with D_2_O bound to Ti_5c_ and O_2c_ sites and no multilayer water feature is observed near ~175 K, the increased uptake is not attributed to condensation of water on the external surface. Rather, the dose- and thickness-dependent uptake is consistent with D_2_O transport into deeper regions of the nanoparticulate film, likely through a combination of surface diffusion, desorption-readsorption, and gas-phase transport through interparticle voids^32^.

This behavior contrasts with that of the planar single crystal (Fig. 1d, top), where adsorption follows a site-specific sequence: Ti_5c_ sites saturate first, followed by O_2c_ sites and eventually multilayer formation, indicating that diffusion of water molecules on the surface allows them to find the most strongly binding sites that are available. The limited contact points between the nanoparticles (see Fig. 1a) inhibit surface diffusion between them, thus preventing significant transport via this surface-only route.

Based on simple geometric considerations and the bipyramidal morphology of the nanoparticles, we estimate that a single, randomly oriented layer of nanoparticles has ~4.3 times the area of the planar TiO_2_(101) (see Supplementary Note 1). Accordingly, a dose of 90 ML is sufficient to saturate approximately 21 layers within the “thick” film. By comparison, the saturation doses for the “medium” and “thin” samples correspond to roughly 4 and 2 layers of TiO_2_(101) nanoparticles, respectively.

The comparison with planar TiO_2_(101) is central to the interpretation of these experiments. The D_2_O TPD results show that the faceted nanoparticles expose TiO_2_(101)-like adsorption sites, as indicated by desorption features associated with Ti_5c_ and O_2c_ sites. However, the assembled nanoparticle layers differ from planar TiO_2_(101) in two important ways. First, they provide a much larger accessible surface area distributed through multiple particle layers. Second, the interparticle voids create a three-dimensional transport network in which weakly bound or volatile species can undergo desorption-readsorption events and collide with neighboring particle surfaces before escaping into the vacuum. Thus, the nanoparticle layers retain the local surface chemistry of TiO_2_(101), while introducing transport and spatially separated reaction steps that are absent on a planar single crystal. In this respect, the nanoparticle layers more closely match the conditions encountered in real catalytic reactors than the single crystal experiments.

### Competition between dehydration and ketonization

As shown above, the faceted TiO_2_(101) nanoparticles expose the same dominant adsorption sites as the planar TiO_2_(101) surface. However, previous research has shown that the reaction of acetic acid yields markedly different products on the two systems: on planar surfaces, ketene forms predominantly via monomolecular dehydration^15,33^, whereas on nanoparticles, acetone is the primary product, formed through bimolecular C–C coupling^34,35^. The supported layers of faceted TiO_2_(101) used in this study provide an ideal platform for probing the mechanistic origins of these distinct reaction pathways.

The vibrational frequencies revealing the binding of acetic acid on planar and nanoparticulate TiO_2_(101) in infrared reflection absorption spectroscopy (IRRAS) spectra are further shown in Supplementary Fig. 6. There, dosing at very low temperature (<100 K) leads to the molecular acetic acid layer and subsequent annealing above 150 K to deprotonation and bidentate acetate formation^15^.

**Fig. 2:**
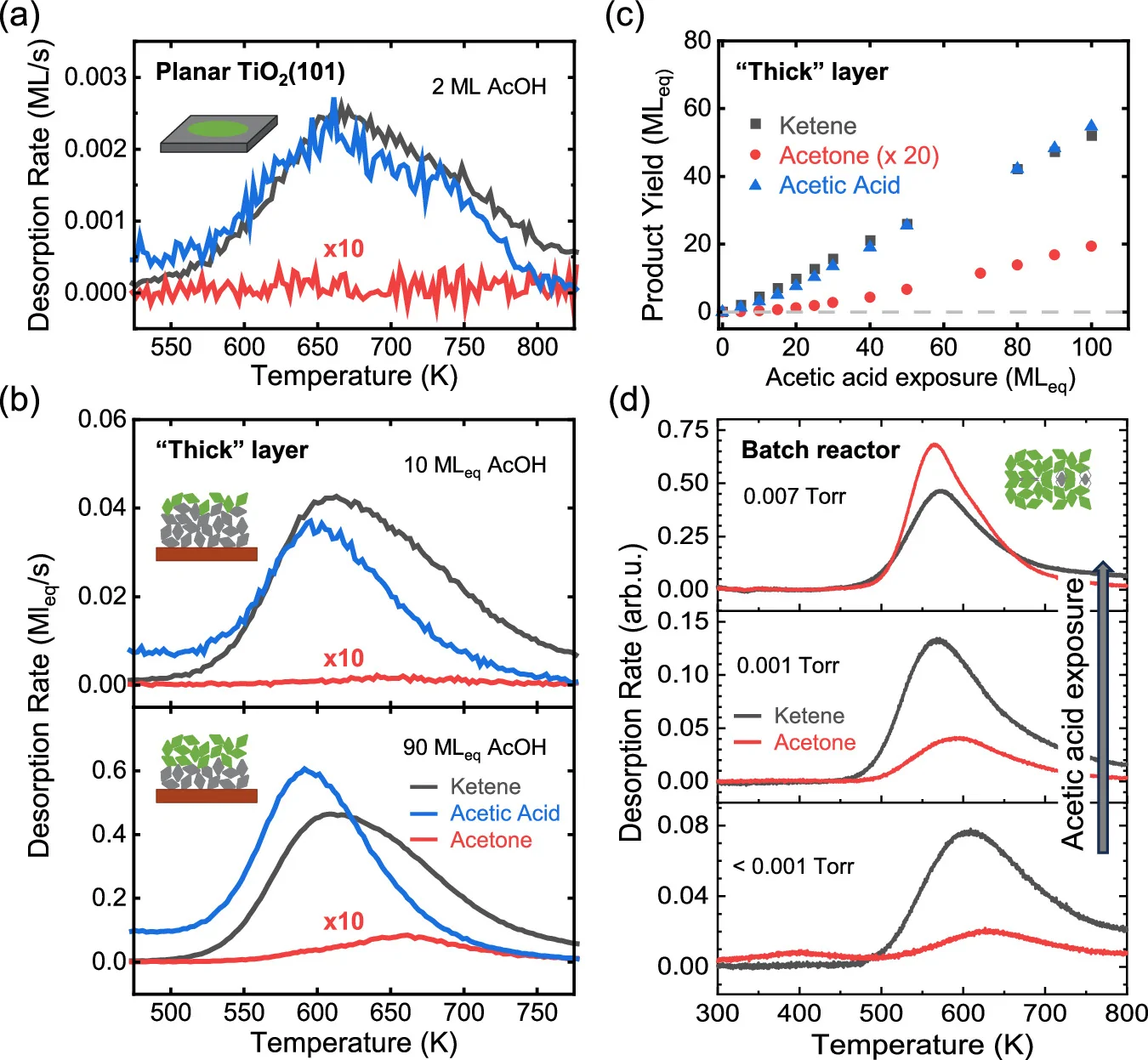
Temperature-programmed reaction spectroscopy (TPRS) and product yields for acetic acid (AcOH) conversion on planar and nanoparticulate anatase TiO_2_(101). **a**, **b** TPRS spectra of ketene (black), acetone (red), and recombinatively formed acetic acid (blue) collected under UHV conditions following acetic acid exposure at 100 K. **a** Planar TiO_2_(101) (2 ML dose). **b** Supported “thick” nanoparticulate TiO_2_(101) layer with 10 ML_eq_ (top) and 90 ML_eq_ (bottom) doses. **c** Integrated product yields from the “thick” layer as a function of acetic acid dose, showing linear increases in ketene and acetic acid, and a delayed rise in acetone. The temperature axis for nanoparticle spectra is shifted by 50 K relative to the planar crystal to account for differences in temperature measurements. **d** Batch reactor TPRS spectra for faceted TiO_2_(101) nanoparticles pressed onto a tungsten mesh. Increasing acetic acid doses (bottom to top) produce progressively more acetone, consistent with ketene-mediated C–C coupling.

We used temperature-programmed reaction spectroscopy (TPRS) to compare the catalytic reactivity of the nanoparticulate TiO_2_(101) layers to the planar TiO_2_(101). For the planar system, we previously showed that acetic acid deprotonates at low temperatures (~150 K), forming surface-bound acetate on adjacent Ti_5c_ sites and hydrogen on a bridging O_2c_ site^15,36^. Figure 2a shows the TPRS spectra for carbon-containing products from planar TiO_2_(101) following a saturation dose of acetic acid (1 CH_3_COOH per two Ti_5c_ sites). Consistent with previous studies^15^, only ketene (black) and recombinatively formed acetic acid (blue) are observed. The acetone signal (red), shown with tenfold amplification, remains flat, indicating no detectable acetone production. Both ketene and acetic acid desorb over the same temperature range (500–750 K), with a peak near 650 K and a weak shoulder around 750 K, suggesting the presence of two kinetically distinct ketene formation pathways. The CO_2_ and H_2_O products are shown in Supplementary Fig. 7, and a complete acetic acid coverage-dependent set of TPRS spectra is displayed in Supplementary Fig. 8. Since all hydroxyls formed upon acetic acid deprotonation recombine to desorb as water near 300 K^15^, the acetic acid observed between 550–800 K must result from reaction between surface acetates and hydroxyls generated by C–H bond cleavage. The nearly 1:1 ratio of ketene to acetic acid yields (quantified on planar TiO_2_(101))^15^ indicates that all hydrogen liberated during C–H bond scission is consumed in acetic acid recombination. This is further supported by the absence of water desorption in this temperature range (Supplementary Fig. 7). Notably, the constant ketene-to-acetic acid desorption ratio across the full 550–800 K range suggests that the probability of hydroxyl recombination with acetates is largely temperature-independent.

A similar set of TPRS spectra is observed for the thick layer of supported TiO_2_(101) nanoparticles when only the topmost layers are saturated with acetic acid (Fig. 2b, top). Dosing 10 ML-equivalents of acetic acid, sufficient to saturate approximately 2–3 layers of nanoparticles (see D₂O TPD discussion above), yields primarily ketene and recombined acetic acid, mirroring the behavior of the planar surface. A slight shift in product desorption temperatures likely reflects differences in temperature measurement between the planar crystal and nanoparticle-supported samples.

For the smaller acetic acid dose, the ketene desorption profile closely matches that from planar TiO_2_(101) and the acetone signal (Fig. 2b, top, red trace) remains largely flat. However, subtle differences appear in the desorption of recombinatively formed acetic acid. While the leading edge and peak position align with those of ketene, the high-temperature shoulder seen on the planar surface is absent. Because C–H bond cleavage during ketene formation generates surface hydroxyls, the absence of acetic acid desorption at higher temperatures implies that these hydroxyls are instead recombining to form water. This interpretation is supported by the appearance of a water peak in this temperature range (Supplementary Fig. 9 bottom and Supplementary Fig. 10). Although the origin of the difference in the acetic acid desorption profile compared to planar TiO_2_(101) is not established, we speculate that multiple collisions of recombinatively formed acetic acid molecules with TiO_2_(101) nanoparticles during desorption increase the probability of readsorption and deprotonation, thereby promoting additional ketene and water formation.

As the acetic acid dose increases on the thick sample, the ketene and recombinative acetic acid desorption peaks retain their characteristic line shapes, even at the highest dose of 90 ML_eq_ (Fig. 2b, bottom and Supplementary Fig. 9 top). In contrast to the lower-dose cases, the TPRS trace for acetone (red) now shows a distinct desorption feature emerging around 550 K and peaking at 670 K. This behavior resembles that observed under more realistic catalytic conditions. We propose that acetone formation at higher acetic acid doses, where deeper layers of the catalyst are saturated, results from multiple collisions of gas-phase ketene with acetate-covered TiO_2_(101) nanoparticles. As ketene migrates toward the outer surface, it encounters adsorbed acetate on neighboring particles, enabling acetone formation via C–C coupling. Although yields under UHV conditions are modest (~1%) due to limited residence time, shallow particle layer depth, and low collision probability, the systematic increase in acetone formation with dose and layer thickness demonstrates that ketene-mediated chemistry is accessible under the conditions studied. At higher pressures and in thicker catalyst beds, longer residence times and more frequent surface encounters are expected to increase the contribution of this pathway. However, its quantitative contribution under steady-state catalytic conditions will depend on the surface coverages and relative abundance of acetate, monodentate acetic acid, and enolate species.

While recombinatively formed acetic acid is also observed in this temperature range, it is unlikely to be the principal mobile intermediate leading to acetone. This assignment is based on the relative temperature profiles of the products. Recombinatively formed acetic acid is most prominent below ~600 K, where very little acetone is detected, and its yield decreases steadily at higher temperatures. Acetone desorption, in contrast, begins mainly above 600 K, when recombinatively formed acetic acid desorption is largely complete. Ketene exhibits a broader desorption profile, with a high-temperature shoulder that overlaps closely with the acetone desorption feature. Together with the nonlinear dose dependence of acetone production in Fig. 2c and the batch reactor trends in Fig. 2d (discussed below), these observations are most consistent with ketene, rather than recombinatively formed acetic acid, acting as the mobile gas-phase intermediate that reacts with acetate-covered TiO_2_(101) nanoparticles to form acetone.

Experiments analogous to those on the “thick” layer (Fig. 2b and Supplementary Fig. 10) were also performed on “medium” and “thin” layers (see Supplementary Figs. 11 and 12). Product evolution in these thinner films follows the same general trends (Supplementary Fig. 13). Based on D_2_O TPD analysis (Fig. 1d and Supplementary Fig. 5), the “thin” and “medium” layers correspond to approximately 2 and 4 layers of nanoparticles, respectively. As a result, ketene and acetone yields saturate after only a few ML-equivalents of acetic acid. Acetone formation is undetectable in the “thin” sample and remains minimal in the “medium” layer.

The evolution of product yields with increasing acetic acid dose for the “thick” layer is shown in Fig. 2c. Both ketene and recombinative acetic acid yields increase linearly with dose, indicating that the catalyst layer remains unsaturated even at the highest exposures (~100 ML_eq_). In contrast, the acetone yield displays a strongly nonlinear trend: it is negligible below ~20 ML_eq_, followed by a steady rise at higher doses. The full dataset is consistent with the acetone yield increasing with the increasing depth of the saturated nanoparticulate TiO_2_(101) bed. These results support the involvement of ketene as a reactive mobile intermediate en route to acetone under the low-pressure, temperature-programmed conditions studied here.

Isotopic labeling experiments were also attempted to further test the proposed ketene-mediated pathway. However, the use of CD_3_COOD leads to significant mass-spectral overlap among key products in our quadrupole mass spectrometer (see Supplementary Fig. 14). In particular, CD_2_CO has a parent ion at m/z = 44, overlapping with CO_2_, while CD_3_COCD_3_ and CD_3_COOD both give parent ions at m/z = 64. Mixed CH_3_COOH/CD_3_COOD dosing experiments (not shown) are also complicated by overlap among partially exchanged acetic acid isotopologues, including CH_2_DCOOH/CH_3_COOD at m/z = 61 and CD_2_HCOOD/CD_3_COOH at m/z = 63. These overlaps prevent an unambiguous isotope-tracing assignment under the present experimental conditions.

To further probe the competition between ketene and acetone formation, we investigated acetic acid conversion on faceted TiO_2_(101) nanoparticles pressed onto a tungsten mesh and studied in a batch reactor integrated with a TPRS setup. In each experiment, a pulse of acetic acid was introduced into the sample compartment, and the resulting pressure rise was recorded, with higher pressures corresponding to larger injected doses. After dosing, the sample was evacuated under high vacuum for 30 min to remove physisorbed and weakly bound acetic acid prior to TPRS analysis. These experiments are conceptually analogous to those in Fig. 2b: higher acetic acid doses, reflected by larger pressure increases, are expected to saturate progressively deeper layers within the nanoparticulate TiO_2_(101) bed.

The results of these batch reactor experiments are shown in Fig. 2d. In the lowest-dose case (bottom panel), where the pressure rise was below the detection limit of the capacitance manometer, the TPRS signal from ketene (black) exceeds that from acetone (red). The peak positions and line shapes closely match those observed in Fig. 2b. As the acetic acid dose increases (Fig. 2d, middle panel), both ketene and acetone signals grow, with acetone increasing more rapidly. At the highest dose (top panel), the acetone signal becomes dominant. This progression is consistent with a ketene-mediated contribution to acetone formation and supports the mechanistic picture developed from the UHV studies on nanoparticulate TiO_2_(101) layers. We note that these batch reactor TPRS experiments still probe transient conditions after evacuation rather than true steady-state catalytic operation, and therefore, they are best interpreted as evidence for the feasibility of ketene-mediated coupling rather than as a quantitative measure of its contribution under high-pressure reaction conditions.

### Ab initio mechanistic studies

To gain further insight, we investigated the role of ketene in acetone formation using DFT calculations on a periodic anatase TiO_2_(101) *p*(1 × 4) slab model (see “Methods” and Supplementary Fig. 15). The most stable adsorption configuration for acetic acid is a dissociated bidentate acetate species (Supplementary Fig. 16), consistent with experimental observations that this is the dominant surface species under UHV conditions^15^. At elevated temperatures, the bidentate acetate can recombine with a surface-bound hydrogen atom, forming molecular acetic acid (Supplementary Fig. 16), which then desorbs and may engage in subsequent surface reactions as discussed below.

**Fig. 3:**
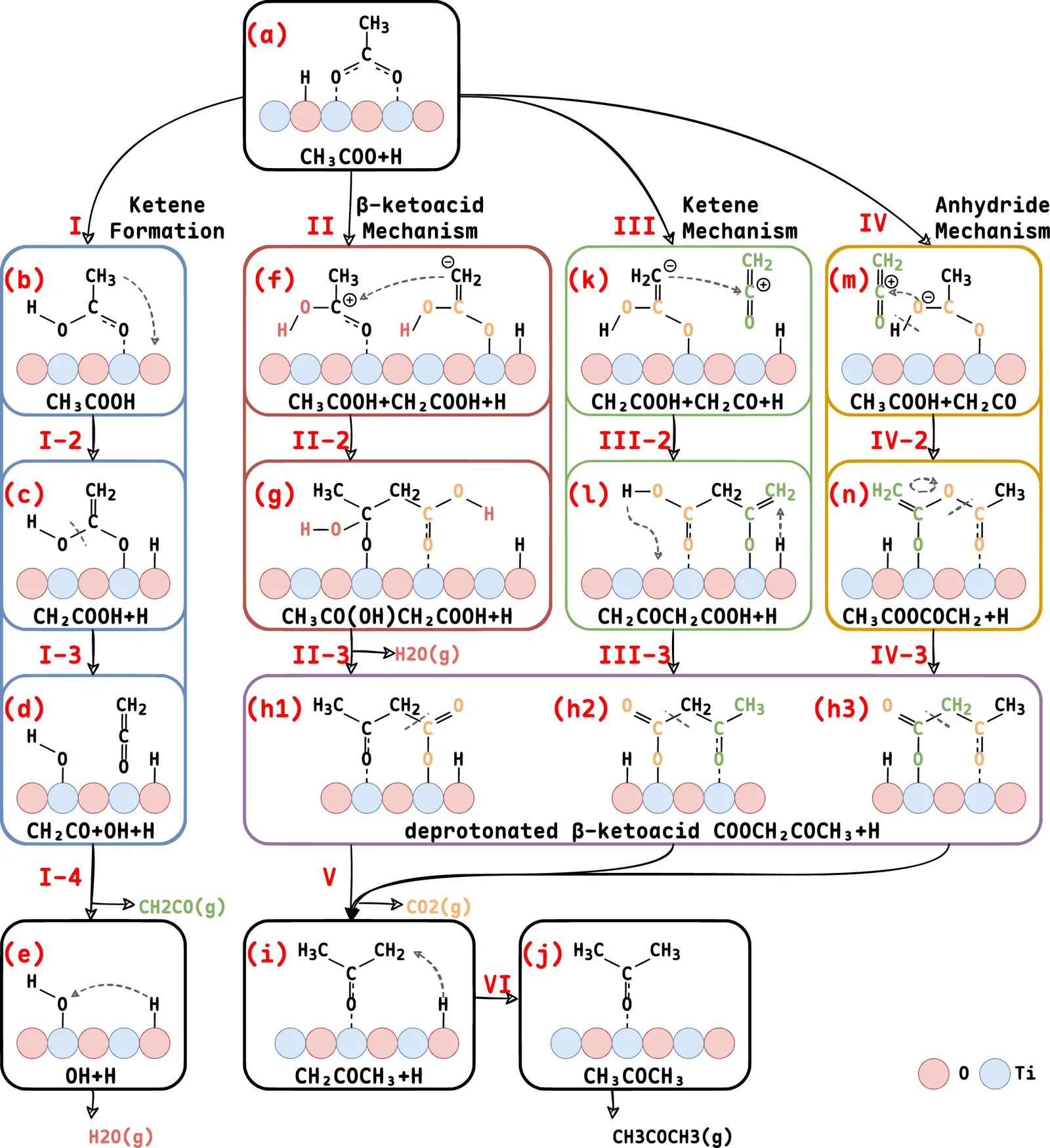
Proposed reaction network for ketene (CH_2_CO) and acetone (CH_3_COCH_3_) formation from acetic acid (CH_3_COOH) on anatase TiO_2_(101). All pathways originate from bidentate acetate (**a**). Pathway I describes ketene formation via enolization and subsequent decomposition through intermediates **b**–**e,** corresponding to the overall reaction CH_3_COOH → CH_2_CO + H_2_O. Acetone can form through three distinct mechanisms that converge to a common deprotonated β-ketoacid intermediate (**h1–h3**). In the β-ketoacid mechanism (Pathway II), a monodentate acetic acid species couples with a 1-hydroxy enolate through intermediates **f** and **g,** leading overall to 2CH_3_COOH → CH_3_COCH_3_ + CO_2_ + H_2_O. In the ketene mechanism (Pathway III), ketene reacts with a 1-hydroxy enolate through intermediates **k** and **l,** corresponding to the overall reaction CH_2_CO + CH_2_COOH + H → CH_3_COCH_3_ + CO_2_. In the anhydride mechanism (Pathway IV), ketene reacts with a monodentate acetic acid species to form an anhydride intermediate through intermediates **m**, **n** corresponding to CH_2_CO + CH_3_COOH → CH_3_COCH_3_ + CO_2_. The resulting β-ketoacid intermediates undergo decarboxylation (step V) to form an acetone enolate. **i** Followed by proton transfer (step VI) to form acetone. **j** The key nucleophilic attack steps are illustrated in structures **f**, **k**, and **m**.

The proposed reaction network is summarized in Fig. 3. Ketene formation (mechanism I) proceeds via conversion of bidentate acetate (structure a) to monodentate acetic acid (b), followed by enolization and decomposition of the resulting 1-hydroxy enolate. The net reaction is CH_3_COOH → CH_2_CO + H_2_O. On the other hand, acetone formation can occur via three mechanistic pathways. The widely accepted *β-ketoacid mechanism* (mechanism II) involves C–C coupling between a monodentate acetic acid and a 1-hydroxy enolate derived from acetate (step II-2)^10^, ultimately yielding acetone via the overall reaction 2CH_3_COOH → CH_3_COCH_3_ + CO_2_ + H_2_O. In the second route, termed the *ketene mechanism* (mechanism III), ketene couples with a surface-bound 1-hydroxy enolate (step III-2). The overall process is CH_2_CO + CH_2_COOH + H → CH_3_COCH_3_ + CO_2_. The third route, *anhydride mechanism* (mechanism IV), proceeds via the formation of a surface anhydride intermediate from the reaction of ketene with monodentate acetic acid (step IV-2), corresponding to CH_2_CO + CH_3_COOH → CH_3_COCH_3_ + CO_2_. Although these three mechanisms are often treated as parallel^8^, our calculations reveal that they all converge to a common deprotonated β-ketoacid intermediate (h1, h2, and h3).

**Fig. 4:**
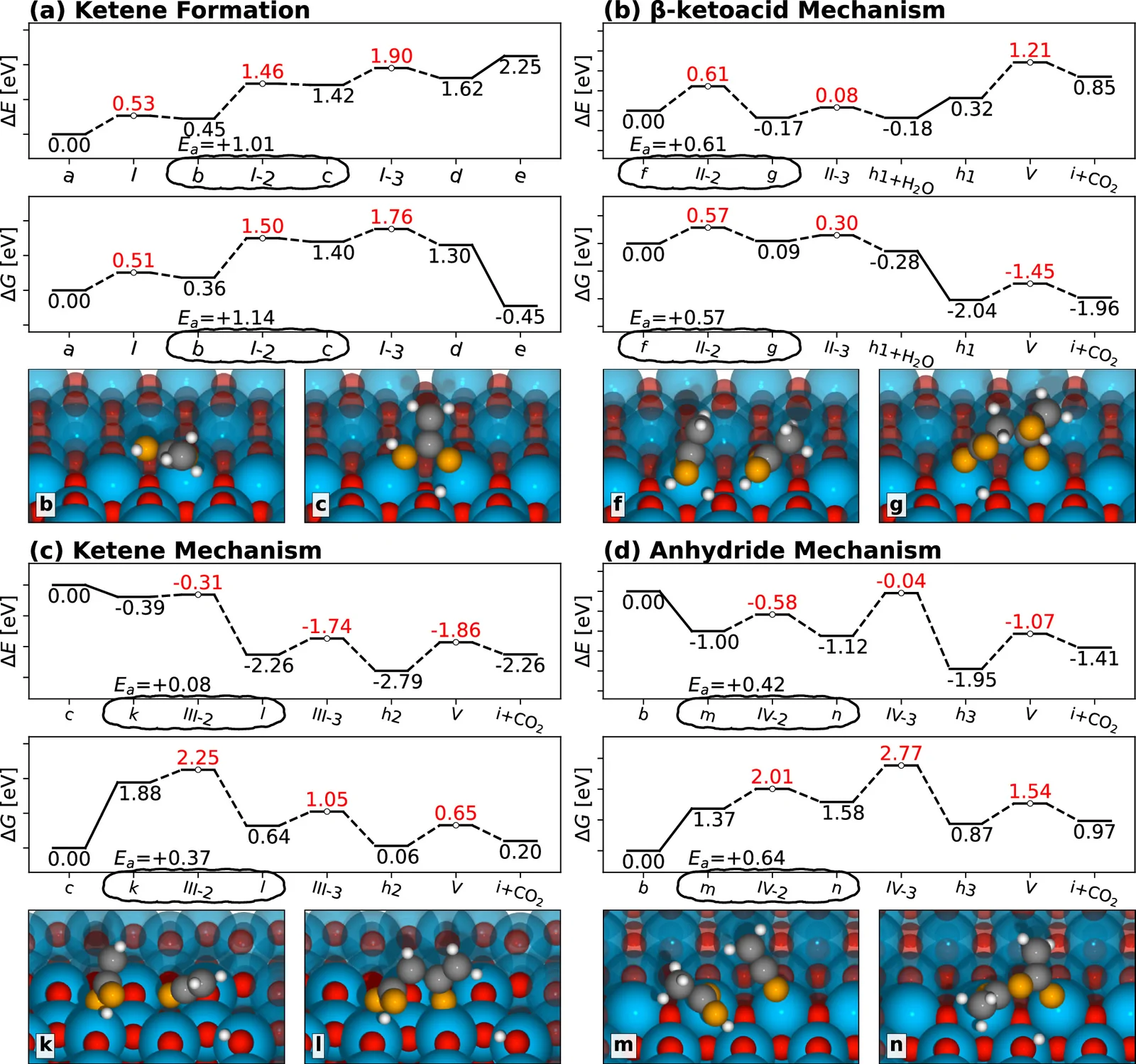
Total energy (Δ*E*) and free energy (Δ*G*) profiles for ketene and acetone formation on TiO_2_(101), calculated using DFT. Free energies were computed at 650 K and 10^−7^ Torr, corresponding to UHV experimental conditions. Letters and roman numerals along the reaction coordinate correspond to intermediates and transition steps in the reaction network (Fig. 3). Energies of intermediates (black) and transition states (red) are referenced to the initial structure of each pathway. Key reaction steps are circled with associated activation energies labeled, and their initial and final states are shown at the bottom of each panel. **a** Ketene formation via decomposition of enolized monodentate acetic acid. **b** β-Ketoacid mechanism: acetone formation via coupling of monodentate acetic acid with a 1-hydroxy enolate. **c** Ketene mechanism: coupling of ketene with a CH₂-containing 1-hydroxy enolate. **d** Anhydride mechanism: reaction of ketene with a monodentate acetic acid. Ti and O atoms in TiO_2_(101) are shown in cyan and red, respectively. C, H, and O atoms in adsorbates are shown in gray, ivory, and orange, respectively.

The total and free energy profiles for the key steps in ketene and acetone formation are highlighted in Fig. 4. Free energies were computed under conditions matching the experiment: 650 K and 10^−7^ Torr, where acetic acid conversion yields ~50% ketene and ~1% acetone. For comparison, profiles at 650 K and 0.007 Torr (relevant to higher-pressure experiments) are shown in Supplementary Fig. 17. Aside from the adsorption and desorption steps, the total and free energy profiles are nearly identical.

We first focus on the ketene formation pathways. In the most favorable mechanism (Fig. 4a), the rate-determining step is the enolization of monodentate acetic acid, which originates from the recombination of bidentate acetate with a surface hydroxyl (H*) at a bridging O_2c_ site. This transformation requires a sequence of structural rearrangements with an effective energy barrier of +0.69 eV (Supplementary Fig. 16). The resulting monodentate acetic acid, stabilized by a single O–Ti_5c_ bond, is conformationally flexible and undergoes H transfer from the methyl group to a nearby O_2c_ site, forming a short-lived 1-hydroxy enolate (CH_2_COOH). The enolization step has an energy barrier of +1.01 eV (= 1.46–0.45 eV), and the negligible reverse barrier suggests the equilibrium lies heavily toward the acetic acid. It should be noted that enol formation represents the highest energy barrier shared across all reaction pathways to acetone (Fig. 4b–d).

This short-lived 1-hydroxy enolate has been predicted on other metal oxides, including ZrO_2_^37^ and Cu_2_O^16^. Its decomposition via homolytic C–OH bond cleavage (Fig. 4a) proceeds with a barrier of +0.48 eV (= 1.90–1.42 eV), yielding nascent ketene. The latter tends to weakly physisorb near a Ti_5c_ site, stabilized by interaction between its CH_2_ group and H*, at low temperature. However, under reaction conditions, ketene desorption is thermodynamically favored, consistent with the large ketene signal observed in TPD (Fig. 2a). Indeed, conversion of this physisorbed state to a more stable chemisorbed configuration carries a barrier of ~0.52 eV (Supplementary Fig. 18), indicating that desorption outcompetes further adsorption. This behavior increases the likelihood that ketene will participate in secondary surface reactions on nearby nanoparticles. Finally, the recombination of H* and OH* to form water has a low barrier of +0.24 eV.

An alternative ketene formation route involves the direct molecular decomposition of acetic acid, analogous to the known pathway for formic acid decomposition^38,39^, and may become relevant at high surface coverages on faceted nanoparticles (Supplementary Fig. 19). In this mechanism, a molecular acetic acid adsorbed atop a layer of bidentate acetates undergoes simultaneous C–OH and C–H bond cleavage, yielding ketene, OH*, and H*. The rate-determining step in this pathway has a total energy barrier of +1.42 eV, which is significantly higher than the +1.01 eV barrier associated with enolization of monodentate acetic acid. In addition, this pathway requires a near-monolayer of molecular acetic acid. Therefore, enolization remains the dominant ketene formation route under typical conditions. However, under high coverage, where recombinatively formed acetic acid can adsorb atop neighboring nanoparticles, this molecular decomposition channel may contribute to the observed broad desorption feature for ketene, which spans a wider temperature range than that of acetic acid (Fig. 2a).

A recent study demonstrated that ketene can also arise from an acylium cation intermediate in zeolites^40^. In our system, we identified an analogous pathway proceeding via an adsorbed acylium species on TiO_2_(101) (Supplementary Fig. 20). However, formation of this acylium intermediate from molecular acetic acid requires a high barrier of +1.08 eV, and its subsequent α-H abstraction step carries an even higher barrier of +1.30 eV. Consequently, although mechanistically conceivable, the acylium route is kinetically disfavored under typical reaction conditions compared to the two pathways discussed above and is unlikely to contribute appreciably to ketene formation.

For acetone formation, three mechanistic pathways are possible, all converging on a deprotonated β-ketoacid intermediate that subsequently decarboxylates to yield an acetone enolate. Although the three pathways lead to the same chemical intermediate, the resulting surface species (h1, h2, and h3) adopt distinct adsorption geometries inherited from the preceding C–C coupling and proton-transfer steps. As shown in Supplementary Fig. 21, these geometric differences give rise to slightly different transition-state structures for the subsequent decarboxylation step, resulting in modest variations in the calculated activation barriers (+0.89, +0.93, and +0.88 eV for h1, h2, and h3, respectively).

In the first mechanism, the widely accepted β-ketoacid pathway, a monodentate acetic acid couples with a neighboring 1-hydroxy enolate derived from a second monodentate acetic acid (mechanism II in Figs. 3 and 4b). Once the enolate forms at a Ti_5c_ site adjacent to the monodentate acetic acid, its CH_2_ group can attack the carbonyl carbon to yield a hydroxylated β-ketoacid (structure g). This intermediate then dehydrates, either intra- or intermolecularly (Supplementary Fig. 22), to form the deprotonated β-ketoacid (h1). Decarboxylation of this β-ketoacid yields an acetone enolate, whose CH_2_ group readily captures a surface proton (H*) to form acetone, with an energy barrier of only +0.18 eV (Supplementary Fig. 23). We note that in this pathway, the carbonyl carbon in the acetone originates from the monodentate acetic acid, while the carbon lost as CO_2_ derives from the enolized acetic acid, consistent with isotope-labeling experiments using pivalic and ^13^C-labeled acetic acids, where ^13^C is found exclusively in the CO₂ product^19^.

Given the substantial production of ketene and its weak adsorption energy, it likely escapes into the gas phase and scatters through interparticle voids within the nanoparticulate TiO_2_(101) layer, where it can encounter reactive surface species capable of further reaction to form acetone. One such route involves ketene coupling with a 1-hydroxy enolate (mechanism III in Figs. 3 and 4c). In this mechanism, the carbonyl carbon of ketene couples with the CH_2_ group of the enolate via a low-energy barrier of +0.08 eV (structure k), yielding an α-enolate acetic acid intermediate (l). Subsequent proton transfer steps, H* addition to the CH_2_ group and migration of the OH proton to a bridging O_2c_ site, enable rearrangement to a deprotonated β-ketoacid (h2). This transformation involves an effective energy barrier of +0.52 eV, attributed to H* hopping between adjacent sites. An alternative pathway involves ketene coupling with a carboxylate formed by enolization of a bidentate acetate (Supplementary Fig. 24), with a higher coupling barrier of +0.34 eV than the 0.08 eV barrier for ketene with the 1-hydroxy enolate. Both pathways converge on the same deprotonated β-ketoacid intermediate, which decarboxylates to form an acetone enolate and, ultimately, acetone. Unlike in the β-ketoacid pathway, in these ketene-mediated mechanisms, the carbonyl carbon of acetone originates from ketene, while the CO_2_ carbon derives from the enolized acetic acid. This isotopic distribution appears inconsistent with the pivalic acid labeling experiment^19^, in which ^13^C is found exclusively in CO_2_. However, these mechanisms rely on the presence of an enolate coupling partner. In the pivalic acid experiment, the absence of α-H in pivalic acid precludes enolate formation, thus preventing cross-ketonization via ketene pathways. Therefore, the pivalic acid isotopic labeling experiments do not rule out this mechanism as a possible contributor to ketonization.

The final investigated mechanism (mechanism IV in Figs. 3 and 4d) involves the reaction of ketene with a monodentate acetic acid (structure m). Rather than direct C–C coupling, the hydroxyl group of the acetic acid attacks the carbonyl carbon of ketene, simultaneously transferring its proton to a neighboring O_2c_ site, forming an anhydride intermediate (n). Under conditions where both ketene and acetic acid are abundant, this pathway becomes favorable: the anhydride forms readily due to a low forward barrier of +0.42 eV and persists because of a relatively high reverse barrier of +0.54 eV. The anhydride can then undergo an intramolecular rearrangement to generate a deprotonated β-ketoacid with a barrier of +1.08 eV. In this pathway, the carbonyl carbon of the acetone originates from the acetic acid, while the CO₂ carbon is derived from the ketene. This is consistent with the pivalic acid labeling experiment, where all ^13^C was found in the CO_2_, supporting the viability of this mechanism for cross-ketonization, as long as the steric hindrance from the bulky tert-butyl group in pivalic acid does not preclude anhydride formation.

## Discussion

Our experiments demonstrate that gas-phase ketene is a viable intermediate in the ketonization of acetic acid on TiO_2_ catalysts. Using nanoparticulate TiO_2_(101) layers of varying thickness, we show that ketene, formed via enolization and decomposition of surface-bound acetate, can desorb, migrate through interparticle voids, and undergo gas-phase collisions with neighboring surface species to form acetone. Surface science and batch reactor experiments reveal a clear correlation between ketene and acetone yields as the nanoparticulate TiO_2_(101) layers become saturated to increasing depths from the external surface. DFT calculations support this interpretation, showing that ketene reacts readily with surface enolates or monodentate acetic acid via low-barrier pathways converging on a common β-ketoacid intermediate.

The approach developed in this work enables direct investigation of reactive intermediates engaging in spatially decoupled elementary steps within the catalyst bed and can be extended to other catalytic systems involving mobile intermediates. In the case of acetic acid ketonization, it reveals that ketene is not a nonparticipating by-product, but can serve as a mobile intermediate that drives C–C coupling. These findings establish a conceptual framework in which gas-phase transport of volatile intermediates expands the effective catalytic landscape, enables spatially decoupled reactivity, and offers new strategies for tuning reactivity and selectivity in complex catalytic environments.

## Methods

Extensive experimental and computational details, including sample synthesis, characterization, dosing procedures, and additional spectra, are provided in the Supplementary Information. Below, we summarize the essential aspects relevant to the main text.

### Catalyst preparation

The nanoparticulate TiO_2_(101) with well-defined morphology was hydrothermally synthesized via a modified two-step approach^22,24^. First, potassium titanate precursor was synthesized as follows: 22.4 g of KOH (Sigma-Aldrich, ≥ 85 wt%) was dissolved in 40 mL of deionized water, followed by adding 1 g of P25 (Aldrich, nanopowder, 21 nm in diameter, ≥ 99.5 wt%) while stirring. The obtained suspension was transferred into a Teflon-lined stainless-steel autoclave, which was kept at 200 °C for 24 h. After synthesis, the white precipitate was treated by five-times repeated washing-centrifugation and subsequent drying at 80 °C overnight. TiO_2_(101) was prepared by adding 0.2 g of the precursor to 250 mL of deionized water and sonicating for 45 min. The obtained solution was sealed in a Teflon-lined stainless-steel autoclave, and hydrothermal synthesis was conducted at 200 °C for 24 h. The as-synthesized catalysts were washed five times using deionized water, dried in air at 80 °C overnight and calcined in the muffle furnace at 550 °C for 4 h.

### Catalyst characterization

Catalyst morphology and structure were characterized using a combination of electron and spectroscopic techniques (see Supplementary Figs. 1–3). Scanning electron microscopy (SEM) and helium ion microscopy (HeIM) were used to visualize particle morphology and size. Surface chemical composition and oxidation states were analyzed using X-ray photoelectron spectroscopy. Catalyst structure and crystallinity were assessed using X-ray diffraction and Raman spectroscopy.

### TPRS measurements

Temperature-programmed desorption and reaction spectroscopy (TPD/TPRS) experiments were conducted in an ultra-high vacuum (UHV) system equipped with a molecular beam source for precise dosing and a quadrupole mass spectrometer for product detection. Single crystal TiO_2_(101) and supported nanoparticulate TiO_2_(101) layers were mounted on tantalum plates, resistively heated, and temperature-calibrated against the desorption of reference adsorbates (Kr or D_2_O). Adsorbates such as D_2_O and CH_3_COOH were introduced onto the surfaces at controlled temperatures, with coverages referenced to the density of Ti_5c_ sites on TiO_2_(101). Clean surfaces were prepared by sputtering and O_2_ annealing (single crystal) or high-temperature degassing and O_2_ annealing (nanoparticles), and TPRS spectra were recorded during linear heating at 1 K/s with corrections for product fragmentation.

For nanoparticle-layer experiments, faceted TiO_2_(101) nanoparticles were deposited onto optically polished Au-coated Ta substrates by spray-casting or drop-casting suspensions of the nanocrystals. The resulting films were mounted in the same UHV TPD/TPRS apparatus used for the planar TiO_2_(101) single crystal. The samples were resistively heated through the Ta support, and the temperature was monitored using a K-type thermocouple attached to the back of the Ta plate. The nanoparticle temperature calibration was checked using the D_2_O desorption peak relative to planar TiO_2_(101). Prior to dosing, the nanoparticle films were heated to 900 K to remove adsorbed species and annealed in an O_2_ beam; cleanliness was confirmed by the absence of desorption products in a blank TPD experiment. D_2_O and CH_3_COOH were dosed with a molecular beam at controlled sample temperatures, and TPD/TPRS spectra were recorded during linear heating at 1 K/s. Coverages on nanoparticle samples are reported as ML-equivalents referenced to the Ti_5c_ site density of planar TiO_2_(101); therefore, 1 ML-equivalent does not correspond to saturation of all active sites within the nanoparticle bed.

### Ab initio calculation

All DFT calculations were performed using the Perdew-Burke-Ernzerhof (PBE) exchange-correlation functional^41^ with Grimme’s D3 dispersion corrections^42^, as implemented in the CP2K package^43^. The Goedecker-Teter-Hutter (GTH)^44^ norm-conserving pseudopotentials and a hybrid Gaussian-planewave basis set with a cutoff energy of 800 Ry were used to describe the core electrons.

The catalyst surface was modeled using a p(1 × 4)-TiO_2_ slab (10.32 × 15.16 Å) with four Ti layers as shown in Supplementary Fig. 15. A vacuum region ∼15 Å wide was added along the z direction to avoid spurious interactions between periodic images. The dipole correction was used to remove possible electric fields in the vacuum region. The atoms in the bottom half-slab were fixed in all the calculations. Structural optimizations were continued until the residual forces on unfixed atoms were less than ∼0.02 eV/Å. Reaction pathways and barriers were determined via the Climbing Image Nudged Elastic Band (CINEB) method^45^. The transition state, which is the highest energy point along the minimum-energy pathway, was refined to a force tolerance of ∼0.05 eV/Å and verified by vibrational frequencies.

The Gibbs free energies were obtained through a correction on the total energies based on vibrational frequencies using the Atomic Simulation Environment (ASE)^46^ (details in the Supplementary Information). The vibrational analysis only considers adsorbates and uses a finer grid on the exchange-correlation functional to improve the accuracy of the vibrational frequencies.

## Supplementary information

Supplementary Information

Transparent Peer Review file

## Data Availability

The data generated in this study, including the source data underlying Figs. 1–4 and Supplementary Figs. 1–24 and the computational structures and input files have been deposited in the Zenodo repository at https://doi.org/10.5281/zenodo.21708272. ^47^
