# Supplementary material for: Ketene as a gas phase intermediate in acetic acid ketonization on TiO2
Source: Nat Commun. 2026 Oct 28;17:11209. doi: 10.1038/s41467-026-77945-8 (PMC13720007; doi:10.1038/s41467-026-77945-8)
Supplement: Supplementary file 1 [file 41467_2026_77945_MOESM1_ESM.pdf]

Ketene as a Gas Phase Intermediate in Acetic Acid Ketonization on TiO_2

Jiayan Xu[1]†, William JI DeBenedetti[2]†‡, Xingyu Wang[2]†§, Yiqing Wu[2], Libor Kovarik[2], Christopher R. O'Connor[2], Feng Gao[2], Janos Szanyi[2], Yong Wang[2,3], Annabella Selloni[1]*, Greg A. Kimmel[2]*, Zdenek Dohnálek[2,3]*

[1] Department of Chemistry, Princeton University, Princeton, NJ 08544, USA.

[2] Physical and Computational Sciences Directorate and Institute for Integrated Catalysis, Pacific Northwest National Laboratory, Richland, WA 99354, USA.

[3] Voiland School of Chemical Engineering and Bioengineering, Washington State University, Pullman, Washington 99163, USA.

*Corresponding authors: A.S.: aselloni@princeton.edu, G.A.K.: gregory.kimmel@pnnl.gov, Z.D.: zdenek.dohnalek@pnnl.gov.

† These authors contributed equally to this work

Current address:

‡ Plutonium Supply & Disposition (PSD-4) Los Alamos National Laboratory, Los Alamos, New Mexico 87545, USA

§ Commonwealth Fusion Systems, Devens, MA 01434, USA

Supplementary Methods

Scanning Electron Microscopy (SEM) and He-Ion Microscopy

SEM was performed on a Helios 600 Nano Lab. Samples were coated with a thin layer of carbon to improve conductivity. SEM imaging was performed using beam voltages of 2-3 kV and 50-100 pA of current using through-lens detection.

He-Ion Microscopy (HeIM)

HeIM images were acquired using a Zeiss Orion HeIM. The microscope was operated at optical imaging voltages of 20-30 kV using an aperture size of 60 μm with a beam blanking current of 0.6 pA. An Everhart–Thornley detector was used throughout. A variety of magnifications and tilts were used to obtain high-resolution images that captured particle morphology and size.

Scanning transmission electron microscopy (STEM)

STEM analysis was performed with an aberration-corrected Thermo Fisher Themis Z scanning transmission electron microscope. The observations were performed at 300 kV in scanning mode using a high-angle annular dark-field (HAADF) detector. The probe convergence angle was 25 mrad, and the inner detection angle on the HAADF detector of 52 mrad. The presented image was collected using multi-frame acquisition, mostly consisting of 50 images. The acquisition and basic image processing were performed with Thermo-Fisher's Velox software.

X-Ray Photoemission Spectroscopy (XPS)

XPS was performed using unmonochromated Mg Kα irradiation on an Omicron XPS equipped with a 7-channel detector. Photoelectrons were collected at 70° from the surface normal. A Tougaard or Shirley baseline was removed from all reported spectra.[1] The spectra were normalized using the area of the Ti 2$p_{3/2}$. Small energy corrections were applied to reported spectra using reference energies to offset mild band bending.

X-Ray Diffraction (XRD)

X-Ray Diffraction (XRD) was performed on a Rigaku Smart Lab using as-synthesized nanocrystals. Each transition was indexed to known reflections reported on crystalline anatase. Gaussian Fitting was performed on each transition to extract the peak position and linewidth. Scherrer analysis was used to calculate the average particle size from the diffraction pattern.[2]

Raman Spectroscopy

Raman spectroscopy was performed using a Horiba LabRAM HR Evol Raman spectrometer with a 633 nm excitation laser and a thermoelectrically cooled CCD. Presented spectra are an average of 5 accumulations using a 1 s integration from 0 – 1000 cm^{-1}.

Sample preparation for SEM, He-Ion microscopy, XPS, and H_2O adsorption and Acetic acid UHV Reactivity Studies

Temperature programmed desorption (TPD) experiments were performed in an ultra-high vacuum (UHV) system. The system is equipped with a closed-cycle helium cryostat for sample cooling, a molecular beam source for dosing adsorbates, a quadrupole mass spectrometer (Hiden, 3F-PIC), a Fourier-transform infrared spectrometer (Bruker, Model Vertex 80), a low-energy electron gun (Kimball Physics, Model ELG-2), Auger electron spectroscopy (Perkin-Elmer, Phi 15-155 Cylindrical Auger Electron Optics), and a low-energy electron diffraction spectrometer (Perkin-Elmer, Phi 15-120 LEED Optics). A naturally grown anatase $TiO_2(101)$ single crystal (SurfaceNet, $5 \times 5 \times 1$ mm^3, miscut angle $< 0.3^{\circ}$) was mounted on a tantalum plate using high-temperature conducting cement (Aremco 865), and a tantalum retaining plate covering the edges of the sample was used to secure the sample. The sample was resistively heated, and the temperature was measured with a K-type thermocouple spot-welded to the back of the tantalum mounting plate. The temperature of the surface is calibrated by comparing the Krypton desorption temperature read from the thermocouple to that of the known value of Krypton desorption temperature on anatase $TiO_2(101)$. The sample was cleaned by repeated cycles of Ne^+ sputtering (2 kV) followed by annealing under an O_2 beam (4 min, 720 K, flux $\sim5\times10^{13}$ molecules $cm^{-2}s^{-1}$) and vacuum annealing (5 min, 950 K).[3] The sample preparation yielded a reproducible desorption spectrum for 2 ML of adsorbed D_2O.[4] Water (D_2O) and acetic acid (CH_3COOH) were cleaned by 3 cycles of freeze-pump-thaw and dosed using a molecular beam at specified surface temperatures. The adsorbate coverages are reported in monolayers (MLs) defined relative to the density of Ti_{5c} sites (1 ML is 5.2×10^{14} molecules cm^{-2}) on a TiO_2 anatase single crystal. Temperature Programmed Reaction Spectroscopy (TPRS) was performed by adsorbing CH_3COOH, and the spectra were acquired using a heating rate of 1 K/s. The TPRS spectra were corrected for product mass fragmentation before quantification.

TiO_2 nanoparticles were deposited on an optically polished gold-coated tantalum plate by spray-casting of morphologically controlled anatase TiO_2 nanocrystals onto the substrate. Images shown in

the main article were representative of thick films used in temperature-programmed desorption measurements and were the result of 75 sprays of a 1g/40mL dispersion. The solvent was allowed to evaporate and was not heated or annealed. The medium nanoparticulate $TiO_2(101)$ layer was deposited by drop-casting a suspension on the surface of an Au-coated Ta plate; the suspension was made by mixing 5 mg of nanoparticle powder with 20ml ethanol and sonicating for 30 minutes. The suspension remained sonicated throughout the drop-casting procedure. Each drop was 20 μL; the ethanol film was dried before the next drop was cast. To achieve the desired thickness of nanoparticles on the medium sample, a total of 700 μL suspension was used. The distribution of nanoparticles was then examined by SEM to ensure even coverage across the sample. The TPD experiments were performed in the same UHV system as the single crystal described above. The samples were heated by spotwelding the back of the tantalum plate to a round tantalum wire and resistively heated through the tantalum wire. The temperature was measured with a K-type thermocouple spot welded to the back of the tantalum plate. The temperature of the nanoparticles was calibrated by comparing the D_2O desorption peak to that of a TiO_2 anatase single crystal. The desorption temperature of D_2O does not change with higher exposure on nanoparticles, indicating the temperature is consistent within the nanoparticulate $TiO_2(101)$ beds. Before a dosing experiment, the nanoparticulate $TiO_2(101)$ bed was heated to 900K to degas of any adsorbed molecules, then annealed under an O_2 beam (4 mins, 720K, flux $\sim 5\times10^{13}$ molecules $cm^{-2}s^{-1}$). The cleanliness was checked by running a TPD to 900 K and no desorption was observed in the spectra. Water (D_2O) and acetic acid (CH_3COOH) were dosed using a molecular beam at specified surface temperatures. The coverages of adsorbates are reported in monolayers (MLs) defined relative to the density of Ti_{5c} sites (1ML is 5.2×10^{14} molecules cm^{-2}) on TiO_2 anatase single crystal, not to the area density of nanoparticulate $TiO_2(101)$ layers. In other words, 1 ML of adsorbates does not cover all active Ti_{5c} sites on the nanoparticles. The estimation of coverage on the nanoparticles is discussed below. Temperature Programmed Reaction Spectroscopy (TPRS) was performed by adsorbing CH_3COOH, and the spectra were acquired using a heating rate of 1K/s.

Ab Initio Thermodynamics

Adsorption Energy. The adsorption energy E_{ads} is defined as

$$E_{ads} = E_{surface+molecule} - E_{surface} - E_{molecule(g)} \tag{1}$$

where $E_{surface+molecule}$ is the total energy of the surface with the adsorbed molecule, $E_{surface}$ is the total energy of the clean anatase TiO_2(101) surface, and $E_{molecule(g)}$ is the total energy of a gas phase molecule in its most stable configuration. The adsorption is stronger if E_{ads} is more negative.

Effective Barrier. The reaction barrier of an elementary step $\boldsymbol{E_a}$ is defined as

$$E_a = E_{TS} - E_{intermediate} \tag{2}$$

where $E_{intermediate}$ is the total energy of an intermediate and E_{TS} is the total energy of the transition state. E_a is the forward barrier if $E_{intermediate}$ is the initial-state energy E_{IS} while it is the reverse barrier if $E_{intermediate}$ is the final-state energy E_{FS}.

To further compare different reaction pathways, we define the effective barrier E_a^{eff} as the energy difference between the highest transition state and the initial state, which is given by

$$E_a^{eff} = \max_i E_{TS,i} - E_{IS} \tag{3}$$

where E_{TS}^i is the energy of the ith transition state along a given pathway. For example, for a pathway containing three transition states E_{TS}^1, E_{TS}^2, and E_{TS}^3 between two intermediates E_A and E_B of interest. The effective barrier is $\max(E_{TS}^i) - E_A$.

Free Energy Correction. The Gibbs free energies were obtained through a correction on the total energies using the Atomic Simulation Environment (ASE).[5]

For molecules in the gas phase, the Gibbs free energy can be computed in the ideal-gas limit using

$$\mathrm{G}(T,p) = E_{tot} + E_{ZPE} + \int_0^T C_p \mathrm{d}T - T * S(T,p) \tag{4}$$

where E_{tot} is the total energy, E_{ZPE} is the zero-point energy, and C_p is the constant-pressure heat capacity. The constant-pressure heat capacity can be obtained from the constant-volume heat capacity using

$$C_p = k_B + C_{V,elec} + C_{V,trans} + C_{V,rot} + C_{V,vib} \tag{5}$$

where $C_{V,elec}$ is the electronic heat capacity, $C_{V,trans}$ is the translational heat capacity, $C_{V,rot}$ is the

rotational heat capacity, $C_{V,vib}$ is the vibrational heat capacity. The C_V terms are given by

$$\begin{gathered} C_{V,elec} = 0 \\ C_{V,trans} = \frac{3}{2} k_B \\ C_{V,rot} = k_B \text{ if linear} \\ C_{V,rot} = \frac{3}{2} k_B \text{ if nonlinear} \\ \int_0^T C_{V,vib} dT = \sum_i^{DOF} \frac{\epsilon_i}{e^{\frac{\epsilon_i}{k_B T}} - 1} \end{gathered} \quad (6)$$

The entropy $S(T, p)$ is given by

$$S(T, p) = S_{elec} + S_{trans} + S_{rot} + S_{vib} - k_B \ln \frac{P}{P^\circ} \quad (7)$$

where S_{elec} is the electronic entropy, S_{trans} is the translational entropy, S_{rot} is the rotational entropy, S_{vib} is the vibrational entropy, and k_B is the Boltzmann constant. The entropy terms are given by

$$\begin{gathered} S_{elec} = k_B \ln[2 * N_{spin} + 1] \\ S_{trans} = k_B \left(\ln \left[\left(\frac{2\pi M k_B T}{h^2} \right)^{\frac{3}{2}} \frac{k_B T}{P^\circ} \right] + \frac{5}{2} \right) \\ S_{rot} = k_B \left[\ln \left(\frac{8\pi^2 I k_B T}{\sigma h^2} \right) + 1 \right] \text{ if linear} \\ S_{rot} = k_B \left[\ln \left(\frac{\sqrt{\pi I_A I_B I_C}}{\sigma} \left(\frac{8\pi^2 k_B T}{h^2} \right)^{\frac{3}{2}} \right) + \frac{3}{2} \right] \text{ if nonlinear} \\ S_{vib} = k_B \sum_i^{DOF} \left[\frac{\epsilon_i}{k_B T \left(e^{\frac{\epsilon_i}{k_B T}} - 1 \right)} - \ln \left(1 - e^{-\frac{\epsilon_i}{k_B T}} \right) \right] \end{gathered} \quad (8)$$

where k_B is the Boltzmann constant, N_{spin} is the spin multiplicity, M is the mass of the molecule, h is the Planck constant, I are the degenerated moment of inertia for a linear molecular and I_A, I_B, and I_C are three principle moments of inertia for a nonlinear molecule.

For adsorbates, the Gibbs free energy can be computed in the harmonic approximation using

$$G(T, p) = E_{tot} + E_{ZPE} + E_{vib} + pV - T * S_{vib} \quad (9)$$

where E_{tot} is the total energy, E_{ZPE} is the zero-point energy, E_{vib} is the vibrational energy, and S_{vib} is the vibrational entropy. The vibrational energy E_{vib} is given by

$$E_{vib} = \sum_i^{DOF} \frac{\epsilon_i}{e^{\frac{\epsilon_i}{k_B T}} - 1} \quad (10)$$

where DOF is the number of vibrational degrees of freedom, ϵ_i is the vibrational energy, k_B is the Boltzmann constant. In the vibrational analysis, we only consider the vibrational frequencies of the adsorbate. Because the adsorbates do not have real translational or rotational degrees of freedom, all

degrees of freedom of the adsorbate are taken as harmonic. The vibrational entropy S_{vib} is calculated using the same formula as that for the limit of ideal gas. Moreover, the pV term is assumed to be zero as it is negligible for adsorbates.

Supplementary Note 1. Surface area of the nanoparticles exposed to the molecular beam

To correlate the surface areas of individual nanoparticles with those of the planar surfaces, we evaluate the ratio between their total area and the area they project on a planar surface. Similarly, the ratios between their external surface area (visible to the molecular beam) and the area they project can also be determined. These ratios depend on the spatial orientation of the particle relative to the macroscopic surface normal.

The highest ratio of the nanoparticle surface area to the projected area of 5.4 is achieved when the nanoparticles are aligned with their long crystallographic axis perpendicular to the macroscopic surface normal (i.e., with the tetragonal pyramids pointing upwards). Further, for the nanoparticles oriented in this fashion, the surface area visible to the incoming molecular beam is 2.7 times that of a planar surface.

When the nanoparticles are stacked flat with the diagonal of their square base aligned along the surface normal, the total area is 3.0 times that of the projected planar surface, with a line-of-sight area 1.5 times that of the planar surface. Finally, if the nanoparticles are lying flat with one side of their square base oriented along the surface normal, the total area reaches 4.3 times that of a planar surface, with a line-of-sight area of 1.1 times.

Since the nanoparticles are randomly oriented, we pick the middle value of 4.3 times the planar surface for all the estimates used below. Based on this value, we determine that 90 ML-equivalents would saturate approximately 21 topmost layers of particles within the “thick” layer of supported nanoparticles. For the “medium” and “thin” layers, where we reached saturation by water, we estimate they consist of approximately 4 and 2 layers of particles, respectively.

Supplementary Figures

Supplementary Figure 1. X-Ray diffraction characterization of nanoparticulate TiO_2(101)

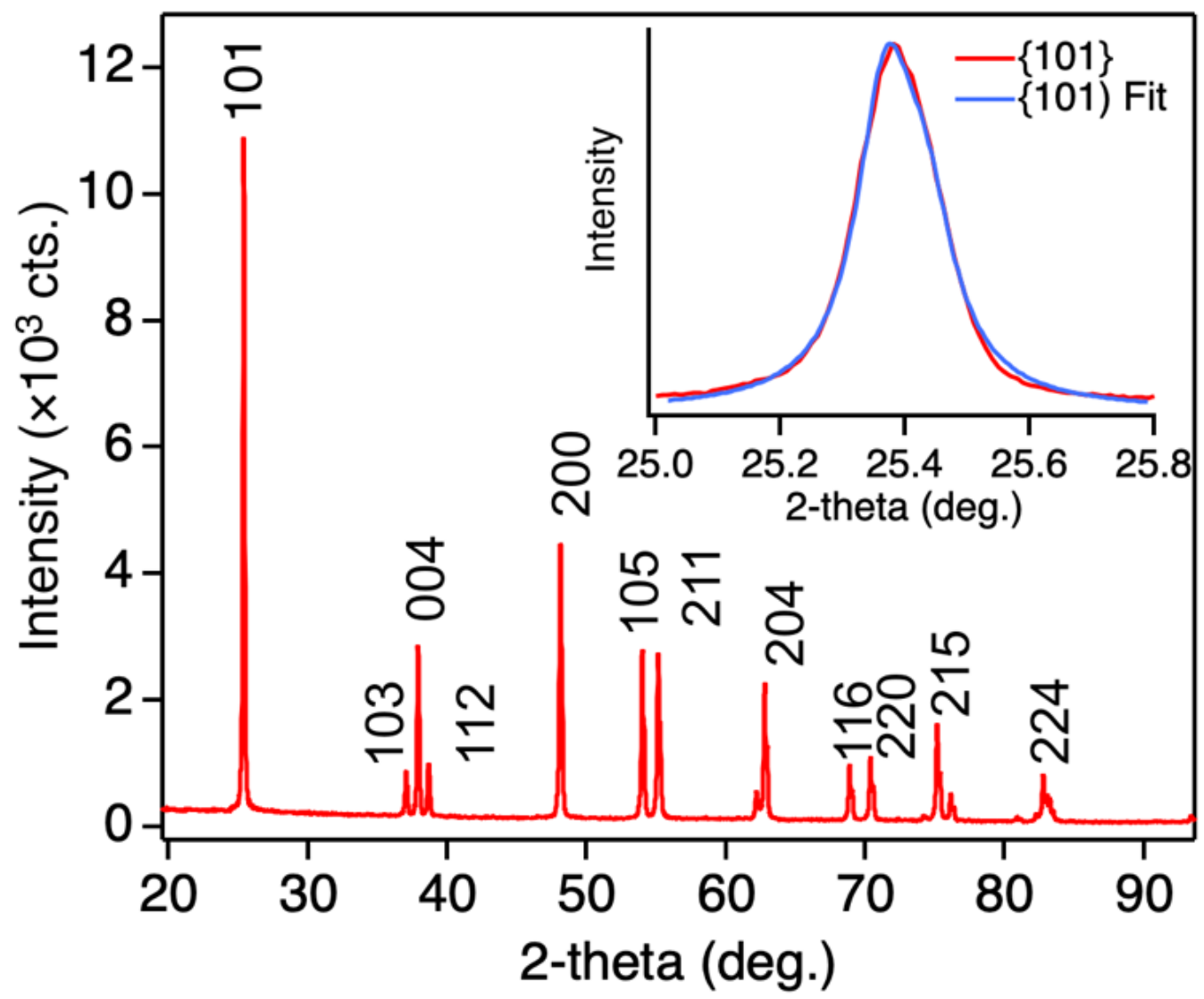

h	*k*	*l*	2θ (°)	FWHM	Crystal Size (nm)
1	0	1	25.37	0.057	25.1
1	0	3	37.02	0.062	20.9
0	0	4	37.88	0.056	25.9
1	1	2	38.63	0.066	20.3
2	0	0	48.09	0.062	22.3
1	0	5	53.98	0.063	19.5
2	1	1	55.12	0.071	17.6
2	0	4	62.77	0.066	16.6
1	1	6	68.86	0.071	15.2
2	2	0	70.36	0.076	14.6
2	1	5	75.15	0.074	14.8
3	0	1	76.11	0.071	14.6

Supplementary Figure 1. (top) X-Ray Diffraction pattern of nanoparticulate TiO_2(101). Miller indices are listed above each reflection and indexed to the anatase polymorph. The inset shows a Gaussian fit of the 101 reflection. (bottom) Table of Miller indices taken from the XRD pattern. FWHM signifies full width at half maximum.

Supplementary Figure 2. Raman characterization of nanoparticulate TiO_2(101)

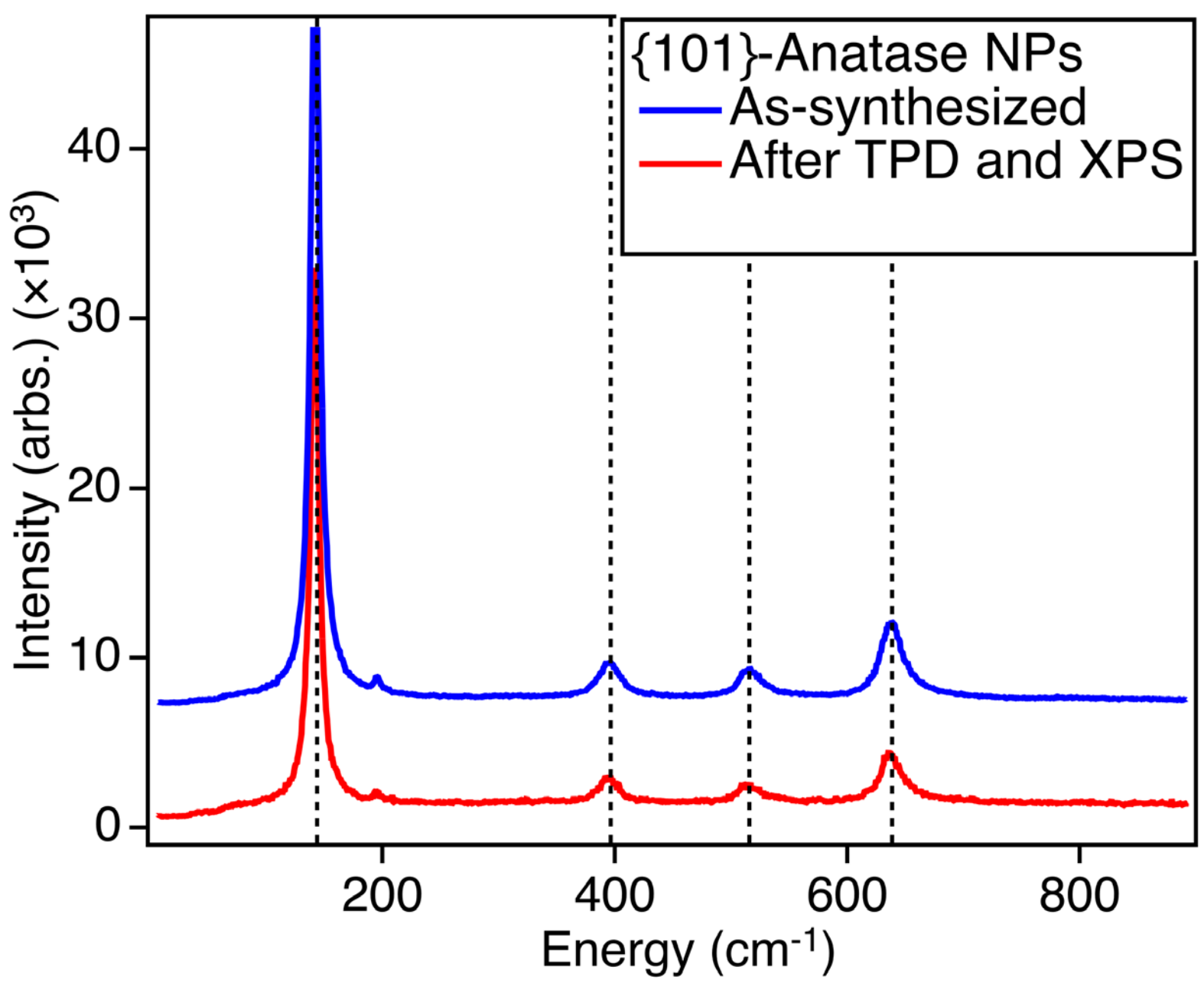

Supplementary Figure 2. Raman spectra of nanocrystalline TiO_2(101). (blue) As-synthesized, (red) after TPD and XPS characterization. The dashed lines indicate the E_g, B_{1g}, A_{1g}+B_{1g}, and E_g modes, respectively.

Supplementary Figure 3. O 1s XPS spectra from nanoparticulate TiO_2(101)

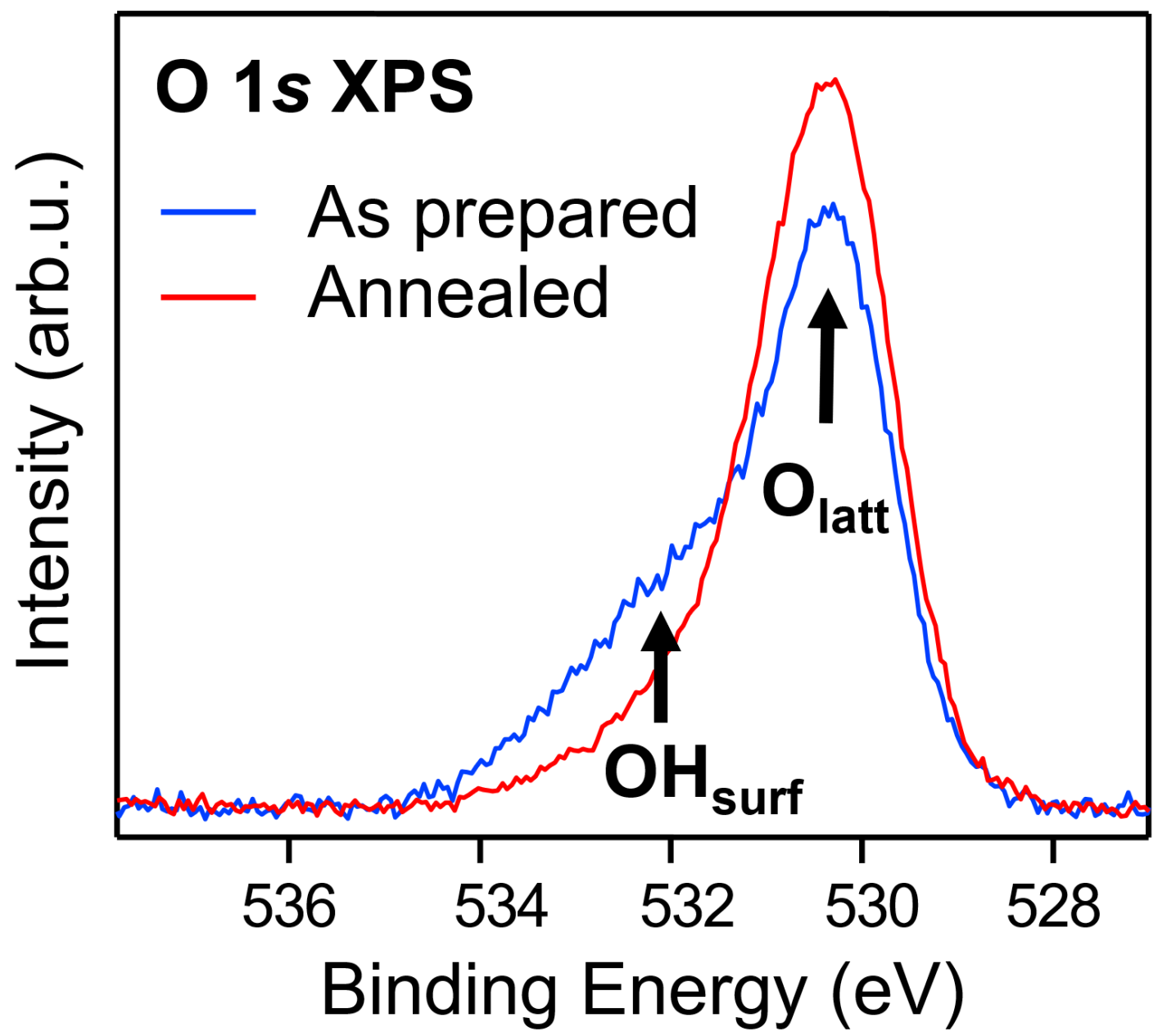

Supplementary Figure 3. O 1*s* XPS spectra before (blue) and after 850 K annealing of the thick nanoparticulate TiO_2(101) layer deposited on optically polished Au-coated Ta substrate obtained under UHV conditions.

Supplementary Figure 4. Coverage-dependent D_2O desorption from planar $TiO_2(101)$

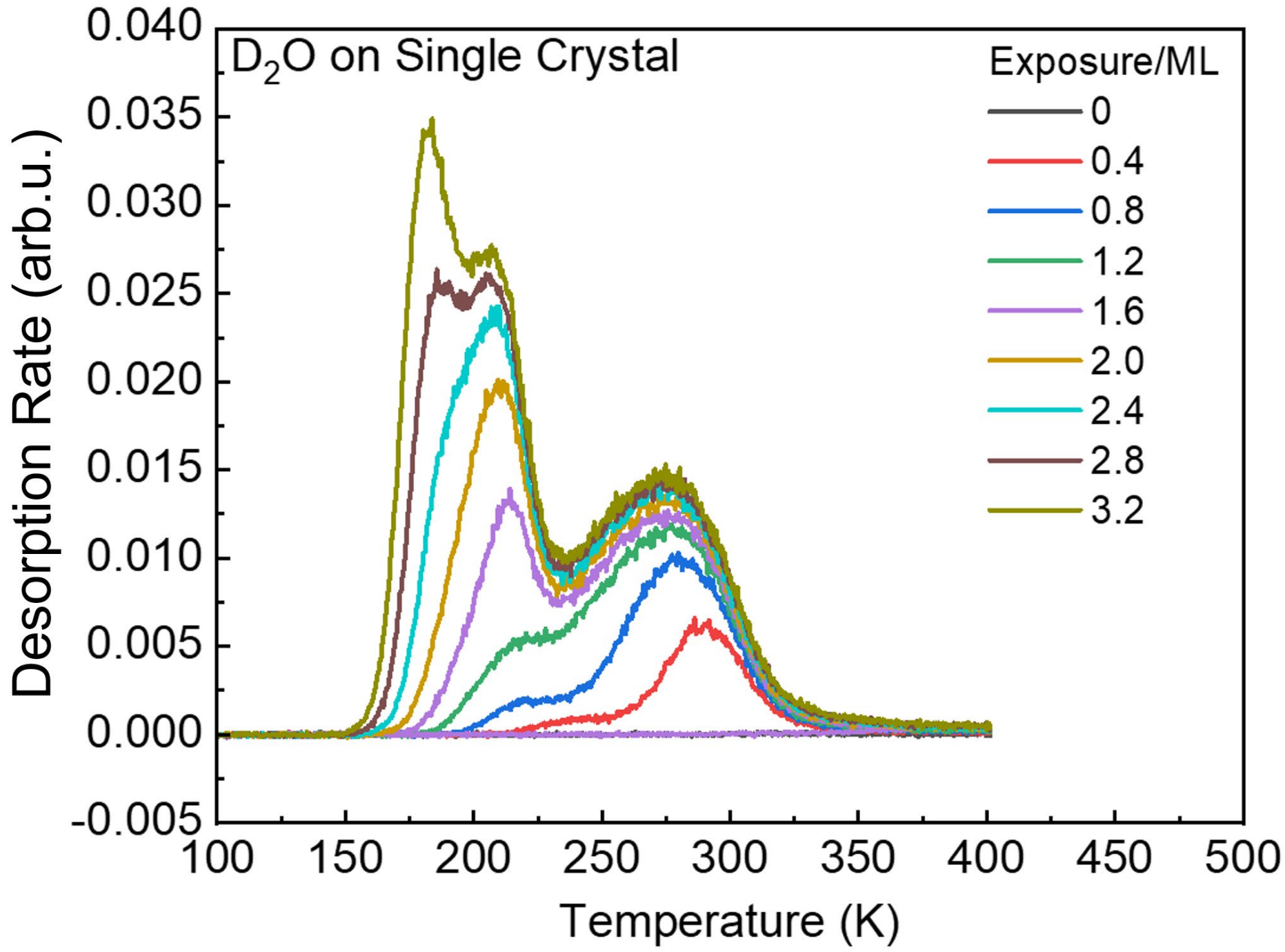

Supplementary Figure 4. Coverage-dependent D_2O TPD spectra following water adsorption at 100 K. D_2O TPD on Anatase $TiO_2(101)$ single crystal. Dosing temperature 100K. The peaks centered at ~280 K, 215 K, and 175 K are assigned to the desorption from Ti_{5c}, O_{2c}, and D_2O multilayers, respectively.

Supplementary Figure 5. D_2O adsorption capacity of TiO_2(101) nanoparticle layers

(a)

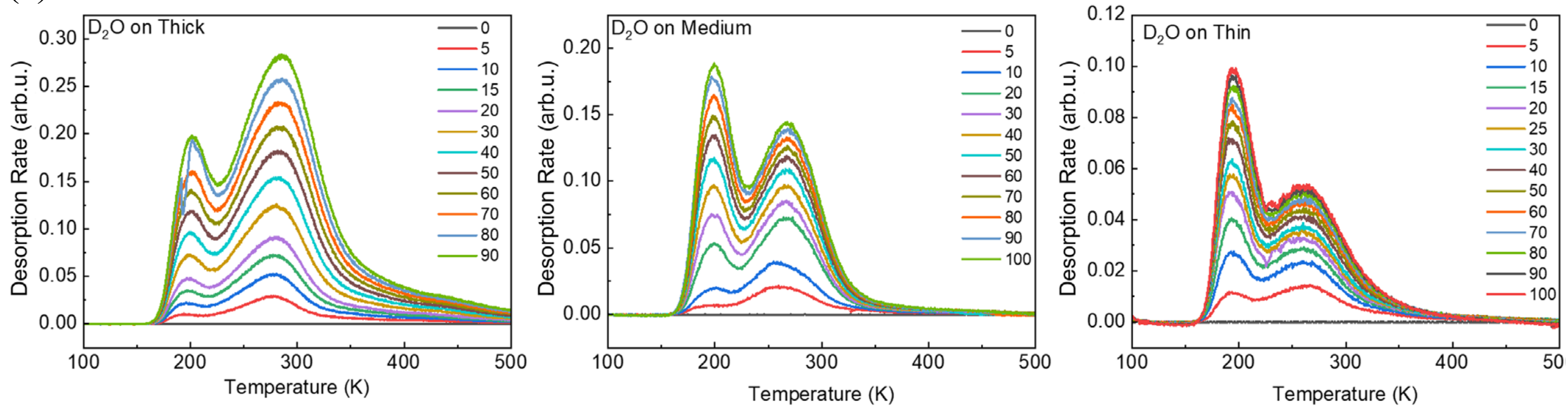

(b)

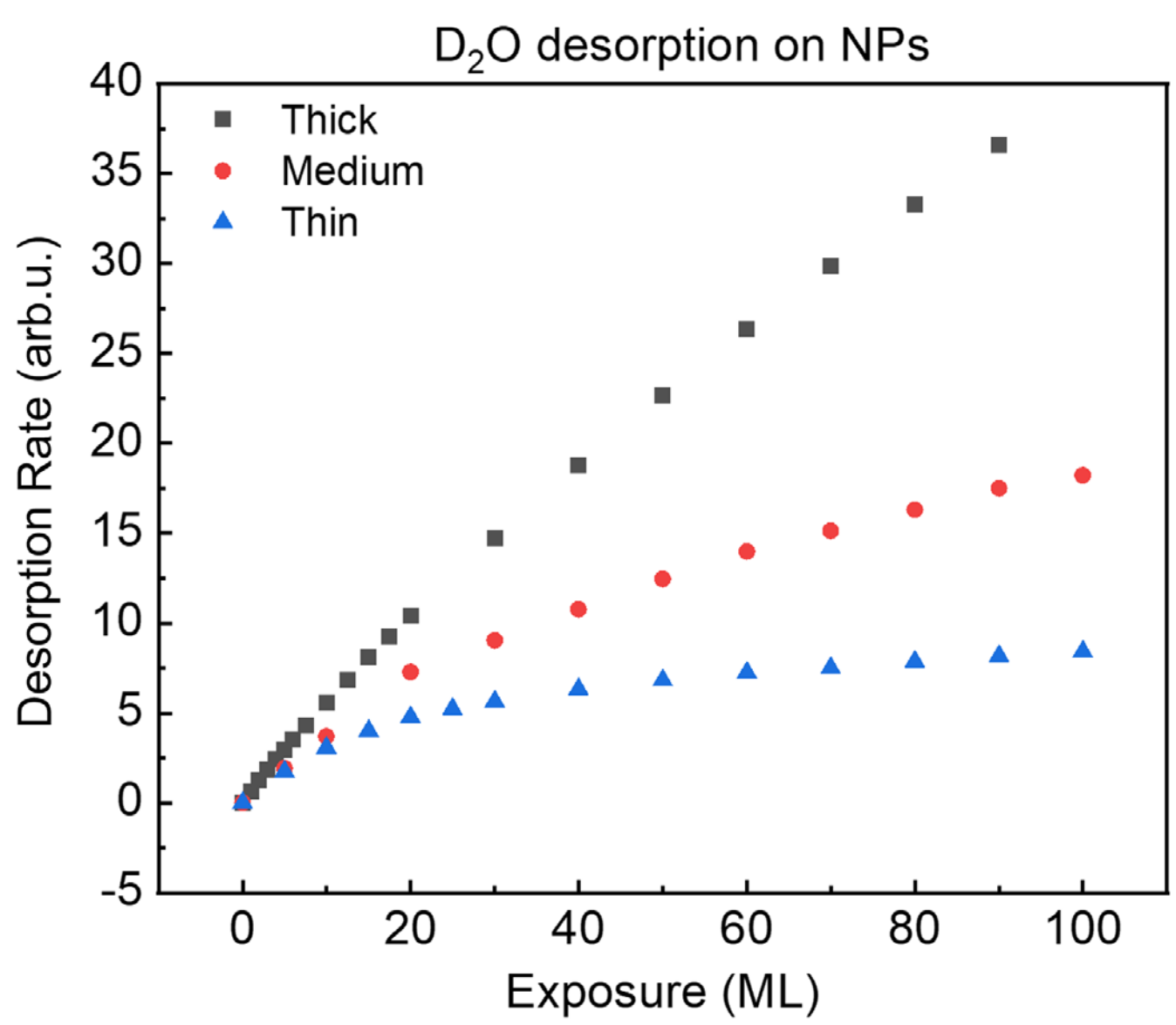

Supplementary Figure 5. (a) D_2O TPD from anatase TiO_2(101) nanoparticles. Exposure is listed in ML-equivalents. Dose temperature was set to175 K to avoid condensation of D_2O multilayers. The D_2O desorption from Ti_{5c} and O_{2c} sites is observed around 280 and 200 K, respectively; the same desorption temperatures as observed on planar TiO_2(101). (b) Integrated area under the dose-dependent TPDs shown in (a). The desorption area from the thick sample does not saturate even for a 100 ML D_2O dose; the medium thickness sample starts saturating around ~20 ML; and the thin samples saturate around ~10 ML.

Supplementary Figure 6. Infrared spectra of acetic acid adsorption on planar and nanoparticulate TiO_2(101)

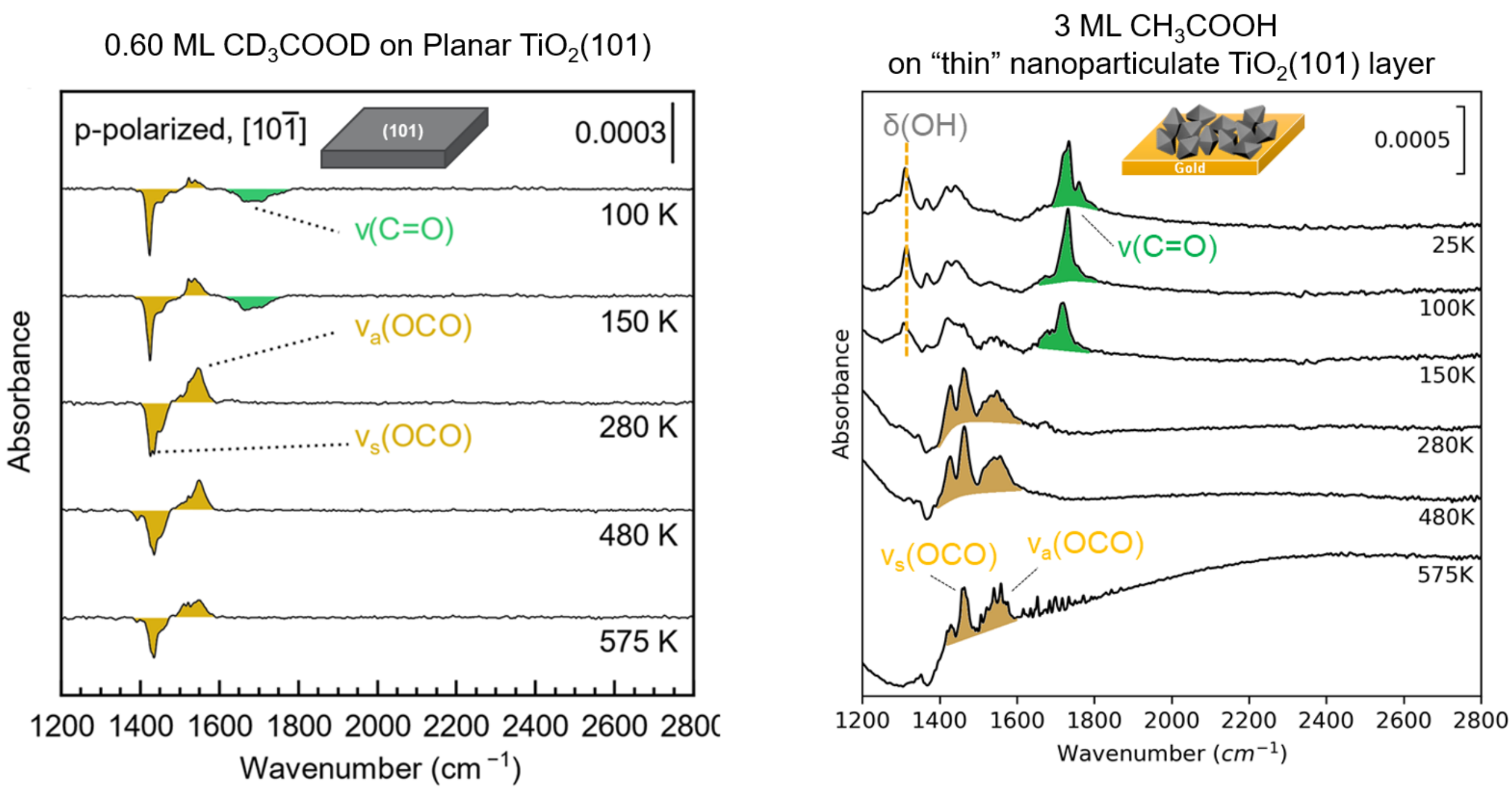

Supplementary Figure 6. Infrared reflection absorption spectra probed with p-polarized light from 0.60 ML of CD_3COOD adsorbed on anatase TiO_2(101) at 100 K (left), and 3 ML of CH_3COOH adsorbed on a "Thin" layer of TiO_2(101) nanoparticles at 25 K (right). Each sample was flash annealed to 100, 150, 280, 480, and 575 K. The vibrational bands associated with bidentate acetate (yellow) are ν_s(OCO) and ν_a(OCO), while the band associated with monodentate acetate (green) is ν(C=O). This indicates that on nanoparticles, acetic acid has the same binding configuration as on anatase TiO_2(101) single crystal. Left panel reproduced with permission from Springer Nature. C. R. O'Connor, R. Ma, G. Collinge, M.-S. Lee, G. A. Kimmel and Z. Dohnálek, "Insights into Acetic Acid Binding and Ketene Formation on Anatase TiO_2(101)," *Topics in Catalysis*, 66, 1087–1101, 2023, Springer Nature.

Supplementary Figure 7. Product-resolved TPRS of acetic acid on planar TiO_2(101)

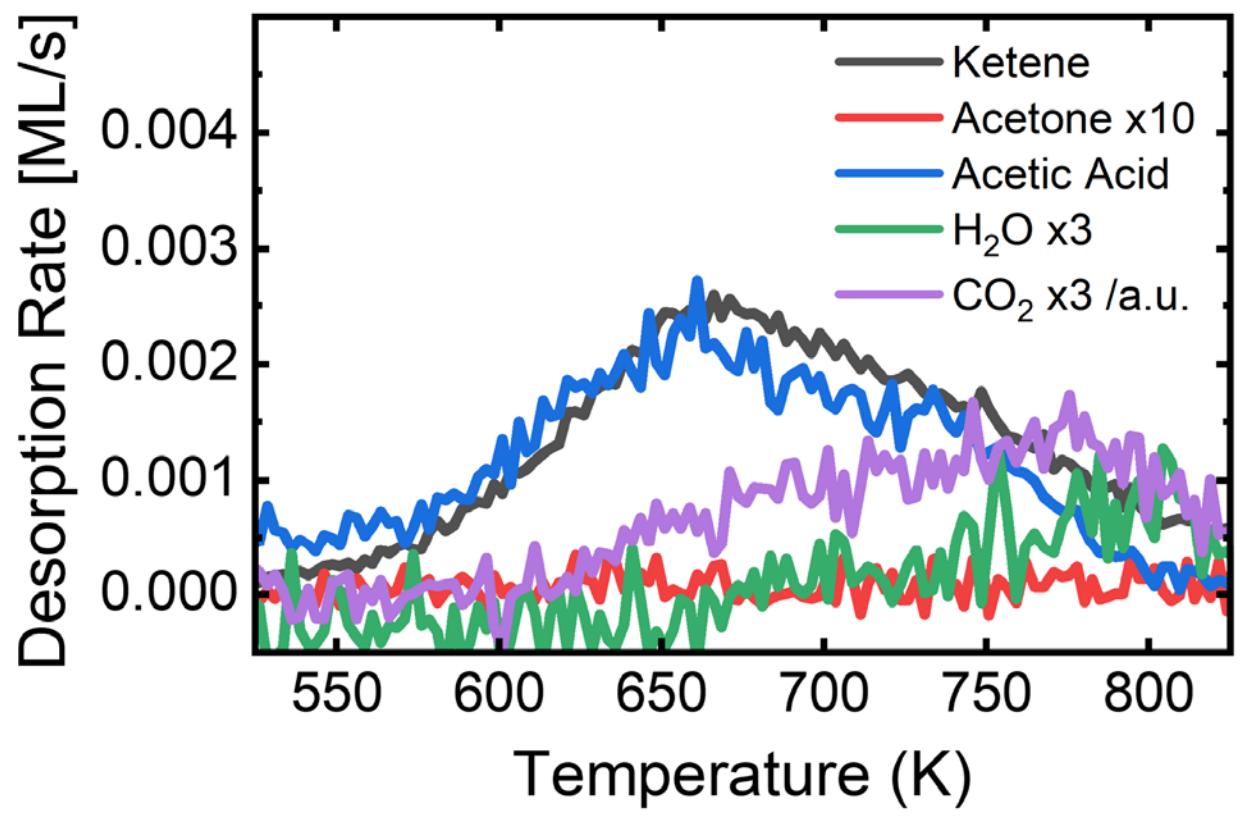

Supplementary Figure 7. TPRS spectra of all products, ketene, acetone, recombinatively-formed acetic acid, water, and CO_2, obtained following a 2 ML dose of acetic acid at 100 K under UHV conditions on planar TiO_2(101).

Supplementary Figure 8. Acetic acid coverage-dependent TPRS on planar $TiO_2(101)$

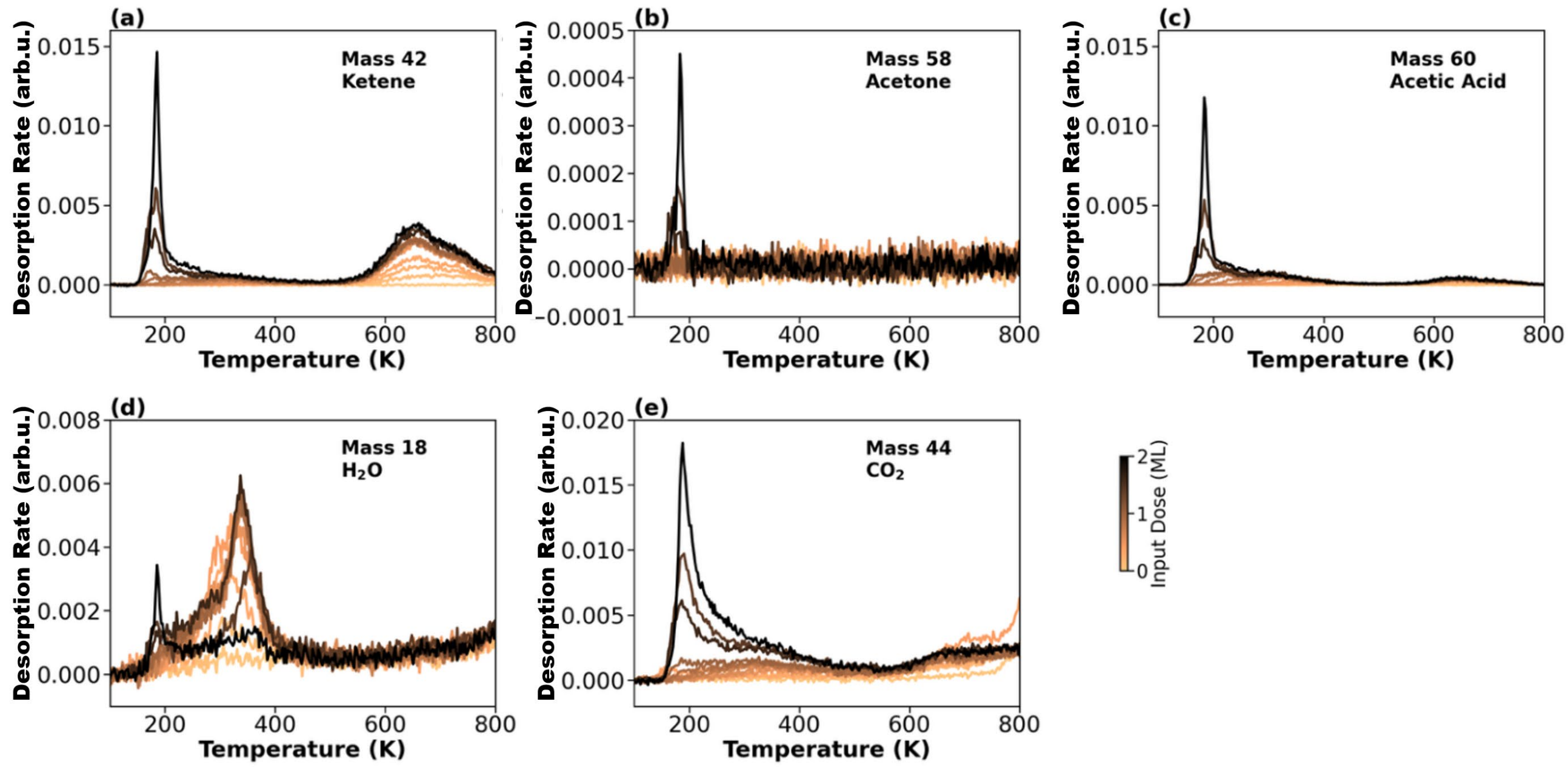

Supplementary Figure 8. Acetic acid coverage-dependent TPRS spectra of (a) ketene, m/z 42; (b) acetone, m/z 58; (c) acetic acid, m/z 60; (d) H_2O, m/z 18; (e) CO_2, m/z 44 obtained following acetic acid dose at 100 K under UHV conditions on planar $TiO_2(101)$.

Supplementary Figure 9. Product-resolved TPRS of acetic acid on a thick nanoparticulate TiO_2(101) layer

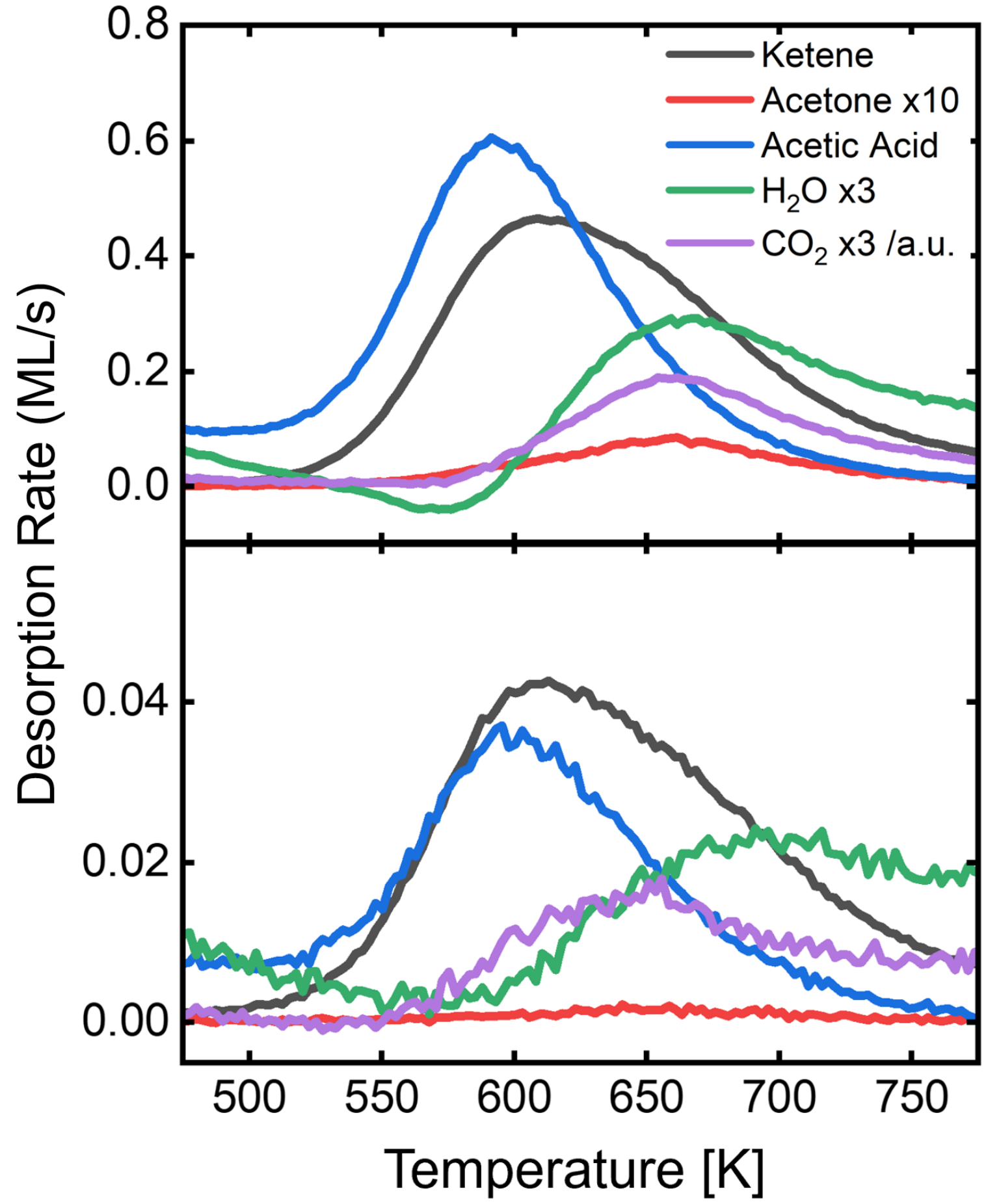

Supplementary Figure 9. TPRS spectra of all products, ketene, acetone, recombinatively formed acetic acid, water, and CO_2, obtained following acetic acid dose on a "thick" layer of TiO_2(101) nanoparticles at 100 K conditions (10ML_{eq}, bottom and 90 ML_{eq}, top).

Supplementary Figure 10. Acetic acid dose-dependent TPRS on a thick TiO_2(101) nanoparticle layer

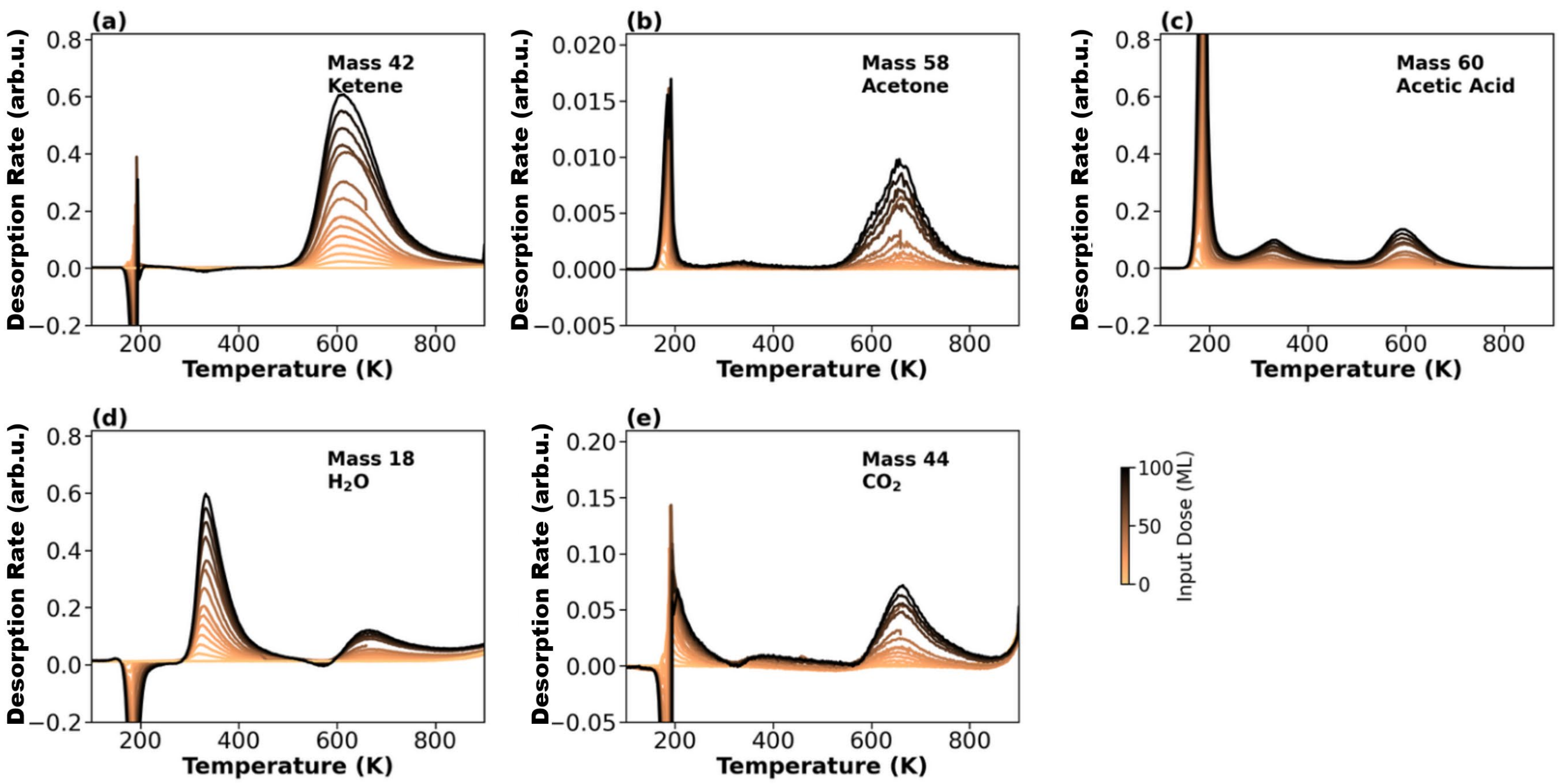

Supplementary Figure 10. Acetic acid dose (0 - 100 ML-eq) dependent TPRS spectra of (a) ketene, m/z 42; (b) acetone, m/z 58; (c) acetic acid, m/z 60; (d) H_2O, m/z 18; (e) CO_2, m/z 44 from "thick" layer of TiO_2(101) nanoparticles. Acetic acid was dosed at 100 K.

Supplementary Figure 11. Acetic acid dose-dependent TPRS on a medium TiO_2(101) nanoparticle layer

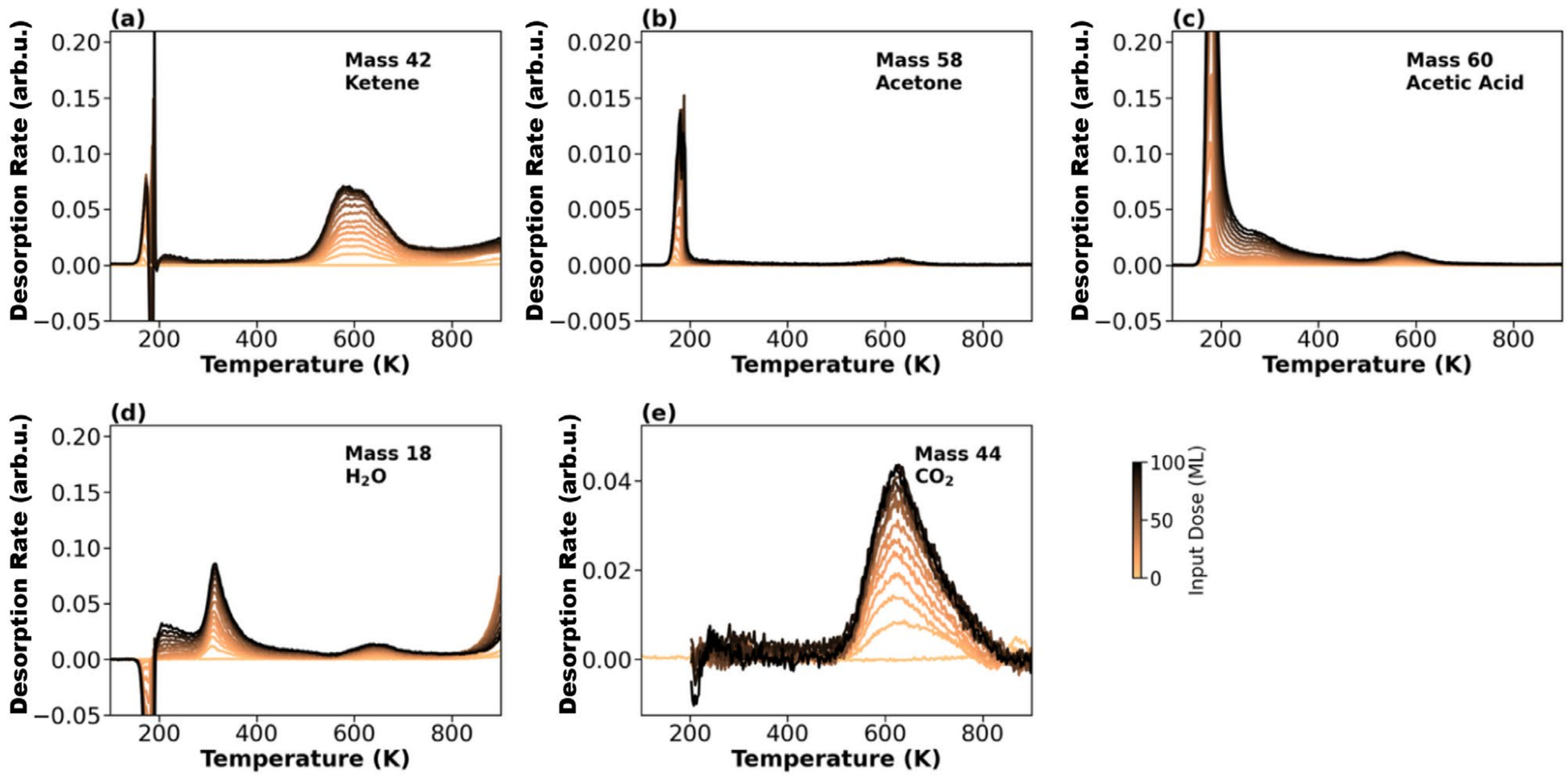

Supplementary Figure 11. Acetic acid dose-dependent (0 - 100 ML-eq) TPRS spectra of (a) ketene, m/z 42; (b) acetone, m/z 58; (c) acetic acid, m/z 60; (d) H_2O, m/z 18; (e) CO_2, m/z 44 from "medium" layer of TiO_2(101) nanoparticles. Acetic acid was dosed at 100 K.

Supplementary Figure 12. Acetic acid dose-dependent TPRS on a thin TiO_2(101) nanoparticle layer

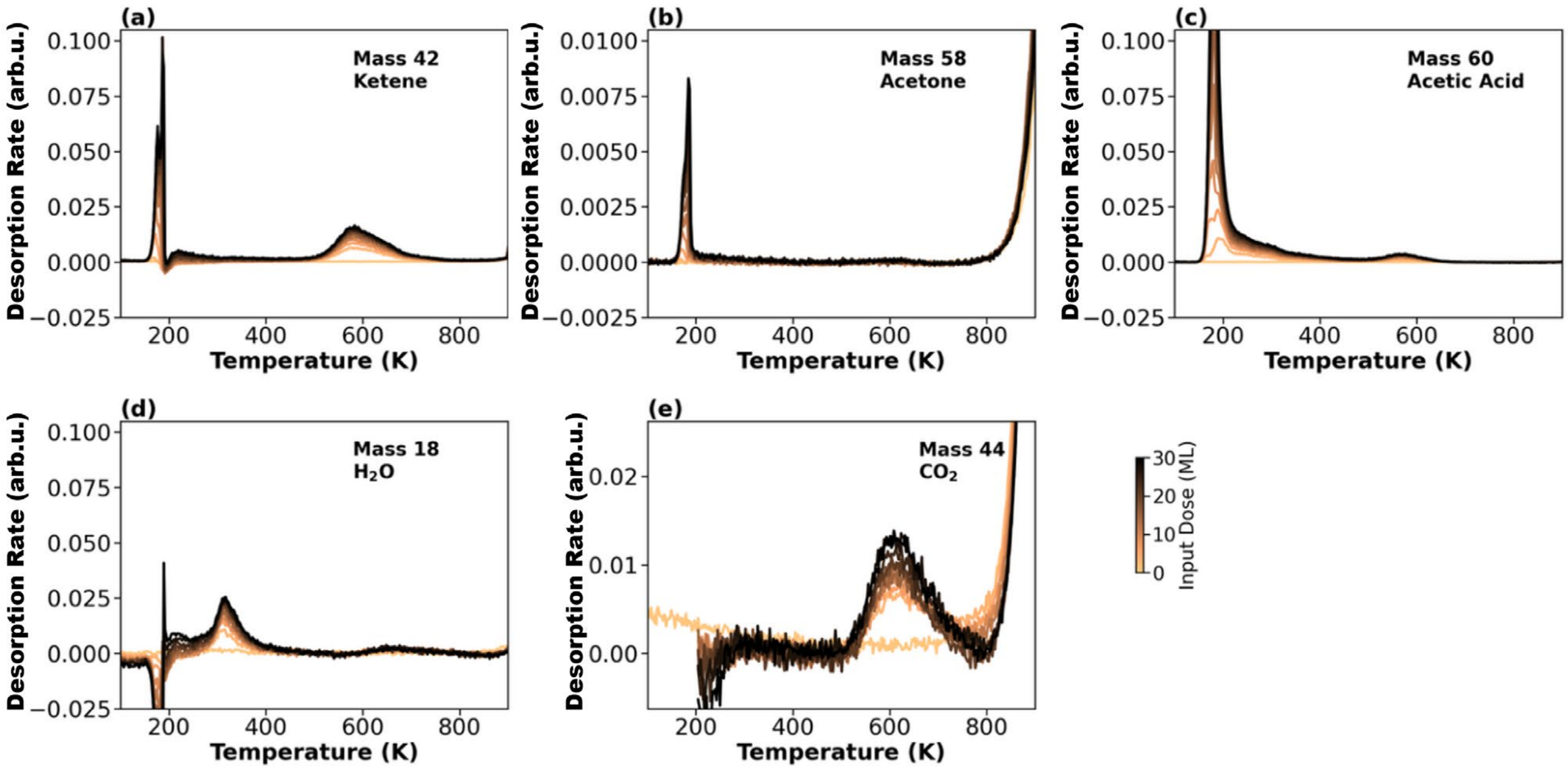

Supplementary Figure 12. Acetic acid dose-dependent (0 - 100 ML-eq) TPRS spectra of (a) ketene, m/z 42; (b) acetone, m/z 58; (c) acetic acid, m/z 60; (d) H_2O, m/z 18; (e) CO_2, m/z 44 from "thin" layer of TiO_2(101) nanoparticles. Acetic acid was dosed at 100 K.

Supplementary Figure 13. Product yields as a function of TiO_2(101) nanoparticle-layer thickness and acetic acid exposure

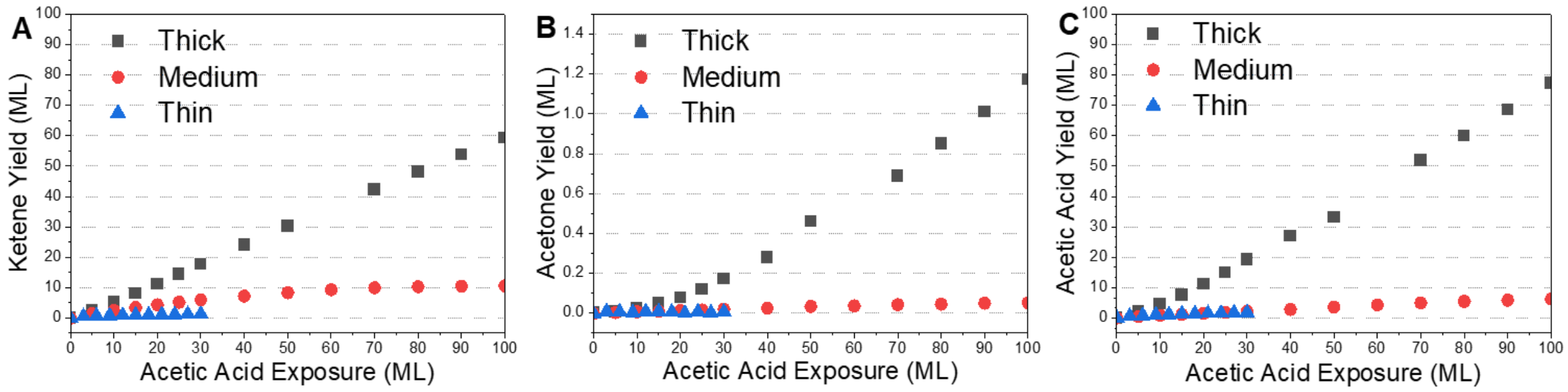

Supplementary Figure 13. Integrated yields of A, ketene; B, acetone; C, recombinatively-desorbed acetic acid from TPRS spectra shown in Supplementary Figures 10-12. While ketene and acetic acid were produced from initial dosage, acetone was not produced until 10 ML of coverage.

Supplementary Figure 14. CD_3COOD dose-dependent TPRS on a medium $TiO_2(101)$ nanoparticle layer.

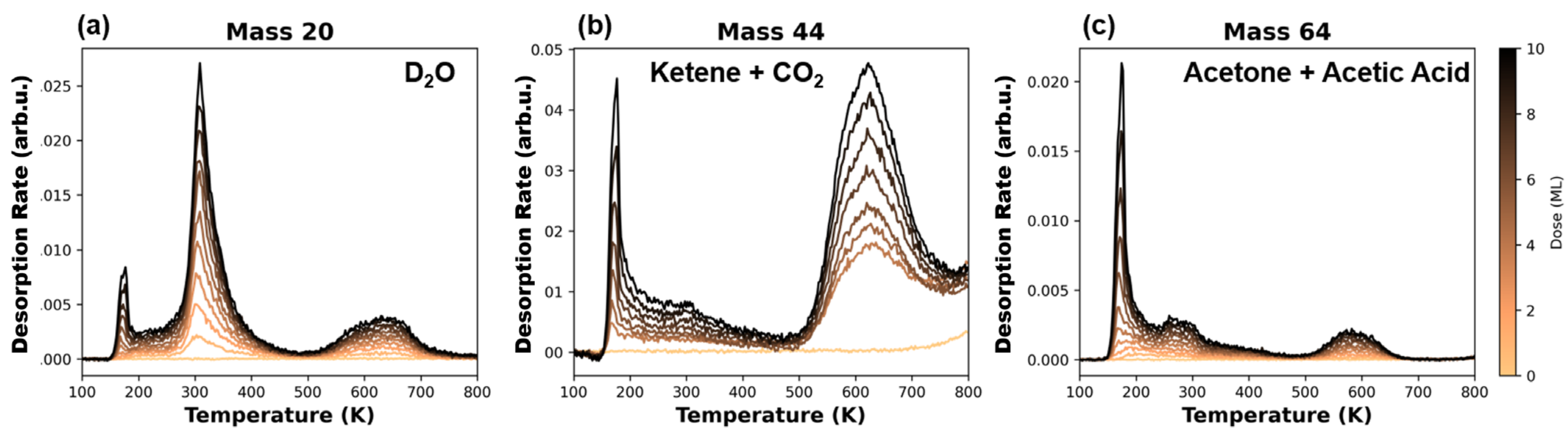

Supplementary Figure 14. CD_3COOD coverage-dependent TPRS of (a) D_2O at m/z 20; (b) CD_2CO and CO_2 at m/z 44; (c) CD_3COCD_3 and CD_3COOD m/z = 64; from "medium" layer of $TiO_2(101)$ nanoparticles, obtained following a series of CD_3COOD doses at 100 K under UHV conditions. D-labeled ketene (CD_2CO) has the same mass as CO_2, and D-labeled acetone (CD_3COCD_3) has the same mass as D-labeled acetic acid (CD_3COOD).

Supplementary Figure 15. Anatase TiO_2(101) slab model used in the DFT calculations

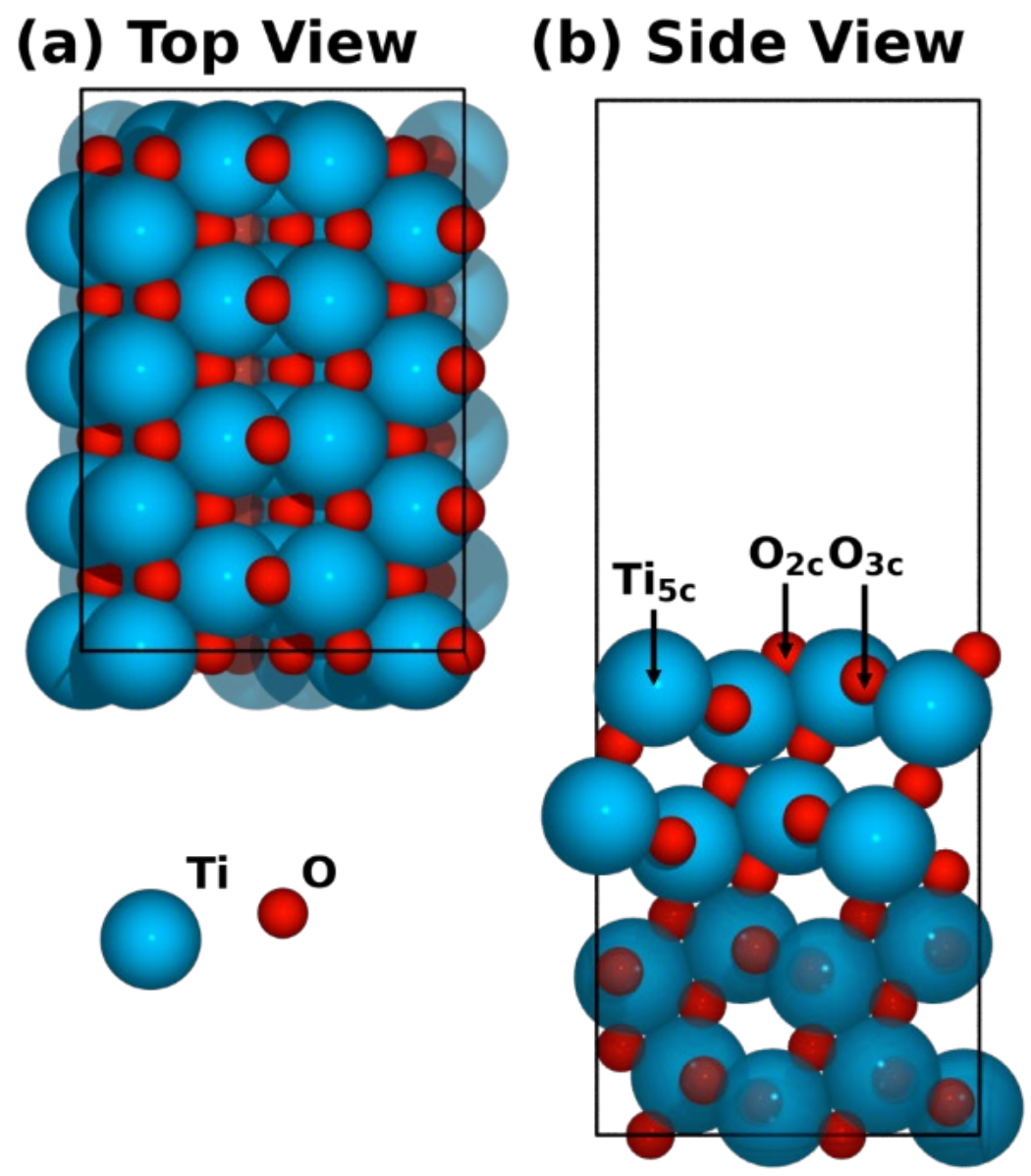

Supplementary Figure 15. Top (a) and side (b) views of the $p(1 \times 4)$-TiO_2(101) slab model. The slab contains four Ti layers comprising 64 TiO_2 units (192 atoms) and a ~15 Å vacuum region along the z direction to avoid spurious interactions between periodic images. The surface layer exposes eight five-coordinated Ti sites (Ti_{5c}), eight two-coordinated O sites (O_{2c}), and eight three-coordinated O sites (O_{3c}). The bottom half of the slab (96 atoms, shown transparently) was fixed during the calculations. Ti and O atoms are shown in cyan and red, respectively.

Supplementary Figure 16. Acetic acid adsorption configurations and interconversion pathways on anatase TiO_2(101)

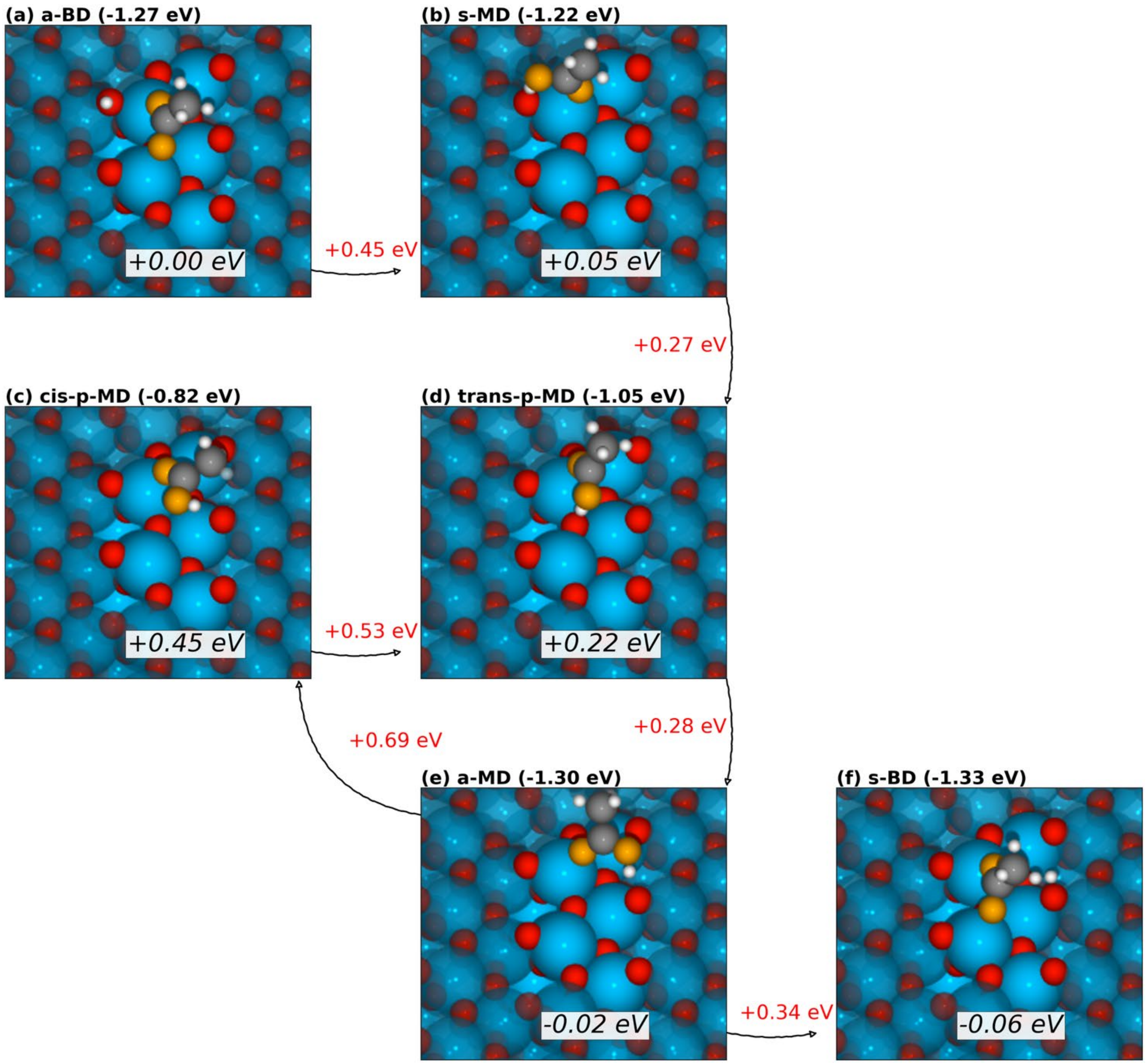

Supplementary Figure 16. DFT-optimized adsorption configurations of acetic acid on p(1 × 4)-TiO_2(101): (a) asymmetric bidentate acetate (a-BD), (b) symmetric monodentate acetic acid (s-MD), (c) cis parallel monodentate acetic acid (cis-p-MD), (d) trans parallel monodentate acetic acid (trans-p-MD), (e) asymmetric monodentate acetic acid (a-MD), and (f) symmetric bidentate acetate (s-BD).

Molecular adsorption produces monodentate acetic acid bound to one Ti_{5c} site, whereas dissociative adsorption produces bidentate acetate and H*, occupying two Ti_{5c} sites and a neighboring O_{2c} site. The prefixes a, s, and p denote asymmetric, symmetric, and parallel orientations relative to the surface x-axis, respectively, while cis and trans specify the molecular configuration. In a-BD and s-BD, acetate binds through both O atoms to adjacent Ti_{5c} sites, with the configurations differing in the location of H* on O_{2c}. The monodentate configurations differ primarily in the orientation of the OH group and its hydrogen-bonding interaction with O_{2c} or O_{3c} sites. a-BD converts to s-MD with a barrier of +0.45 eV; subsequent rotation gives trans-p-MD with a barrier of +0.22 eV. trans-p-MD can convert to cis-p-MD with a barrier of +0.31 eV, whereas the reverse barrier is only +0.08 eV. a-MD can dissociate to s-BD with a barrier of +0.36 eV or convert to cis-p-MD with a higher barrier of +0.71 eV. Values in parentheses above each panel are adsorption energies referenced to gas-phase acetic acid and the clean surface. Boxed values are relative energies, and red values adjacent to the arrows are transition-state energies, all referenced to a-BD.

Supplementary Figure 17. Total- and free-energy profiles for ketene and acetone formation under UHV and higher-pressure conditions

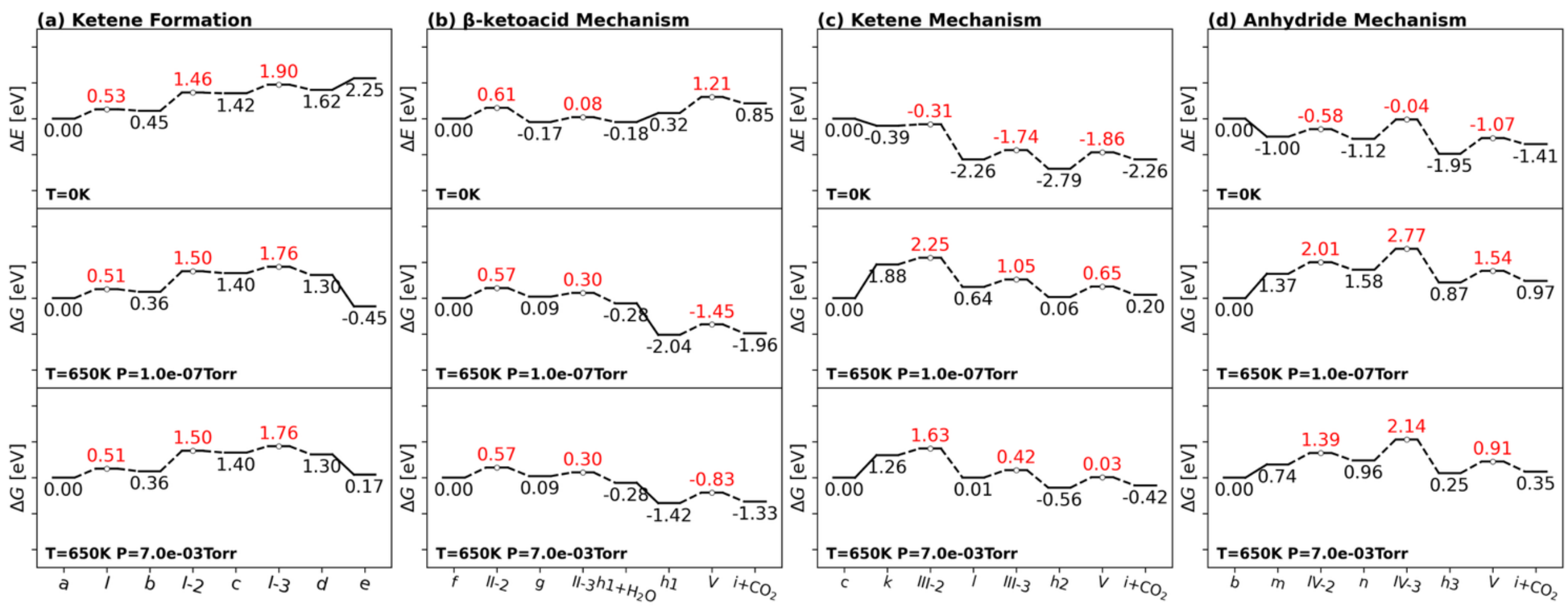

Supplementary Figure 17. Total-energy (ΔE) and free-energy (ΔG) profiles predicted by DFT for (a) ketene formation, (b) acetone formation through the β-ketoacid mechanism, (c) acetone formation through the ketene mechanism, and (d) acetone formation through the anhydride mechanism. Total-energy profiles are shown at 0 K, and free-energy profiles are shown at 650 K and 1×10^{-7} Torr (UHV conditions) and at 650 K and 7×10^{-3} Torr (higher-pressure conditions), both corresponding to an acetic acid conversion of 50% and an acetone yield of 1%. The free-energy and total-energy profiles are similar except for the adsorption and desorption steps. Letters along the abscissas denote the intermediates, and Roman numerals denote the transition steps illustrated in main-text Figure 3. Numerical values indicate the energies of intermediates (black) and transition states (red), referenced to the initial structure of each pathway.

Supplementary Figure 18. Ketene adsorption configurations and interconversion pathways

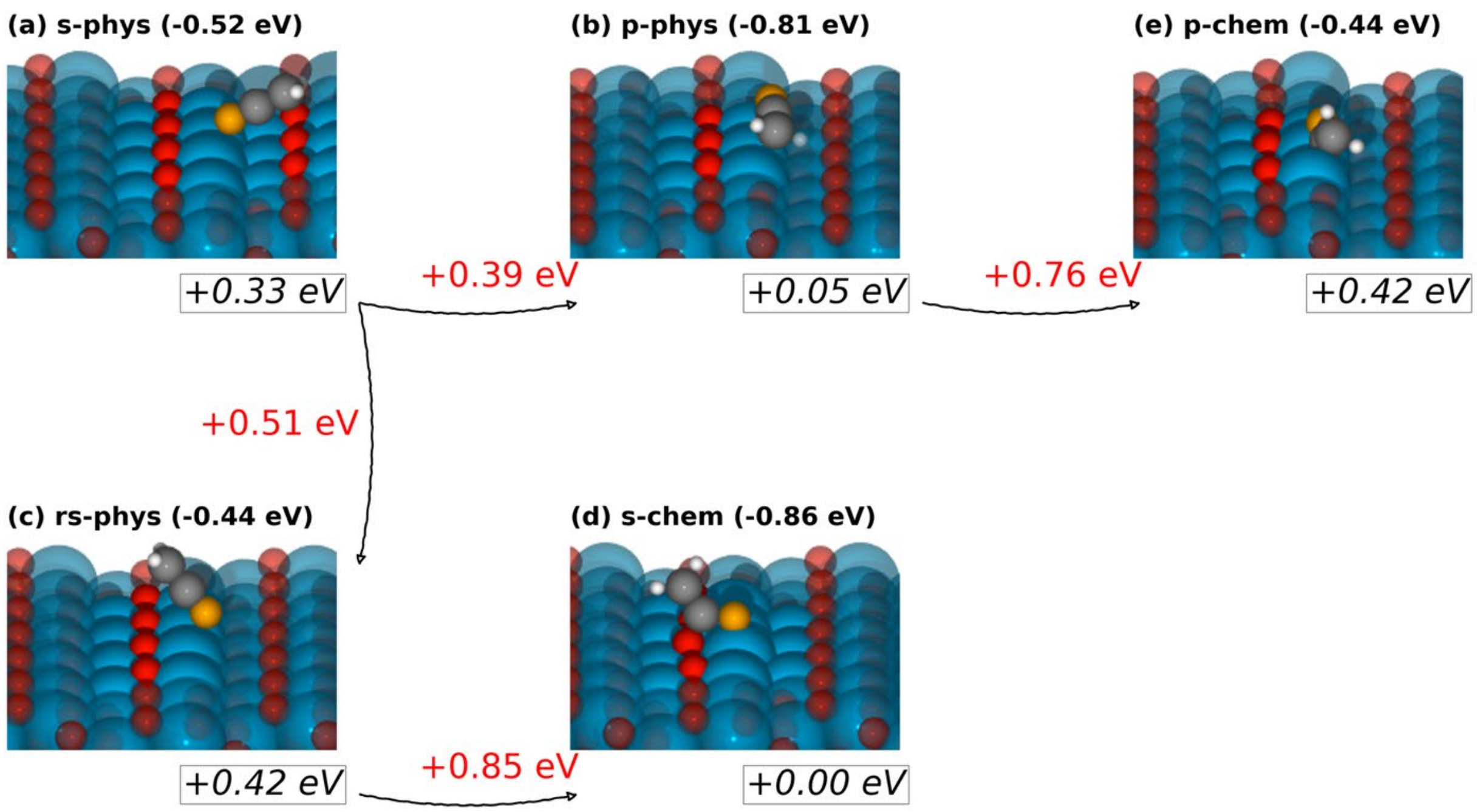

Supplementary Figure 18. DFT-optimized structures and relative energies of five ketene adsorption configurations: (a) s-phys, (b) p-phys, (c) rs-phys, (d) s-chem, and (e) p-chem. The prefixes p and s indicate ketene orientations parallel to the y-axis and symmetric with respect to the x-axis, respectively; rs denotes a reversed s-type orientation, while phys and chem denote physical and chemical adsorption. In the physically adsorbed configurations (a–c), ketene remains more than 2.0 Å from the surface without forming chemical bonds and is stabilized primarily by van der Waals interactions. The p-phys configuration is 0.28 eV more stable than s-phys. In the chemically adsorbed configurations (d, e), the C–C–O backbone bends upon interaction of the carbonyl C atom with a surface O atom, while the ketene O atom binds to a nearby Ti_{5c} site. In p-chem, the C atom interacts with O_{3c}, whereas in s-chem it interacts with O_{2c}, making s-chem the most stable configuration with an adsorption energy of −0.86 eV. Values in parentheses above each panel are adsorption energies referenced to non-interacting gas-phase ketene and the clean surface. Boxed values are relative energies, and red values above the arrows are transition-state energies, all referenced to s-chem. Conversion from physically adsorbed s-phys to s-chem proceeds through rs-phys with an effective total-energy barrier of +0.52 eV.

Supplementary Figure 19. Direct ketene formation from molecular acetic acid at high surface coverage.

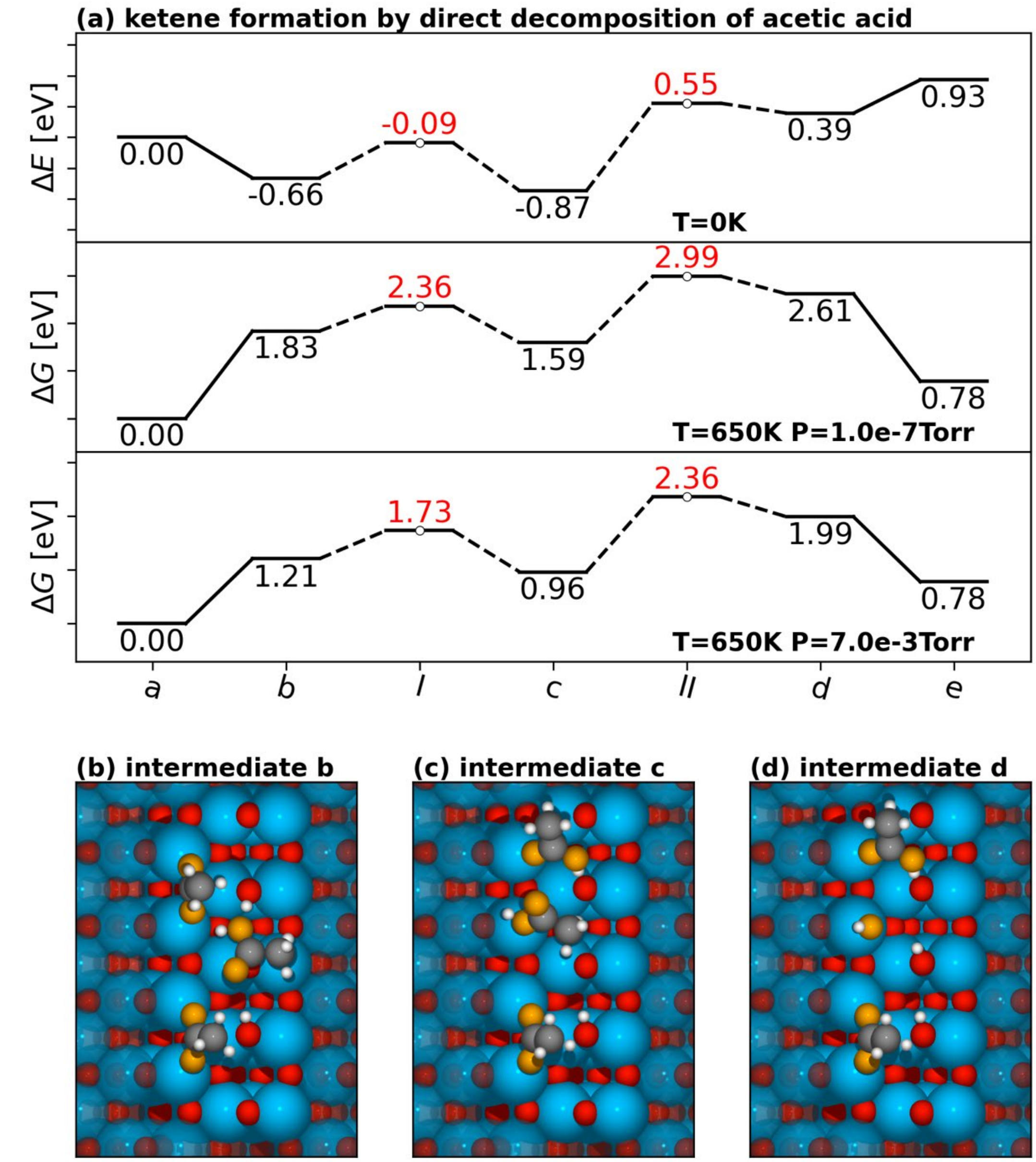

Supplementary Figure 19. (a) Total-energy (ΔE) and free-energy (ΔG) profiles for direct decomposition of molecular acetic acid, shown at 0 K and at 650 K under 1×10^{-7} Torr and 7×10^{-3} Torr. Energies are referenced to the initial state containing two adsorbed bidentate acetates. At high acetic acid coverage, an additional acetic acid molecule can adsorb in an adlayer above the surface, where it is stabilized by hydrogen bonding. In intermediate b, the adlayer acetic acid lies

approximately parallel to the surface and forms hydrogen bonds with a bidentate acetate, with an adsorption energy of −0.66 eV. Rearrangement to intermediate c produces a weak Ti_{5c}–OH interaction while one bidentate acetate converts to a monodentate configuration; this rearrangement has a total-energy barrier of +0.57 eV. Subsequent concerted C–O and C–H bond cleavage produces OH*, an O_{2c}-bound H*, and physically adsorbed ketene and proceeds from intermediate c with a total-energy barrier of +1.42 eV. The nascent ketene in intermediate d has an adsorption energy of −0.54 eV. This direct decomposition pathway is analogous to that reported for formic acid on TiO_2, although formic acid decomposition produces CO rather than ketene.[6] (b) Two bidentate acetates and one nonchemically adsorbed acetic acid in the adlayer. (c) Rearranged configuration containing monodentate acetic acid prior to decomposition. (d) Products following direct decomposition of the adlayer acetic acid.

Supplementary Figure 20. Ketene formation through an acylium intermediate.

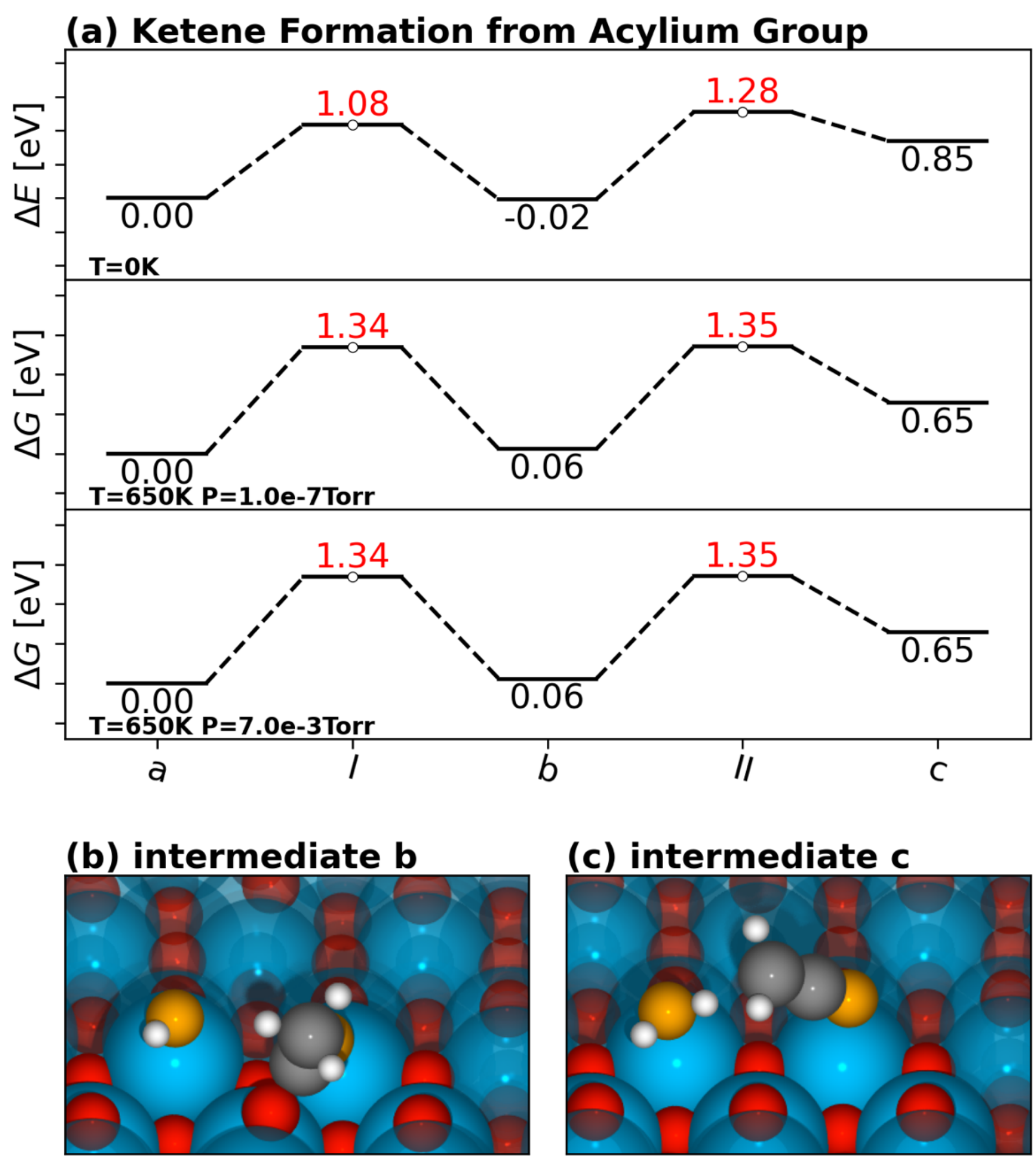

Supplementary Figure 20. (a) Total-energy (Δ*E*) and free-energy (Δ*G*) profiles for ketene formation through an acylium pathway, shown at 0 K and at 650 K under 1×10^{-7} Torr and 7×10^{-3} Torr. The pathway begins from monodentate acetic acid in the cis-p-MD configuration (structure a). C–O bond cleavage (step I) forms an adsorbed acylium intermediate together with an OH group (structure b), followed by α-H abstraction (step II) to form ketene and adsorbed H_2O (structure c). Energies are referenced to the initial monodentate acetic acid configuration. (b) Structure of the adsorbed acylium intermediate and OH group. (c) Structure of the resulting ketene and adsorbed H_2O.

Supplementary Figure 21. Decarboxylation transition states of deprotonated β-ketoacid intermediates.

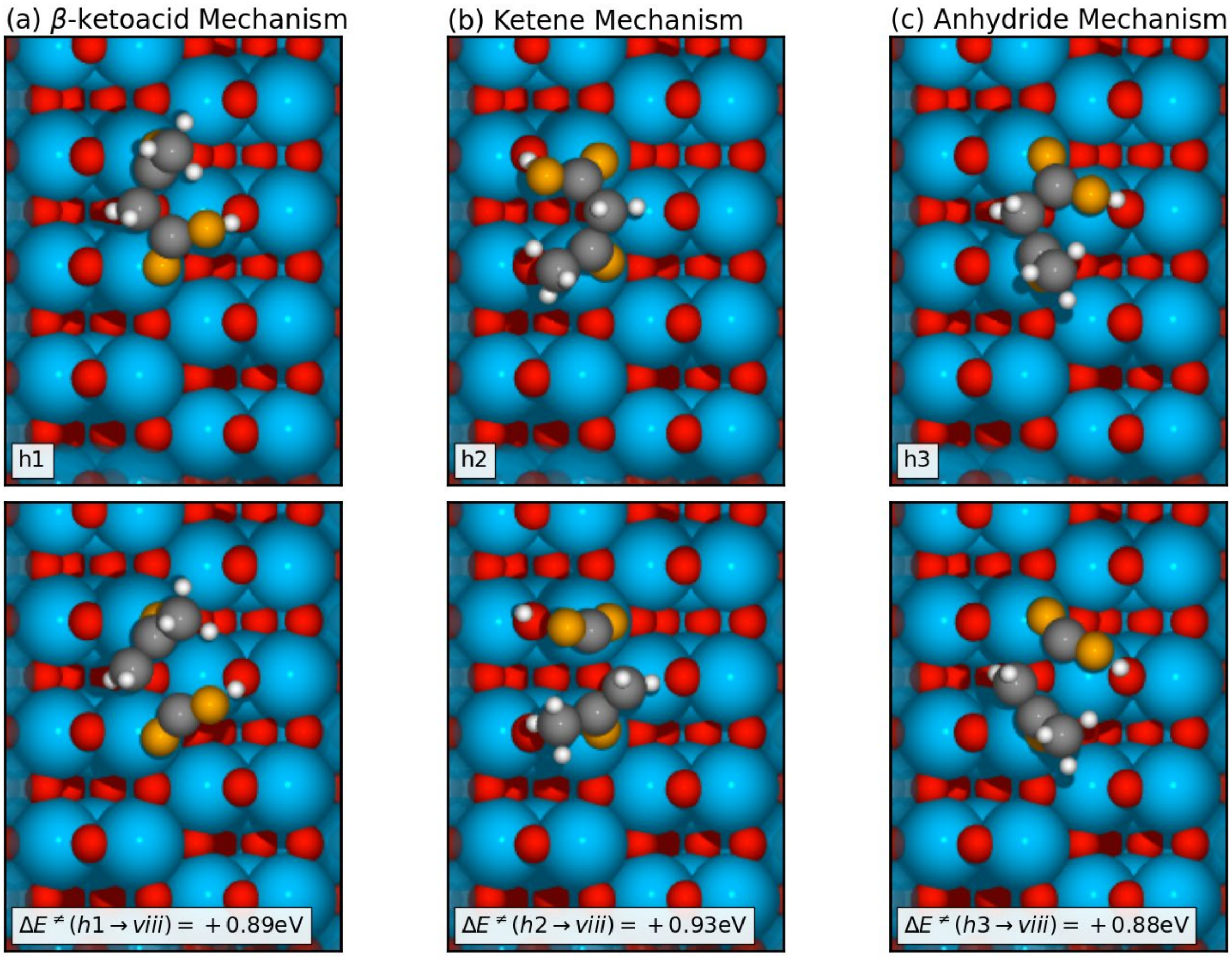

Supplementary Figure 21. Panels (a–c) show the optimized deprotonated β-ketoacid intermediates h1-h3 (top) and their corresponding decarboxylation transition states (bottom) for the β-ketoacid, ketene, and anhydride mechanisms, respectively. Although the three pathways converge to the same chemical intermediate, the adsorbates retain distinct adsorption geometries inherited from the preceding reaction steps. These geometric differences result in slightly different transition-state structures and calculated activation barriers of +0.89, +0.93, and +0.88 eV for h1, h2, and h3, respectively, as indicated in the lower panels.

Supplementary Figure 22. Intermolecular dehydration of a β-ketoacid intermediate

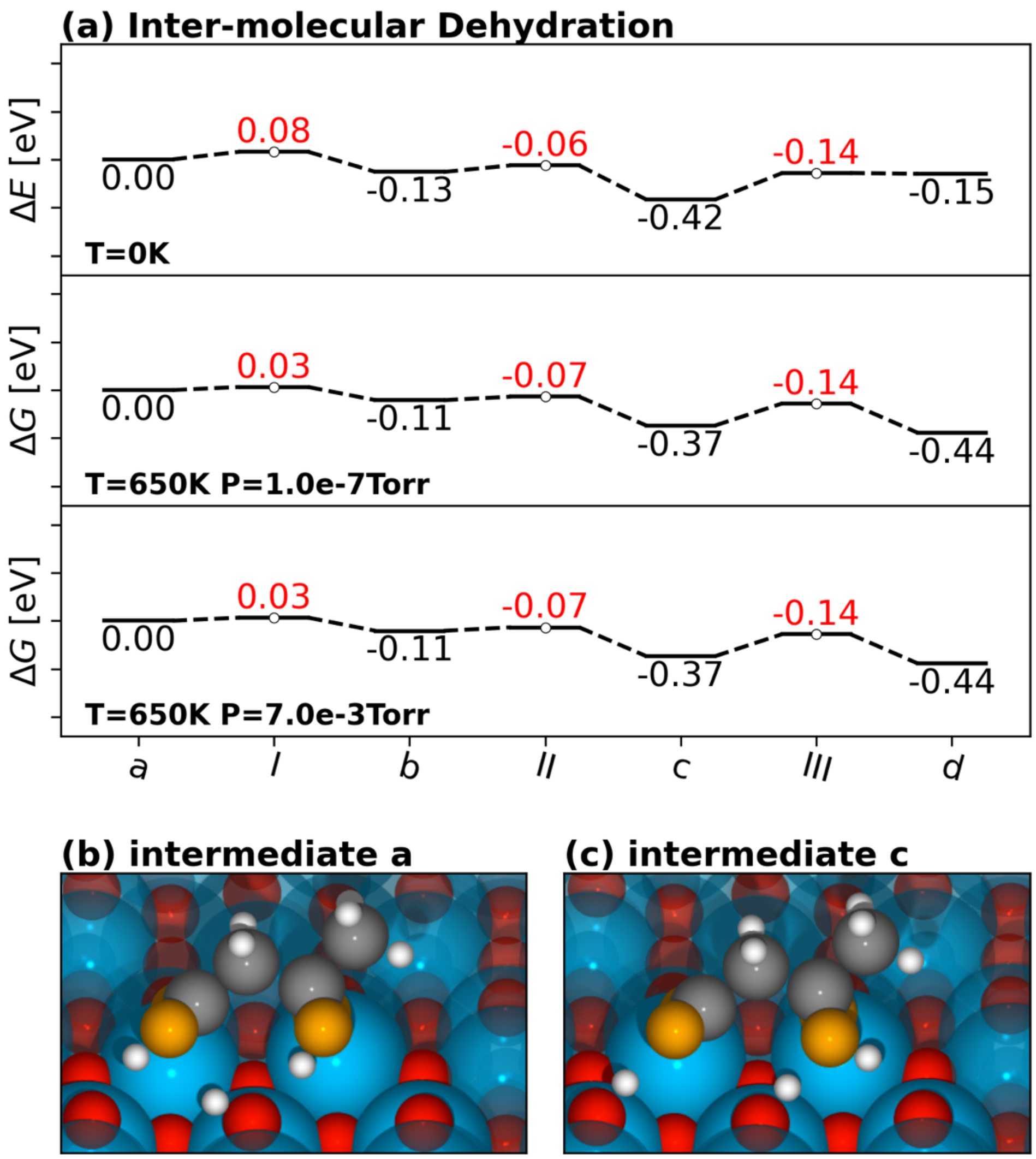

Supplementary Figure 22. (a) Total-energy (ΔE) and free-energy (ΔG) profiles for the intermolecular dehydration of a β-ketoacid at 0 K and at 650 K under 1×10^{-7} Torr and 7×10^{-3} Torr. The β-ketoacid is formed by coupling a monodentate acetic acid with a 1-hydroxyl enolate. An H atom is transferred from the enolate side of the β-ketoacid to an O_{2c} site (step I), followed by reorganization of the hydrogen bond between the OH group and H* (step II). The resulting deprotonated β-ketoacid then undergoes dehydration (step III). (b) Structure of the β-ketoacid intermediate formed by coupling monodentate acetic acid and a 1-hydroxyl enolate. (c) Structure of the deprotonated β-ketoacid intermediate.

Supplementary Figure 23. Protonation of acetone enolate to form acetone

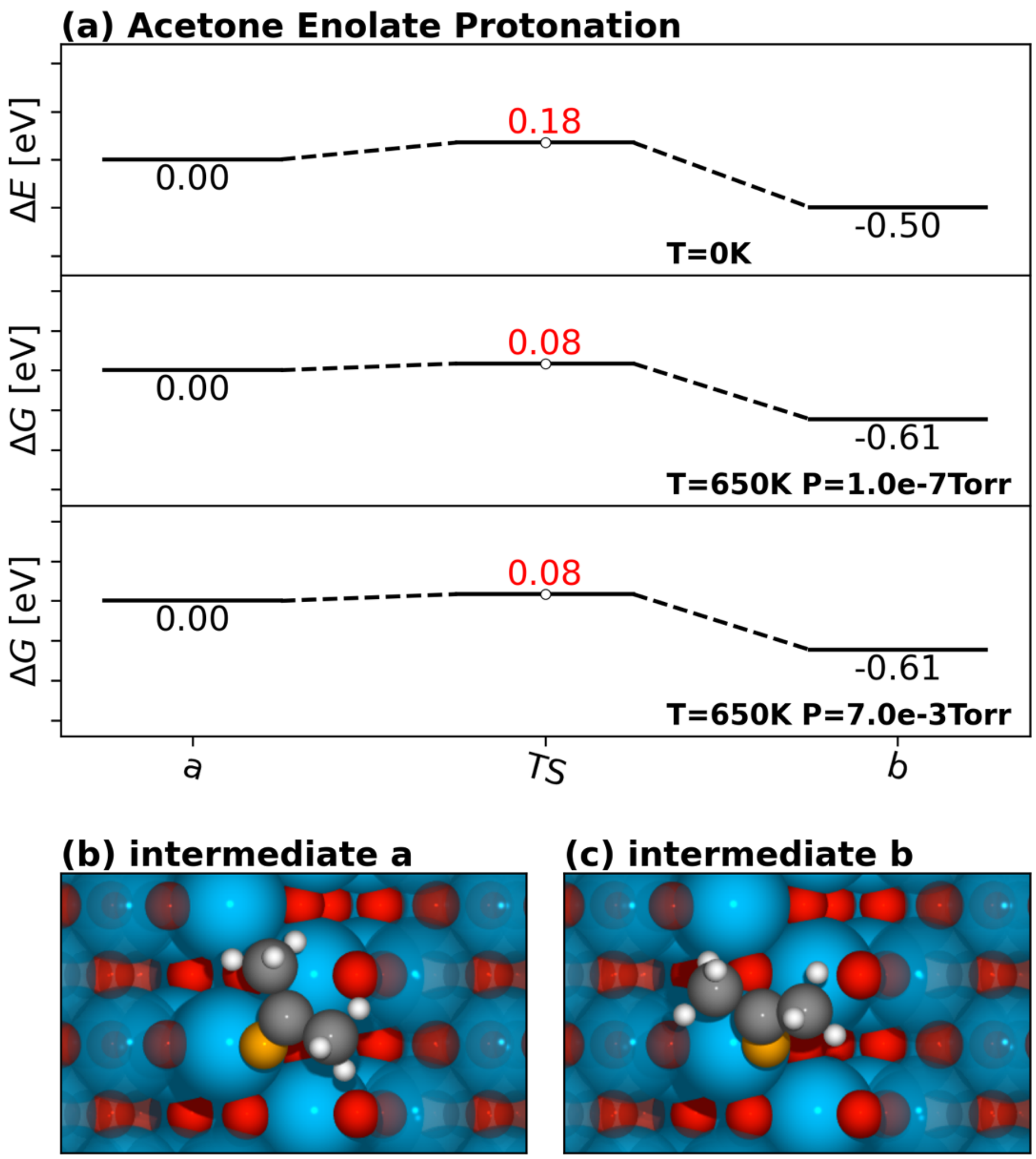

Supplementary Figure 23. (a) Total-energy (ΔE) and free-energy (ΔG) profiles for protonation of the acetone enolate at 0 K and at 650 K under 1×10^{-7} Torr and 7×10^{-3} Torr. Following decarboxylation of the deprotonated β-ketoacid, an acetone enolate is formed. The reactive CH_2 group of the enolate captures a surface H* to form acetone, with a total-energy barrier of +0.18 eV and a free-energy barrier of +0.08 eV at 650 K. (b) Structure of the acetone enolate formed following decarboxylation of the deprotonated β-ketoacid. (c) Structure of the final acetone product.

Supplementary Figure 24. Ketene coupling with an enolized bidentate acetate

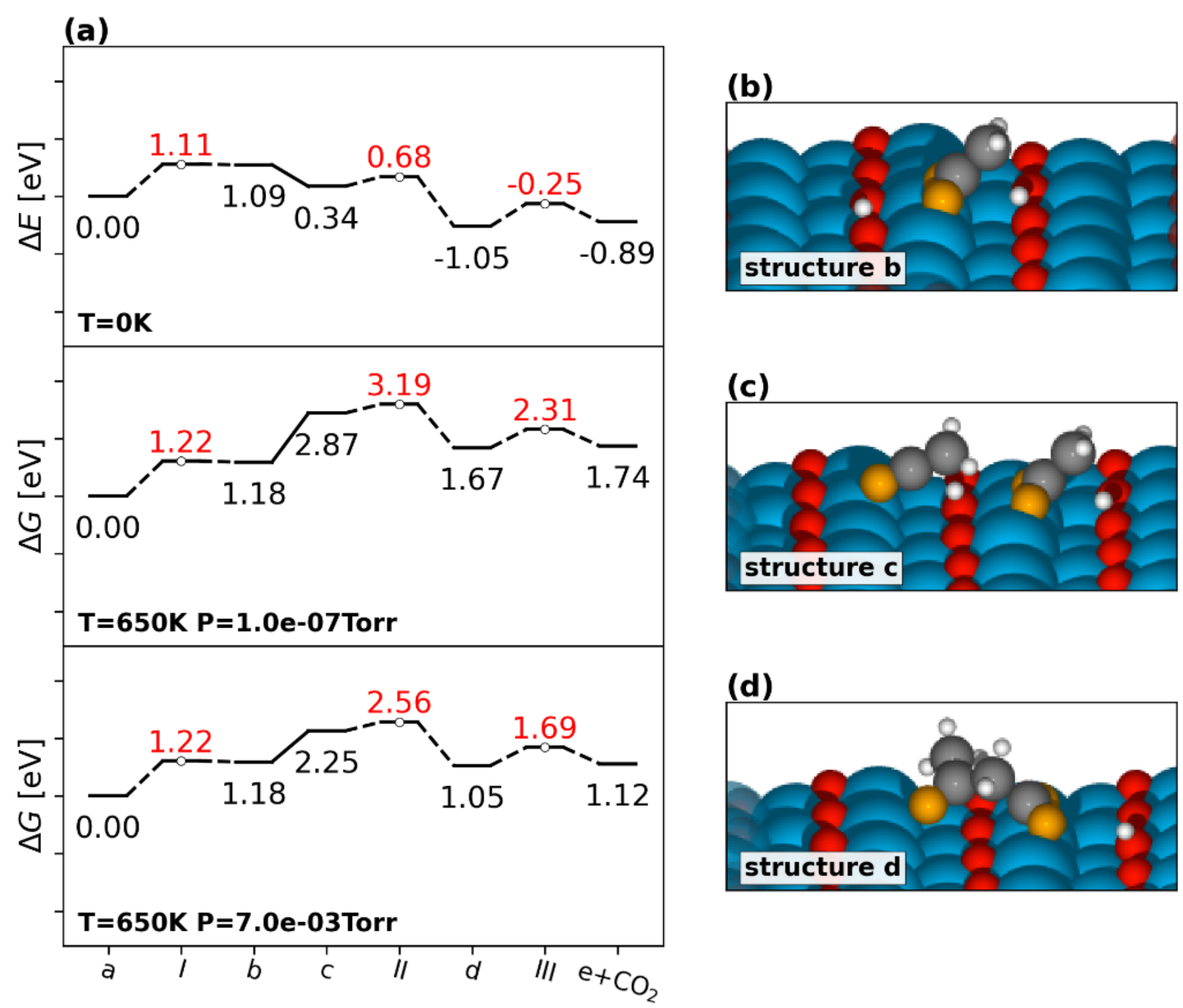

Supplementary Figure 24. (a) Total-energy (Δ*E*) and free-energy (Δ*G*) profiles for the reaction of ketene with a carboxylate formed by enolization of bidentate acetate, shown at 0 K and at 650 K under 1×10^{-7} Torr and 7×10^{-3} Torr. Enolization of bidentate acetate to form the reactive carboxylate intermediate (structure b) has a total-energy barrier of +1.11 eV. The resulting carboxylate couples with a nearby ketene (structure c), while the CH_2 group of ketene captures a surface H*, producing a deprotonated β-ketoacid (structure d). The C–C coupling step has a total-energy barrier of +0.34 eV, comparable to that for coupling between an enolate and monodentate acetic acid. Subsequent decomposition of the β-ketoacid produces an acetone enolate (structure e) and CO_2. Free-energy corrections have little effect on the surface reaction steps and primarily influence ketene adsorption from structure b to structure c. (b) Enolized bidentate acetate. (c) Ketene adsorbed in the vicinity of the bidentate enolate. (d) Deprotonated β-ketoacid formed following ketene–enolate coupling.

Supplementary References

1. Hofmann, S. *Auger- and X-Ray Photoelectron Spectroscopy in Materials Science: A User-Oriented Guide*. (Springer Berlin Heidelberg, 2012).

2. Patterson, A. L. The Scherrer Formula for X-Ray Particle Size Determination. *Phys Rev* **56**, 978–982 (1939).

3. O'Connor, C. R., *et al.* Insights into Acetic Acid Binding and Ketene Formation on Anatase TiO_2(101). *Top Catal* **66**, 1087–1101 (2023).

4. Herman, G. S., Dohnalek, Z., Ruzycki, N. & Diebold, U. Experimental investigation of the interaction of water and methanol with anatase-TiO_2(101). *J Phys Chem B* **107**, 2788–2795 (2003).

5. Hjorth Larsen, A., *et al.* The atomic simulation environment—a Python library for working with atoms. *J Phys: Condens Matter* **29**, 273002 (2017).

6. Kwon, S., Lin, T. C. & Iglesia, E. Elementary steps and site requirements in formic acid dehydration reactions on anatase and rutile TiO2 surfaces. *J Catal* **383**, 60–76 (2020).
