# Supplementary material for: Ketene as a gas phase intermediate in acetic acid ketonization on TiO2
Source: Nat Commun. 2026 Oct 28;17:11209. doi: 10.1038/s41467-026-77945-8 (PMC13720007; doi:10.1038/s41467-026-77945-8)
Supplement: Supplementary file 2 [file 41467_2026_77945_MOESM2_ESM.pdf]

nature portfolio

Peer Review File

Ketene as a Gas Phase Intermediate in Acetic Acid Ketonization on TiO_2

Corresponding Author: Dr Zdenek Dohnalek

This file contains all reviewer reports in order by version, followed by all author rebuttals in order by version.

Version 0:

Reviewer comments:

Reviewer #1

(Remarks to the Author)
This paper reported utilization of TiO2(101) crystallite instead of extended TiO2(101) surface to investigate ketonization of acetic acid. Using TPD and DFT, this paper demonstrated that ketene is a key intermediate for the formation of acetone, which was previously shown to be unlikely the intermediate. This paper provides interesting insight into the mechanism of ketonization. This paper can be acceptable after considering the following comments.

1. It is concluded that there is no oxygen vacancies on TiO2(101) through XPS and D2O-TPD. Other evidence such as EPR may be required to confirm this statement.
2. The adsorption mechanism of D2O is envisioned. The extending D2O to deeper layers at high doses is proposed. Please provide the direct evidence for the transport of molecules to inner layers.
3. In Figure 2c, the desorption behaviors of acetic acid and ketene are extremely similar. Therefore, it remains unclear that which specie (ketene or acetate) is response for the formation of acetone.
4. Is it possible to perform isotopic experiments to support the ketene as the intermediate for the formation of acetone?
5. In Figure 3, the ketene nucleophilically attack enolate using the C in carbonyl instead of the C in -CH2. However, the nucleophilic attack between enolate and monodentate acetic acid is occurred using the C in -CH2. Why ketene differs from enolate? Is it possible using the methylene C of ketene to attack the enolate carbonyl C (for example, ChemCatChem, 2025, 17(2), e202401442)? For the formation ketene, why not break the C-O bond of bidentate acetate first and then alfa-H abstraction?
6. The results in the paper support that ketene is an intermediate for the formation of ketone under low pressure condition. However, the extension of this conclusion to real reaction condition (high pressure, surface is completely covered by acetate) may be discussed.
7. Page 4, the authors stated that 'highlights the critical role of catalyst morphology in controlling catalytic selectivity.' However, this work does not appear to discuss the effect morphology, i.e., different exposed facets of TiO2 (such as, Catalysis Science Technology, 2023, 13, 5924-5937).
8. The DFT calculations indicate that pathways (b), (c), and (d) all converge to the same deprotonated β-ketoacid intermediate (h1, h2, and h3); however, the energy barriers for the subsequent decarboxylation step (Step VIII) exhibit variations across these different routes. While these discrepancies likely arise from conformational variations or differential coordination strengths on the TiO2(101) surface, the structural origins of these energy fluctuations are not explicitly addressed in the manuscript. The authors should clarify the underlying reasons for the discrepancies in the energy barriers and elucidate how the local environment facilitates the stabilization of the transition state during the decarboxylation process.

Reviewer #2

(Remarks to the Author)
Dohnalek and coworkers confirm that ketene acts as an intermediate in acetic acid ketonization. They use faceted TiO2(101) nanoparticles to assemble into layers with controlled thickness. It was shown that ketene formed from surface acetate can migrate through interparticle voids via desorption-adsorption events before reacting with surface acetate to yield acetone. Surface science, reactor studies and theory calculations reveal a direct correlation between ketene and acetone production. This work confirms that ketene acts as a mobile gas-phase intermediate in reaction. All the studies have been carried out

systematically and carefully. The conclusions are solid and of significance. It merits publication in Nature Commun. In addition, I have the following suggestions.
1) The assembling of TiO2 nanoparticles into layers has been used as the model surfaces. Based on the samples, TPD and other surface analyses have been carried out. The similar surface science studies in the single crystal TiO2 surfaces have been explored in previous works. Therefore, the difference and the new finding from the TiO2 particle surfaces could be discussed in more details. The TPD experiments over the assembled nanoparticles can be described in more detail in the main text. It is not clear how the authors perform the tests.
2) The reaction process they address in this work is not very common. To help the readers to understnad the process, the authors may presnt chemical reasction formula to illustate them.
3) The figure 3 seems to be complicdated and difficult to undertand. Could the authors simply it?

Version 1:

Reviewer comments:

Reviewer #1

(Remarks to the Author)
The authors have addressed most of my concerns. The paper is recommended for publication in current form.

Reviewer #2

(Remarks to the Author)
The authors have carefully addressed all comments and questions from the first reviewers and me. I recommend its publication in Natural Communications.

Open Access This Peer Review File is licensed under a Creative Commons Attribution 4.0 International License, which permits use, sharing, adaptation, distribution and reproduction in any medium or format, as long as you give appropriate credit to the original author(s) and the source, provide a link to the Creative Commons license, and indicate if changes were made.
In cases where reviewers are anonymous, credit should be given to 'Anonymous Referee' and the source.
The images or other third party material in this Peer Review File are included in the article's Creative Commons license, unless indicated otherwise in a credit line to the material. If material is not included in the article's Creative Commons license and your intended use is not permitted by statutory regulation or exceeds the permitted use, you will need to obtain permission directly from the copyright holder.
To view a copy of this license, visit https://creativecommons.org/licenses/by/4.0/

Response to Reviewer 1

Recommendation: Acceptable after considering the following comments.
This paper reported utilization of TiO_2(101) crystallite instead of extended TiO_2(101) surface to investigate ketonization of acetic acid. Using TPD and DFT, this paper demonstrated that ketene is a key intermediate for the formation of acetone, which was previously shown to be unlikely the intermediate. This paper provides interesting insight into the mechanism of ketonization. This paper can be acceptable after considering the following comments.

We thank the reviewer for their evaluation, constructive remarks, and their time. We address their specific points below.

1. It is concluded that there is no oxygen vacancies on TiO_2(101) through XPS and D_2O-TPD. Other evidence such as EPR may be required to confirm this statement.

Author reply: We thank the reviewer for this point. We agree that EPR would provide a sensitive complementary probe for determining presence of paramagnetic Ti^{3+}/oxygen vacancies in the bulk. We did not perform EPR in this study as we focused on determining the concentration of oxygen vacancies at the surface. Specifically, high-resolution scans of the Ti 2p region in the XPS spectra show only Ti^{4+} features, with no detectable Ti3+ shoulder at lower binding energy (Figure 1c). Further, D_2O TPD does not show high temperature recombinative desorption features (Refs 29, 30) that are characteristic of reduced or defective TiO_2 surfaces (Figure 1d). Combined, these methods represent both electronic and chemical probes of surface TiO_2 reduction and their results support the conclusion that oxygen vacancies are not present at detectable concentrations. Further, our findings are in accord with prior studies that showed that oxygen vacancies reside in the subsurface of anatase TiO_2(101) rather than at the surface and are therefore unlikely to dominate the chemistry discussed here.

Manuscript changes: We have revised the wording throughout the manuscript to avoid the absolute statement that there are "no oxygen vacancies." We have also added a caveat stating that EPR would be a useful complementary method for further quantifying trace vacancy populations that may exist in the bulk.

Page 6, §1
Trace bulk vacancies cannot be excluded without electron paramagnetic resonance (EPR), which would be a useful complementary measurement.

Page 7, §4
As with the planar surface, no desorption features are observed above 280 K, indicating that there is no significant concentration of surface defects such as oxygen vacancies on the faceted nanoparticles.

2. The adsorption mechanism of D_2O is envisioned. The extending D_2O to deeper layers at high doses is proposed. Please provide the direct evidence for the transport of molecules to inner layers.

Author reply: We thank the reviewer for this important comment. We agree that our original wording could be interpreted as claiming direct visualization of D_2O within buried particle layers. That was not our intent. We have revised the manuscript to state more clearly that D_2O transport into deeper layers is inferred from the dose- and thickness-dependent TPD behavior, rather than directly imaged.

The key experimental evidence comes from comparing D_2O uptake on nanoparticulate TiO_2(101) films of different thicknesses, as shown in **Supplementary Figure 5**. At low D_2O doses, the thick, medium, and thin films show similar behavior, consistent with adsorption occurring primarily on the outermost particle layers. At higher doses, however, the thinner films reach saturation, whereas the thick film continues to take up additional D_2O and does not saturate even at the highest doses examined. If D_2O adsorption were restricted only to the externally exposed particles, the thick film should saturate once those outer particles were filled, similarly to the thinner films. Instead, the continued increase in D_2O uptake with increasing dose and film thickness indicates that additional TiO_2 surface area becomes accessible in the thicker film, consistent with D_2O reaching particle layers below the external surface.

Additional support comes from the character of the TPD spectra. The desorption features remain centered at the same temperatures associated with D_2O bound to Ti_{5c} and O_{2c} sites, and the spectra increase mainly in intensity without the appearance of a multilayer water feature near ~175 K. Thus, the additional D_2O uptake is not due to condensation of multilayer water on the outer surface, but is instead consistent with adsorption on additional TiO_2 surface sites within the nanoparticulate layer.

Further, infrared reflection absorption spectroscopy measurement, **Supplementary Figure 6,** provides supporting spectroscopic evidence for similar acetate binding on planar and nanoparticulate TiO_2(101), while D_2O TPD remains the evidence for deeper-layer accessibility.

We have revised the text to clarify that this is self-consistent experimental evidence for transport through the nanoparticulate film, most likely by a combination of surface diffusion, desorption-readsorption, and gas-phase transport through interparticle voids.

Manuscript revisions:
We revised the discussion to clarify the basis for the proposed D_2O transport into deeper particle layers.

Page 7, §6 - 8, §1:
At low doses, where adsorption occurs primarily on the top layers of all films, the D_2O TPD spectra from the thin, medium, and thick nanoparticulate TiO_2(101) layers are similar. At higher doses, however, the thin and medium films approach saturation, whereas the thick film continues to take up additional D_2O. If D_2O adsorption were limited to the outermost particles exposed to the molecular beam, the thick film would also saturate once this external surface area was filled. Instead, the continued uptake by the thick film indicates that additional TiO_2 surface area becomes accessible at higher doses. Because the desorption features remain associated with D_2O bound to Ti_{5c} and O_{2c} sites and no multilayer water feature is observed near ~175 K, the increased uptake is not attributed to condensation of water on the external surface. Rather, the dose- and thickness-dependent uptake is consistent with D_2O transport into deeper regions of the nanoparticulate film, likely through a combination of surface diffusion, desorption-readsorption, and gas-phase transport through interparticle voids. [32]

Page 9, §3:
The vibrational frequencies revealing the binding of acetic acid on planar and nanoparticulate TiO_2(101) observed using infrared reflection absorption spectroscopy (IRRAS) are further shown in **Supplementary Figure 6**. There, dosing at very low temperature (25 K) leads to the molecular acetic acid layer and subsequent annealing above 150 K to deprotonation and bidentate acetate formation.[15]

Supplementary Figure 6:

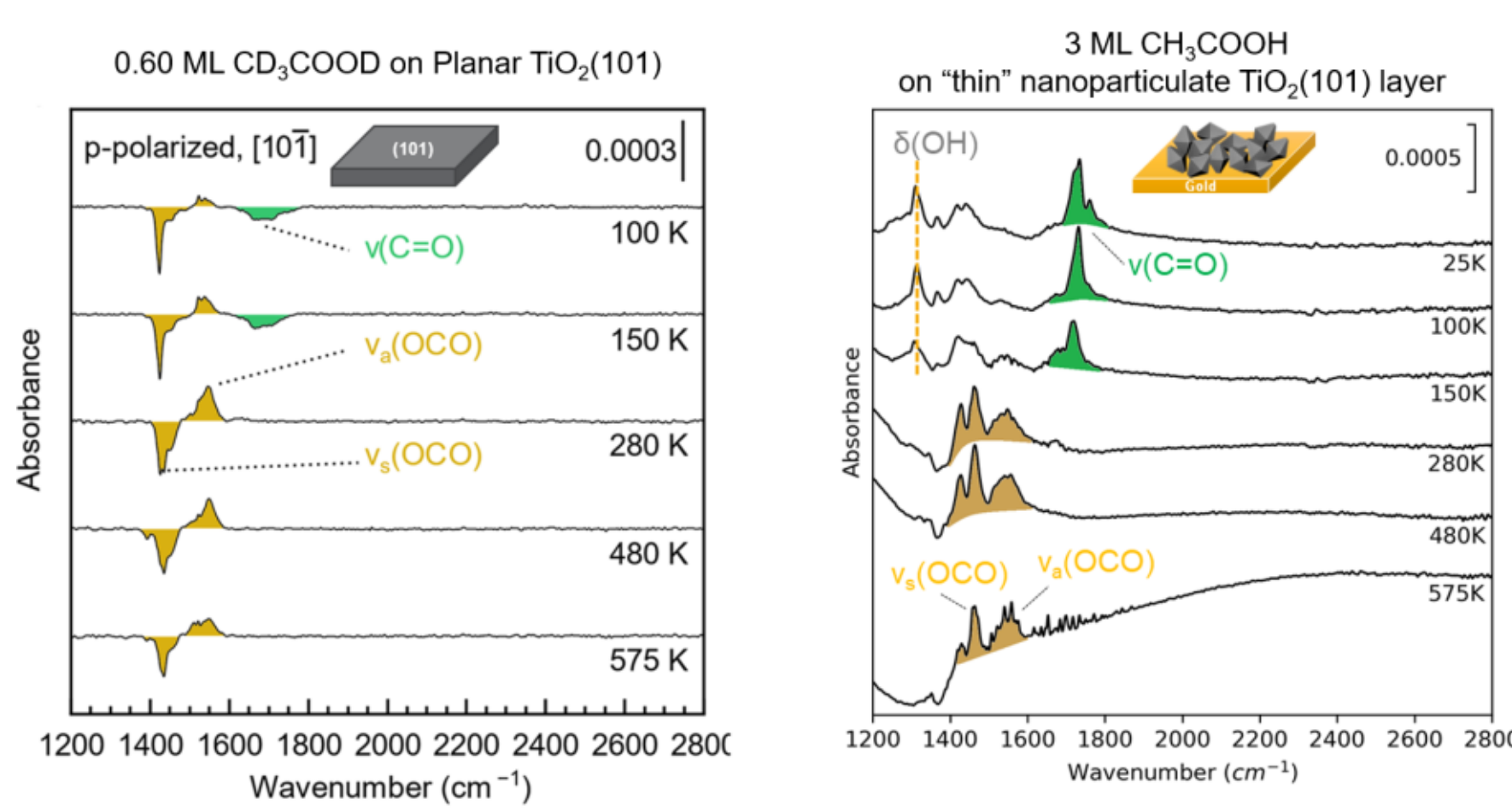

Figure 6. Infrared reflection absorption spectra probed with p-polarized light from 0.60 ML of CD_3COOD adsorbed on anatase TiO_2(101) [from Ref. 3] at 100 K (left) and 3 ML of CH_3COOH adsorbed on "Thin" layer of TiO_2(101) nanoparticles at 25 K (right). Each sample was flash annealed to 100, 150, 280, 480, and 575 K. The vibrational bands associated with bidentate acetate (yellow)

are ν_s(OCO) and ν_a(OCO) while the band associated with monodentate acetate (green) is ν(C = O). This indicates, that on nanoparticles, acetic acid has the same binding configuration as on anatase TiO_2(101) single crystal. Left panel reproduced with permission from Springer Nature. Christopher R. O'Connor et al., Insights into Acetic Acid Binding and Ketene Formation on Anatase TiO2(101), *Topics in Catalysis*, 66, 1087-1101, 2023, Springer Nature.

3. In Figure 2c, the desorption behaviors of acetic acid and ketene are extremely similar. Therefore, it remains unclear that which specie (ketene or acetate) is response for the formation of acetone.

Author reply: We thank the reviewer for raising this important mechanistic point. We agree that the desorption profiles of ketene and recombinatively formed acetic acid are similar over part of the temperature range, and we have revised the manuscript to clarify why we assign ketene as the more likely mobile intermediate involved in acetone formation.

Because the comment refers to the similar desorption behavior of acetic acid and ketene, we interpret the reviewer's question as concerning the identity of the gas-phase intermediate that reacts with the acetate-covered surface. Surface acetate itself is a necessary participant in acetone formation in all of the mechanisms considered in **Figures 3 and 4**. The key distinction is therefore whether the mobile species reacting with acetate-covered TiO_2 nanoparticles is ketene or recombinatively formed acetic acid.

Although ketene and recombinatively formed acetic acid show overlapping desorption features, their relationship to acetone formation differs. Recombinatively formed acetic acid is most prominent below ~600 K, where no acetone is observed, and its signal decreases above 600 K. Acetone, by contrast, appears mainly at higher temperatures, beginning near ~650 K, where the acetic acid signal is largely depleted. Ketene exhibits a broader desorption profile, including a high-temperature shoulder that overlaps more closely with the acetone desorption feature. Thus, the temperature dependence is more consistent with ketene, rather than recombinatively formed acetic acid, acting as the mobile gas-phase intermediate.

The dose dependence further supports this assignment. In **Figure 2c**, ketene and recombinatively formed acetic acid increase approximately linearly with acetic acid dose, whereas acetone appears only after the catalyst bed is saturated to greater depth and then increases nonlinearly. This delayed onset indicates that acetone formation depends on transport through the nanoparticle bed and repeated encounters of a mobile intermediate with acetate-covered surfaces. The batch-reactor data in **Figure 2d** are consistent with this picture: as the acetic acid dose increases, acetone grows more rapidly and eventually dominates, while the relative contribution of ketene decreases, as expected if ketene is consumed in secondary reactions that form acetone.

Finally, our DFT calculations support the chemical feasibility of ketene as the mobile reactant. The calculated mechanisms show that ketene can react with surface enolate/acetate-derived species through low-barrier pathways that converge on β-ketoacid intermediates leading to acetone. We have revised the discussion to make these arguments clearer.

Manuscript revisions: We revised the discussion to strengthen the argument that ketene, rather than recombinatively formed acetic acid, is the key mobile gas-phase intermediate.

Page 11, §2:
While recombinatively formed acetic acid is also observed in this temperature range, we argue that it is unlikely to be the principal mobile intermediate leading to acetone. This assignment is based on the relative temperature profiles of the products. Recombinatively formed acetic acid is most prominent below ~600 K, where no acetone is detected, and its yield decreases steadily at higher temperatures. Acetone desorption, in contrast, begins mainly near ~650 K, when recombinatively formed acetic acid desorption is largely complete. Ketene exhibits a broader desorption profile, with a high-temperature shoulder that overlaps closely with the acetone desorption feature. Together with the nonlinear dose dependence of acetone production in **Figure 2c** and the batch-reactor trends in **Figure 2d** (discussed below), these observations are most consistent with ketene, rather than recombinatively formed acetic acid, acting as the mobile gas-phase intermediate that reacts with acetate-covered TiO_2 nanoparticles to form acetone.

4. Is it possible to perform isotopic experiments to support the ketene as the intermediate for the formation of acetone?

Author reply: We agree with the reviewer that isotopic labeling experiments would provide a valuable additional test of the proposed ketene-mediated mechanism. In principle, the most direct experiment would be a sequential dosing experiment in which an acetate-covered $TiO_2(101)$ nanoparticle layer is first prepared using unlabeled acetic acid and then exposed to isotopically labeled ketene. Isotopic incorporation into acetone and/or CO_2 could then be monitored by isotope-resolved mass spectrometry. Such an experiment would directly test whether externally supplied ketene can enter the acetone-forming pathway. However, ketene is highly unstable and hazardous, and isotopically labeled ketene is not readily available as a reagent that can be introduced into our UHV dosing system.

We did perform isotopic labeling experiments using CD_3COOD, but these experiments revealed significant mass-spectral ambiguities that prevent an unambiguous mechanistic assignment using our quadrupole mass spectrometer

(**Supplementary Figure 14**). For example, the expected deuterated ketene product, CD_2CO, has a parent ion at m/z = 44, which overlaps with CO_2. In addition, fully deuterated acetone, CD_3COCD_3, and acetic acid, CD_3COOD, both give parent ions at m/z = 64. Thus, the key reaction channels cannot be reliably separated by mass spectrometry alone.

We also performed mixed dosing experiments using CH_3COOH and CD_3COOD on planar $TiO_2(101)$ to probe possible H/D exchange with surface hydrogen (not shown). However, these experiments were similarly complicated by isotopic overlaps. For example, CH_2DCOOH and CH_3COOD both appear at m/z = 61, while CD_2HCOOD and CD_3COOH both appear at m/z = 63. These overlaps make it difficult to distinguish isotopic scrambling from distinct reaction products.

To address the reviewer's suggestion, we have added a brief discussion of these isotopic labeling attempts to the manuscript and included the corresponding data in a new section of the Supporting Information. Although these experiments do not provide a definitive isotope-tracing test of the ketene intermediate because of the mass-spectral overlaps described above, they document the experimental constraints and explain why the current mechanistic assignment relies primarily on the temperature-dependent product profiles, dose-dependent acetone formation, batch-reactor trends, and DFT-calculated ketene reaction pathways.

Manuscript revisions:
We have added a brief description of the isotopic labeling experiments and their limitations to the manuscript. A new **Supplementary Figure 14** has been added that presents the CD_3COOD experiments and discusses the relevant mass-spectral overlaps.

Page 13, §1
Isotopic labeling experiments were also attempted to further test the proposed ketene-mediated pathway. However, the use of CD_3COOD leads to significant mass-spectral overlap among key products in our quadrupole mass spectrometer (see **Supplementary Figure 14**). In particular, CD_2CO has a parent ion at m/z = 44, overlapping with CO_2, while CD_3COCD_3 and CD_3COOD both give parent ions at m/z = 64. Mixed CH_3COOH/CD_3COOD dosing experiments (not shown) are also complicated by overlap among partially exchanged acetic acid isotopologues, including CH_2DCOOH/CH_3COOD at m/z = 61 and CD_2HCOOD/CD_3COOH at m/z = 63. These overlaps prevent an unambiguous isotope-tracing assignment under the present experimental conditions.

Supplementary Figure 14:

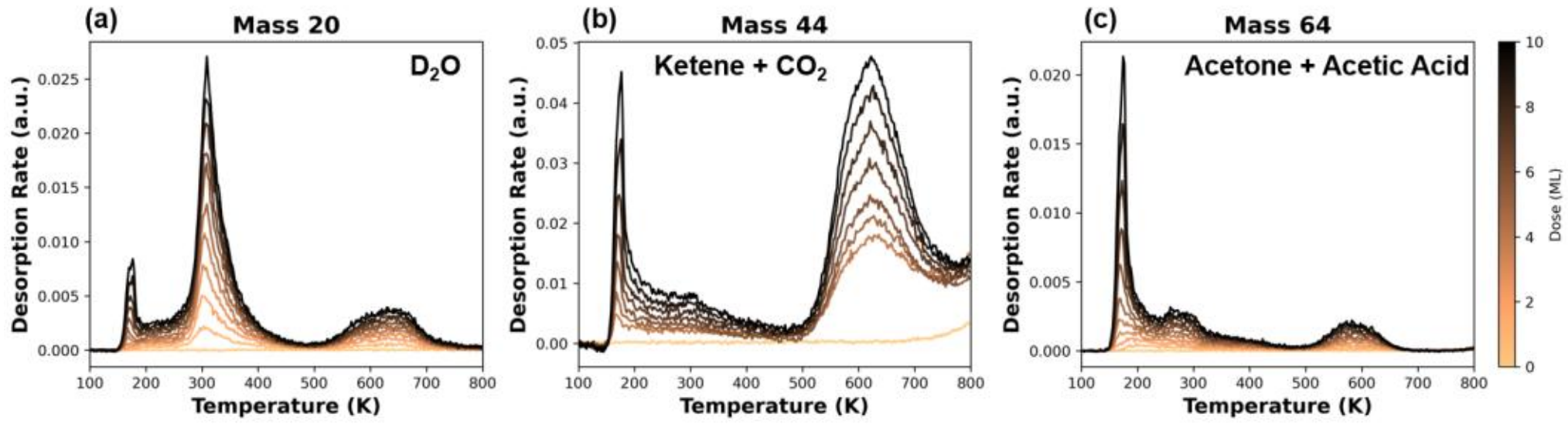

Figure 14. CD_3COOD coverage-dependent TPRS of D_2O, ketene and CO_2, and acetone and acetic acid from "medium" layer of $TiO_2(101)$ nanoparticles, obtained following a series of CD_3COOD doses at 100 K under UHV conditions. D-labeled ketene (CD_2CO) has the same mass as CO_2 at m/z = 44, and D-labeled acetone (CD_3COCD_3) has the same mass as D-labeled acetic acid (CD_3COOD) at m/z = 64.

5. In Figure 3, the ketene nucleophilically attack enolate using the C in carbonyl instead of the C in -CH2. However, the nucleophilic attack between enolate and monodentate acetic acid is occurred using the C in -CH2. Why ketene differs from enolate? Is it possible using the methylene C of ketene to attack the enolate carbonyl C (for example, ChemCatChem, 2025, 17(2), e202401442)? For the formation ketene, why not break the C-O bond of bidentate acetate first and then alfa-H abstraction?

Author reply: The two C–C coupling pathways shown in **Figure 3** involve (i) monodentate acetic acid reacting with a monodentate enolate and (ii) ketene reacting with a monodentate enolate. To clarify the origin of the nucleophilic and electrophilic sites in these reactions, we analyzed the Hirshfeld charges of the corresponding structures, namely structures (f) and (k) in **Figure 3**. In both cases, the methylene carbon ($–CH_2$) of the enolate carries a substantial negative charge (−0.90 e in (f) and −0.89 e in (k)), identifying it as the nucleophilic center. Meanwhile, the carbonyl carbon of monodentate acetic acid and ketene is positively charged (+0.54 e in (f) and +0.39 e in (k)), acting as the electrophilic center. Therefore, for both reactions, the C–C coupling proceeds via nucleophilic attack of the enolate methylene carbon onto the carbonyl carbon of either monodentate acetic acid or ketene. The directions of the nucleophilic attack are shown in **Figure 3**.

The methylene carbon ($-CH_2$) of ketene also carries a negative charge (−0.89 e in (k)), indicating its nucleophilic character. If ketene were to attack the carbonyl carbon of acetic acid via its methylene carbon, this would yield adsorbed $COCH_2CH_3COOH$, which has a positive charge localized on the carbonyl carbon of ketene. The cation $COCH_2CH_3COOH$ cannot be stabilized on $TiO_2(101)$ surface in the absence of electron holes. Therefore, the nucleophilic attack of ketene via its methylene carbon on the carbonyl carbon of acetic acid is highly unlikely in our system. The C–C coupling mechanism in zeolite proposed in ChemCatChem 2025 (Scheme 2 and Figure 6), as suggested by the reviewer,

involves the reaction between ketene and an acylium cation, where the methylene carbon of ketene attacks the electrophilic carbonyl carbon of the acylium species. However, the formation of an adsorbed acylium group from acetic acid on $TiO_2(101)$ has a high energy barrier of ~1.0 eV, which has been reported in **Supplementary Figure 20** step I. Moreover, the acylium cation cannot be stabilized on the $TiO_2(101)$ surface without the presence of electron holes. As a result, the reaction pathway involving ketene acting as the nucleophile toward an acylium species is unlikely under our reaction conditions.

Regarding ketene formation, a mechanism involving initial C–O bond cleavage of bidentate acetate would generate an acylium cation and an adsorbed oxygen atom, which cannot be stabilized on the $TiO_2(101)$ surface without electron holes. Instead, we found a pathway starting from a monodentate acetic acid, where an adsorbed acylium group is formed via C-O bond cleavage followed by α-H abstraction to yield ketene. However, the two reaction steps involved in this pathway both have high energy barriers (~1.0 eV for C-O bond cleavage and 1.3 eV for α-H abstraction), making it kinetically unfavorable compared to the α-H abstraction pathway directly from monodentate acetic acid discussed in the manuscript. This pathway has also been reported in **Figure S20**.

6. The results in the paper support that ketene is an intermediate for the formation of ketone under low pressure condition. However, the extension of this conclusion to real reaction condition (high pressure, surface is completely covered by acetate) may be discussed.

Author reply: We agree with the reviewer. We have revised the manuscript to avoid overextending the UHV results and to more carefully distinguish between elementary-step evidence and steady-state catalytic operation.
Our UHV experiments are designed to isolate elementary processes and demonstrate that ketene can function as a mobile intermediate when produced within a nanoparticulate $TiO_2(101)$ layer. Under ambient and catalytic conditions, the surface coverage, residence time, and relative abundance of bidentate acetate, monodentate acetate/acetic acid, and enolate species will differ. We therefore do not claim that ketene is the only acetone-forming intermediate under all reactor conditions. Instead, we conclude that ketene-mediated C-C coupling is a viable and potentially important pathway that can operate in parallel with the classical β-ketoacid mechanism.

Manuscript revisions:
Page 4, §2
However, we emphasize that our UHV experiments are designed to isolate elementary steps and transport effects rather than reproduce the full steady-state population of surface species. Under more realistic conditions, the relative populations of bidentate acetate, monodentate acetate/acetic acid, enolate species, and weakly adsorbed intermediates may differ from those in the temperature-programmed experiments. As such, our results do not rule out the classical β-

ketoacid mechanism or establish ketene as the only acetone-forming intermediate. Rather, they demonstrate that ketene-mediated C–C coupling is a viable pathway for the ketonization of acetic acid.

Page 11, §1
Although yields under UHV conditions are modest (~1%) due to limited residence time, shallow particle layer depth, and low collision probability, the systematic increase in acetone formation with dose and layer thickness demonstrates that ketene-mediated chemistry is accessible under the conditions studied. At higher pressures and in thicker catalyst beds, longer residence times and more frequent surface encounters are expected to increase the contribution of this pathway. However, its quantitative contribution under steady-state catalytic conditions will depend on the surface coverage and relative abundance of acetate, monodentate acetic acid, and enolate species.

Page 13, §3
This progression is consistent with a ketene-mediated contribution to acetone formation and supports the mechanistic picture developed from the UHV studies on nanoparticulate TiO_2(101) layers. We note that these batch-reactor TPRS experiments still probe transient conditions after evacuation rather than true steady-state catalytic operation, and therefore they are best interpreted as evidence for the feasibility of ketene-mediated coupling rather than as a quantitative measure of its contribution under high-pressure reaction conditions.

7. Page 4, the authors stated that 'highlights the critical role of catalyst morphology in controlling catalytic selectivity.' However, this work does not appear to discuss the effect morphology, i.e., different exposed facets of TiO2 (such as, Catalysis Science Technology, 2023, 13, 5924-5937).

Author reply: We thank the reviewer for pointing out this ambiguity. In our manuscript, "morphology" referred primarily to the "mesoscopic" architecture of the catalyst layer, rather than to a systematic comparison of exposed crystal facets. We have replaced the word "morphology" with "architecture".

We have also added a brief description noting that facet-dependent ketonization activity on anatase TiO_2 has been reported in a Catalysis Science & Technology 2023 study cited by the reviewer:

Page 3, §2
Furthermore, facet-dependent studies of anatase TiO_2 suggest that the monodentate binding configuration of carboxylic acids is likely the more reactive intermediate toward C–C coupling.[14]

8. The DFT calculations indicate that pathways (b), (c), and (d) all converge to the same deprotonated β-ketoacid intermediate (h1, h2, and h3); however, the energy barriers for the

subsequent decarboxylation step (Step VIII) exhibit variations across these different routes. While these discrepancies likely arise from conformational variations or differential coordination strengths on the TiO2(101) surface, the structural origins of these energy fluctuations are not explicitly addressed in the manuscript. The authors should clarify the underlying reasons for the discrepancies in the energy barriers and elucidate how the local environment facilitates the stabilization of the transition state during the decarboxylation process.

Author reply: We thank the reviewer for this insightful comment. Although pathways (b), (c), and (d) converge to the same deprotonated β-ketoacid chemical intermediate, the resulting adsorbates are not identical on the TiO_2(101) surface. As shown in the newly added **Supplementary Figure 21**, h1, h2, and h3 adopt distinct adsorption geometries inherited from the preceding C-C coupling steps, which lead to different transition-state structures for the subsequent decarboxylation step. The small variations in the calculated activation barriers are therefore attributed to these geometric differences. We have added corresponding discussion in the revised manuscript and **Supplementary Figure 21** in the revised supporting information to clarify the structural origins of these barrier variations.

Manuscript revisions:

Page 18, §3:
Although the three pathways lead to the same chemical intermediate, the resulting surface species (h1, h2, and h3) adopt distinct adsorption geometries inherited from the preceding C–C coupling and proton-transfer steps. As shown in **Supplementary Figure 21**, these geometric differences give rise to slightly different transition-state structures for the subsequent decarboxylation step, resulting in modest variations in the calculated activation barriers (+0.89, +0.93, and +0.88 eV for h1, h2, and h3, respectively).

Supplementary Figure 21:
Figure 21 shows the deprotonated β-ketoacid intermediates (h1–h3) and the corresponding decarboxylation transition states. While the three pathways converge to the same chemical intermediate, the adsorbates adopt distinct adsorption geometries inherited from the preceding reaction steps. These geometric differences lead to slightly different transition-state structures and account for the small variations in the calculated decarboxylation barriers (0.89–0.93 eV).

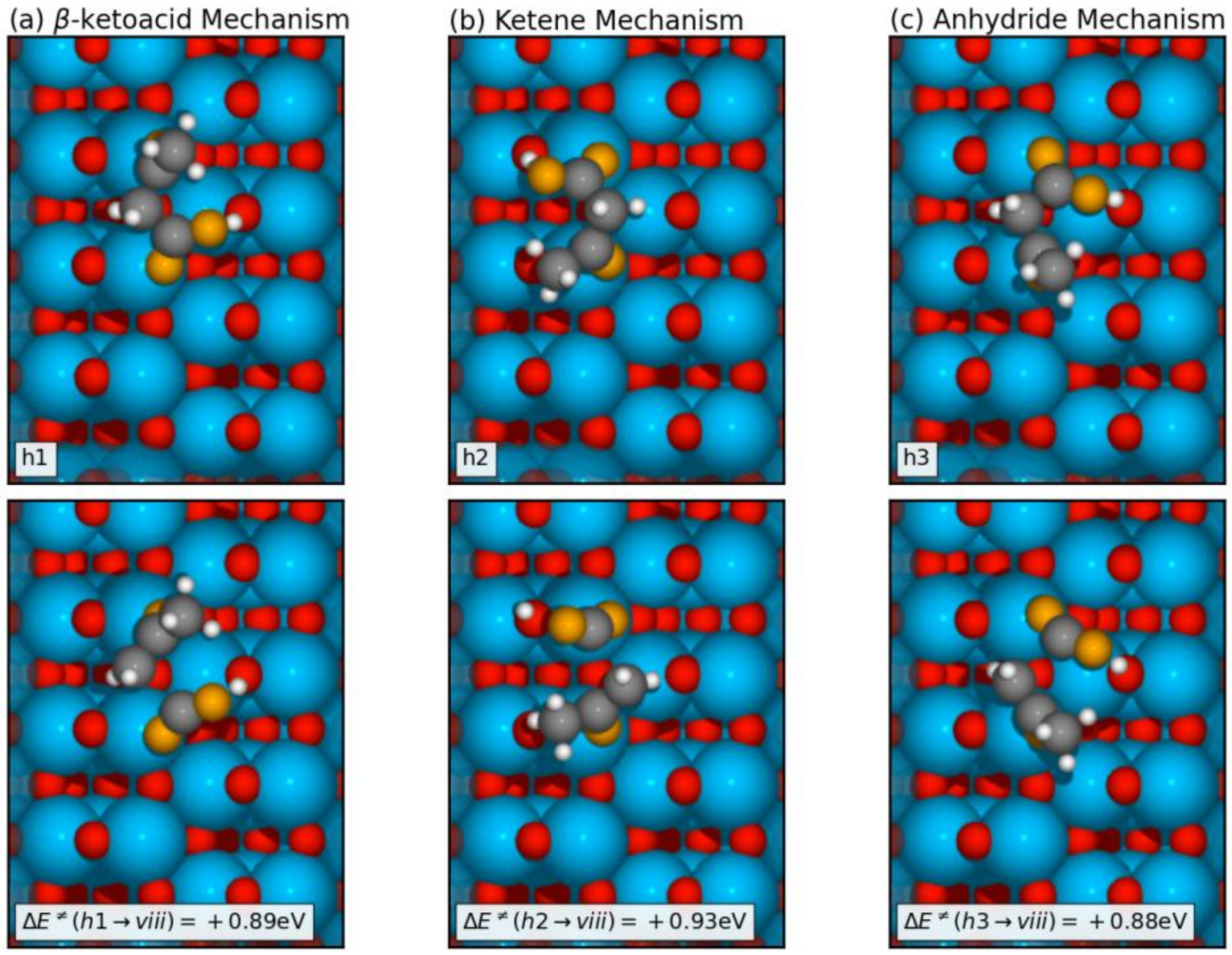

Figure 21. Optimized β-ketoacid and transition-state structures for the decarboxylation step through different reaction pathways. Panels (a–c) correspond to the β-ketoacid, ketene, and anhydride mechanisms, respectively, yielding intermediates h1, h2, and h3 prior to decarboxylation. The calculated activation barriers are shown in the lower left of each panel.

Response to Reviewer 2

Recommendation: Merits publication in Nature Communications
Dohnalek and coworkers confirm that ketene acts as an intermediate in acetic acid ketonization. They use faceted TiO2(101) nanoparticles to assemble into layers with controlled thickness. It was shown that ketene formed from surface acetate can migrate through interparticle voids via desorption-adsorption events before reacting with surface acetate to yield acetone. Surface science, reactor studies and theory calculations reveal a direct correlation between ketene and acetone production. This work confirms that ketene acts as a mobile gas-phase intermediate in reaction. All the studies have been carried out systematically and carefully. The conclusions are solid and of significance. It merits publication in Nature Commun.

We thank the reviewer for their evaluation, constructive remarks, and their time. We address their specific points below.

In addition, I have the following suggestions:

1. The assembling of TiO2 nanoparticles into layers has been used as the model surfaces. Based on the samples, TPD and other surface analyses have been carried out. The similar surface science studies in the single crystal TiO2 surfaces have been explored in previous works. Therefore, the difference and the new finding from the TiO2 particle surfaces could be discussed in more details. The TPD experiments over the assembled nanoparticles can be described in more detail in the main text. It is not clear how the authors perform the tests.

We thank the reviewer for this helpful comment. We agree that the distinction between the planar TiO_2(101) single-crystal studies and the present assembled nanoparticle-layer experiments should be made clearer in the manuscript. The assembled nanoparticle layers introduce a three-dimensional porous architecture containing interparticle voids and multiple particle layers that is absent on the planar TiO_2(101) model. This architecture enables transport and repeated surface encounters of volatile intermediates, which cannot occur on a planar single crystal. This enables us to study the role of reactive gas phase intermediates.

We have revised the manuscript to emphasize this distinction more clearly. We have also expanded the description of the nanoparticle-layer TPD/TPRS experiments in the main text. Specifically, we now state how the nanoparticle films were deposited on Au-coated Ta substrates, how they were heated and temperature-calibrated, how they were cleaned prior to dosing, how D_2O and acetic acid were introduced using the molecular beam, how coverages were defined in ML-equivalents relative to planar TiO_2(101), and how TPD/TPRS spectra were collected.

Manuscript Revisions:
Page 8, §4 – page 9, §1:
The comparison with planar $TiO_2(101)$ is central to the interpretation of these experiments. The D_2O TPD results show that the faceted nanoparticles expose $TiO_2(101)$-like adsorption sites, as indicated by desorption features associated with Ti_{5c} and O_{2c} sites. However, the assembled nanoparticle layers differ from planar $TiO_2(101)$ in two important ways. First, they provide a much larger accessible surface area distributed through multiple particle layers. Second, the interparticle voids create a three-dimensional transport network in which weakly bound or volatile species can undergo desorption-readsorption events and collide with neighboring particle surfaces before escaping into vacuum. Thus, the nanoparticle layers retain the local surface chemistry of $TiO_2(101)$, while introducing transport and spatially separated reaction steps that are absent on a planar single crystal. In this respect, the nanoparticle layers more closely match the conditions encountered in real catalytic reactors than the single crystal experiments.

Page 22, §2:
For nanoparticle-layer experiments, faceted $TiO_2(101)$ nanoparticles were deposited onto optically polished Au-coated Ta substrates by spray-casting or drop-casting suspensions of the nanocrystals. The resulting films were mounted in the same UHV TPD/TPRS apparatus used for the planar $TiO_2(101)$ single crystal. The samples were resistively heated through the Ta support, and the temperature was monitored using a K-type thermocouple attached to the back of the Ta plate. The nanoparticle temperature calibration was checked using the D_2O desorption peak relative to planar $TiO_2(101)$. Prior to dosing, the nanoparticle films were heated to 900 K to remove adsorbed species and annealed in an O_2 beam; cleanliness was confirmed by the absence of desorption products in a blank TPD experiment. D_2O and CH_3COOH were dosed with a molecular beam at controlled sample temperatures, and TPD/TPRS spectra were recorded during linear heating at 1 K/s. Coverages on nanoparticle samples are reported as ML-equivalents referenced to the Ti_{5c} site density of planar $TiO_2(101)$; therefore, 1 ML-equivalent does not correspond to saturation of all active sites within the nanoparticle bed.

2. The reaction process they address in this work is not very common. To help the readers to understand the process, the authors may present chemical reaction formula to illustrate them.

Author reply: We thank the reviewer for this suggestion. To facilitate understanding of the reaction process, we have revised **Figure 3**, added chemical formulas of the adsorbed intermediates and products, and provided the overall chemical equations in the revised text. These annotations provide a clearer connection between the surface structures and the corresponding chemical transformations, making the overall reaction pathways easier to follow.

Manuscript revisions:
Revised Figure 3:

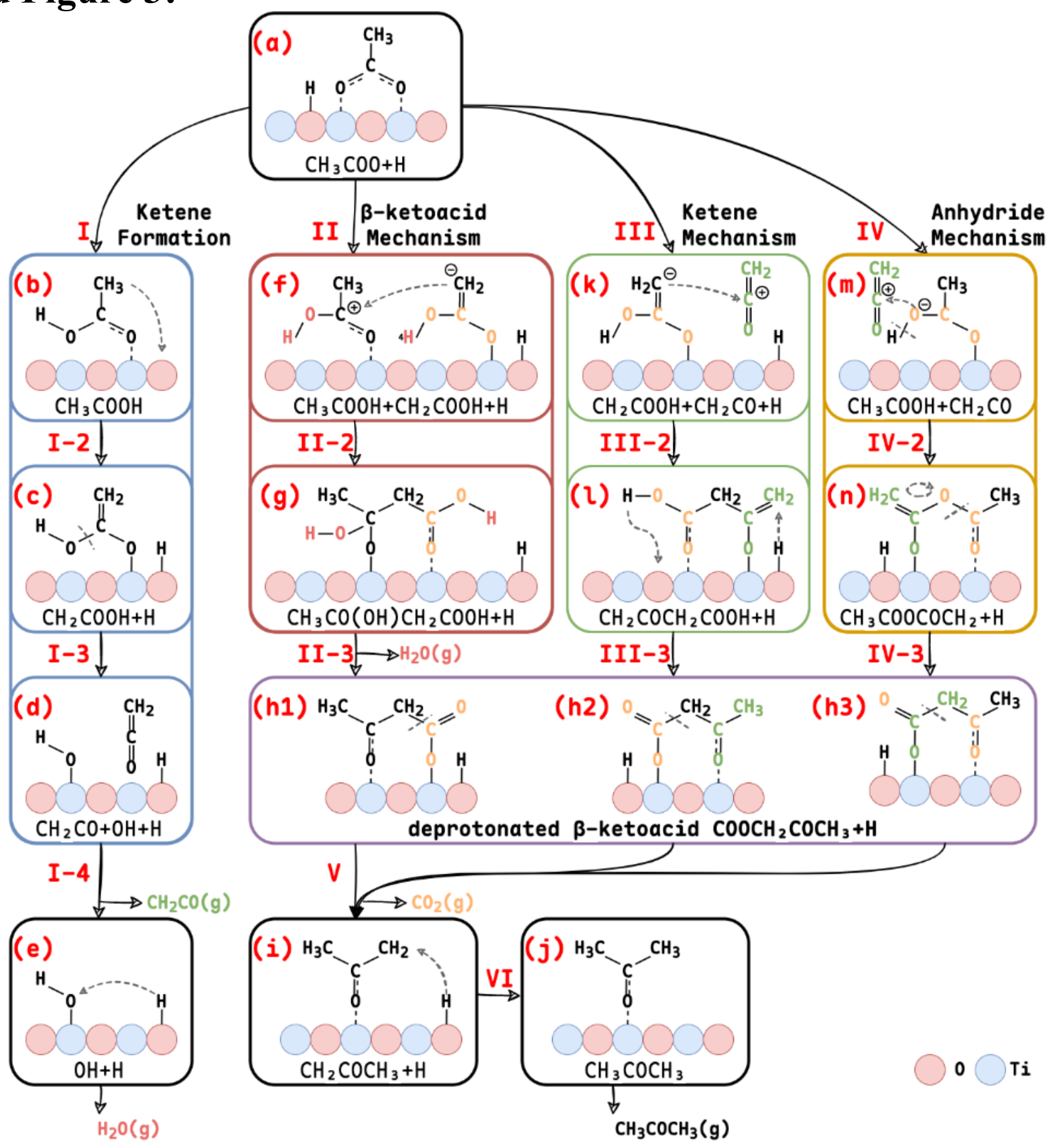

Figure 3. Proposed reaction network for ketene (CH_2CO) and acetone (CH_3COCH_3) formation from acetic acid (CH_3COOH) on anatase $TiO_2(101)$. All pathways originate from bidentate acetate (a). Pathway I describes ketene formation via enolization and subsequent decomposition through intermediates b–e, corresponding to the overall reaction $CH_3COOH \rightarrow CH_2CO + H_2O$. Acetone can form through three distinct mechanisms that converge to a common deprotonated β-ketoacid intermediate (h1–h3). In the β-ketoacid mechanism (Pathway II), a monodentate acetic acid species couples with a 1-hydroxyl enolate through intermediates f–g, leading overall to $2CH_3COOH \rightarrow CH_3COCH_3 + CO_2 + H_2O$. In the ketene mechanism (Pathway III), ketene reacts with a 1-hydroxyl enolate through intermediates k–l, corresponding to the overall reaction $CH_2CO + CH_2COOH + H \rightarrow CH_3COCH_3 + CO_2$. In the anhydride mechanism (Pathway IV), ketene reacts with a monodentate acetic acid species to form an anhydride intermediate through intermediates m–n, corresponding to $CH_2CO + CH_3COOH \rightarrow CH_3COCH_3 + CO_2$. The resulting β-ketoacid intermediates subsequently undergo decarboxylation (step V) and proton transfer (Step VI) to form acetone. The key nucleophilic attack steps are illustrated in structures f, k, and m.

Page 14, §2:

The proposed reaction network is summarized in **Figure 3**. Ketene formation (**mechanism I**) proceeds via conversion of bidentate acetate (structure **a**) to monodentate acetic acid (**b**), followed by enolization and decomposition of the resulting 1-hydroxy enolate. The net reaction is $CH_3COOH \rightarrow CH_2CO + H_2O$. On the other hand, acetone formation can occur via three mechanistic pathways. The widely accepted *β-ketoacid mechanism* (**mechanism II**) involves C–C coupling between a monodentate acetic acid and a 1-hydroxy enolate derived from acetate (**step II-2**),[10] ultimately yielding acetone via the overall reaction $2CH_3COOH \rightarrow CH_3COCH_3 + CO_2 + H_2O$. In the second route, termed the *ketene mechanism* (**mechanism III**), ketene couples with a surface-bound 1-hydroxy enolate (**step III-2**). The overall process is $CH_2CO + CH_2COOH + H \rightarrow CH_3COCH_3 + CO_2$. The third route, *anhydride mechanism* (**mechanism IV**), proceeds via the formation of a surface anhydride intermediate from the reaction of ketene with monodentate acetic acid (**step IV-2**), corresponding to $CH_2CO + CH_3COOH \rightarrow CH_3COCH_3 + CO_2$. Although these three mechanisms are often treated as parallel,[8] our calculations reveal that they all converge to a common deprotonated β-ketoacid intermediate (**h1**, **h2**, and **h3**).

3. The figure 3 seems to be complicdated and difficult to undertand. Could the authors simply it?

Author reply: We thank the reviewer for this suggestion. To improve readability, **Figure 3** has been substantially simplified and reorganized. The reaction network is now divided into four color-coded pathways: ketene formation, β-ketoacid mechanism, ketene mechanism, and anhydride mechanism. Only the key intermediates associated with the reaction-energy profiles are retained, and chemical formulas are added to each intermediate to facilitate interpretation. The revised figure provides a clearer overview of the relationship among the four pathways while preserving the mechanistic details discussed in the manuscript.
